# Integrated Mendelian Randomization and Single‐Cell Transcriptomics Analysis Identifies Critical Blood Biomarkers and Potential Mechanisms in Epilepsy

**DOI:** 10.1111/cns.70172

**Published:** 2025-01-03

**Authors:** Jianwei Shi, Jing Xie, Yanfeng Yang, Bin Fu, Zuliang Ye, Ting Tang, Quanlei Liu, Jinkun Xu, Penghu Wei, Yongzhi Shan, Guoguang Zhao

**Affiliations:** ^1^ Department of Neurosurgery Xuanwu Hospital, Capital Medical University Beijing China; ^2^ China International Neuroscience Institute Beijing China; ^3^ Deanery of Biomedical Sciences, Edinburgh Medical School, College of Medicine and Veterinary Medicine University of Edinburgh Edinburgh UK; ^4^ National Clinical Research Center for Geriatric Disorders Xuanwu Hospital, Capital Medical University Beijing China; ^5^ Department of Neurosurgery First Hospital of Shanxi Medical University Taiyuan China

**Keywords:** epilepsy, key genes, mendelian randomization, risk incidence, single‐cell transcriptomics

## Abstract

**Background:**

Epilepsy has a genetic predisposition, yet causal factors and the dynamics of the immune environment in epilepsy are not fully understood.

**Methods:**

We analyzed peripheral blood samples from epilepsy patients, identifying key genes associated with epilepsy risk through Mendelian randomization, using eQTLGen and genome‐wide association studies. The peripheral immune environment's composition in epilepsy was explored using CIBERSORT. An epilepsy mouse model was established to validated the expression of key genes at the transcriptomic and proteomic levels through single‐cell analysis. Relevant pathways were verified. Finally, we developed a predictive model for antiepileptic drug response in epilepsy patients.

**Results:**

We found that *CDC25B*, *DNMT1*, *GZMA*, *MTX1*, and *SSH2* expression decreases epilepsy risk, whereas *FGD3*, *RAF1*, and *SH3BP5L* increase it. Epilepsy patients exhibited an altered peripheral immune profile, notably with increased activated mast cells and decreased CD4 memory activated T cells and γδ T cells. Eight genes were significantly related to this immune environment. In the animal model, *FGD3*, *SSH2*, and *DNMT1* were upregulated at both mRNA and protein levels in the hippocampus. FGD3 and SSH2 are specifically elevated in microglia and are primarily associated with actin regulation. The trained predictive model was deployed on an online platform.

**Conclusions:**

This study elucidates key genes linked to epilepsy, delineates the epilepsy immune landscape, and highlights the interaction between these domains, providing insights into potential epilepsy mechanisms and treatments.

## Graphical abstract

This study identified eight key genes associated with epilepsy risk through Mendelian randomization analysis, analyzed the peripheral immune composition of epilepsy patients and the link with these genes, and validated the expression and function of the eight genes in the central system using animal models and single‐cell analysis. (A) Bioinformatic Analysis & Mendelian Randomization illustrates data processing from GEO, eQTLGen, GWAS to key gene identification. (B) Immune microenvironment details immune cell profiling, infiltration analysis, and intercellular correlation within the immune system. (C) Experimental Validation presents in vivo model construction (KA‐induced epileptic mice) and in vitro RNA/protein analysis (qPCR, Western blot, and immunofluorescence). (D) Single‐Cell highlights scRNA‐seq for gene identification. (GEO, Gene Expression Omnibus; eQTLGen, expression quantitative trait loci; GWAS genome‐wide association studies; KA, kainic acid; qPCR, quantitative polymerase chain reaction; scRNA‐seq, single cell RNA sequence).

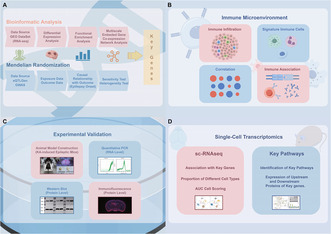

## 1 Introduction

Epilepsy, ranked as the fourth most common neurological disorder, affects approximately 70 million individuals worldwide [1]. Epileptogenesis, the gradual process leading to epilepsy, is categorized into acute, subacute, and chronic phases based on morphological alterations and molecular characteristics [2]. Roughly one‐third of epilepsy patients progress to the chronic phase, culminating in drug‐resistant epilepsy (DRE) [1]. Individuals with DRE do not respond to treatment with two or more antiseizure medications and may only find potential relief through resective epilepsy surgery. However, the ongoing occurrence of recurrent unprovoked seizures and progressive cognitive decline remains a challenging issue for epilepsy patients. The economic impact of epilepsy extends beyond healthcare systems, imposing a significant personal burden on affected individuals [3]. Furthermore, surgical resection is constrained by the necessity to precisely identify and remove singular, non‐essential functional areas of seizure‐causing brain tissue, with the risk of postoperative neurological and psychiatric impairments [4]. Hence, there is a critical need to delve deeper into the biological mechanisms underlying epilepsy.

Complex molecular mechanisms, such as abnormal synaptic transmission and neurotransmitter imbalances, are crucial in epilepsy pathophysiology [5, 6]. Both presynaptic and postsynaptic changes drive persistent epileptic activity. Neurotransmitter imbalances, marked by increased glutamate and decreased GABA, heighten neuronal excitability and seizures. Inflammatory mediators like IL‐1β and TNF‐α further enhance excitatory transmission and reduce inhibitory transmission. Chronic seizures cause neuroplastic changes and genetic mutations, reinforcing excitability and seizure susceptibility. Mutations in genes related to synaptic transmission, ion channels, and inflammation contribute to epilepsy's chronicity. Immune activation and chronic inflammation disrupt the blood–brain barrier, perpetuating neuroinflammatory cycles. Understanding these intricate molecular mechanisms is crucial for developing novel therapeutic strategies for refractory epilepsy.

Large‐scale genomics and transcriptomics studies have significantly advanced our understanding and treatment of epilepsy. Transcriptomic analyses have identified specific gene expression changes linked to DRE, offering insights into its molecular basis [7]. Precision medicine for epilepsy focuses on customizing treatments based on a patient's unique genetic profile, improving therapeutic outcomes. Genomic‐guided approaches can detect drug‐resistant mutations and predict responses to antiepileptic drugs [8]. Additionally, personalized strategies, like targeted gene therapies, are being developed for epilepsy‐specific genetic alterations [9]. Mendelian randomization is increasingly used to identify causal relationships between genetic variants and disease risk factors. This method leverages genetic variants as instrumental variables to infer causality, providing robust evidence for potential therapeutic targets and risk factors in epilepsy [10].

Epilepsy progresses over time, marked by anatomical alterations such as neuronal loss, changes in synaptic plasticity, gliosis, and network reorganization [11, 12]. Numerous biomarkers, particularly characteristic genes across various spatial and temporal contexts, serve as objective measures within these biological processes, advancing the diagnosis, prognosis, and treatment of epilepsy. These genes not only influence the clinical manifestations of epilepsy through diverse molecular functions, revealing different disease subtypes, but are also closely linked to the genetic predisposition to epileptic seizures [12, 13]. Given that genotypic formation occurs before disease onset and is generally unaffected by disease progression, coupled with the high heritability of epilepsy, identifying key genes with a causal relationship to epilepsy risk from a genetic variation perspective will offer valuable insights for clinical practice and mechanistic exploration.

To elucidate the genetic and immunological features of epilepsy, we identified differentially expressed genes (DEGs), applied multiscale embedded gene co‐expression network analysis (MEGENA), and Mendelian randomization (MR) to uncover key genes causally linked to epilepsy risk. By integrating bioinformatics with experimental validations, this study provides valuable insights into the biological mechanisms of epilepsy and the development of novel therapeutic strategies.

## 2 Methods

### 2.1 Data Extraction, Differential Gene Expression, and Enrichment Analysis

Data for this study were extracted from the Series Matrix File of GSE143272, available in the GEO database (https://www.ncbi.nlm.nih.gov/geo/). The annotation file used was GPL10558, encompassing expression profile data from 34 patients with epilepsy and 50 healthy subjects as controls. Analysis of the normalized data was performed using the limma package in R (version 4.2.2) to identify differential expression genes (DEGs) between epilepsy blood samples and healthy controls, *p* < 0.05 and |logFC| > 0.5. Volcano plots and heatmaps were generated using the ggplot2 package in R with default parameters (https://cran.r‐project.org/package=ggplot2).

For enrichment analysis of the DEGs, we utilized the clusterProfiler package from Bioconductor, focusing on Gene Ontology (GO) and Kyoto Encyclopedia of Genes and Genomes (KEGG) pathway analyses. A *p* value and *q* value < 0.05 were considered statistically significant, indicating enrichment in the identified pathways.

### 2.2 MEGENA

The co‐expression network was constructed using MEGENA based on standardized gene expression data [14, 15]. The process involved filtering gene pairs with significant correlations (FDR < 0.05), followed by ranking effective gene pairs according to Pearson correlation coefficients (PCCs). The planar maximally filtered graph algorithm was employed to conduct planarity tests on the significantly ranked PCCs, determining whether gene pairs could be mapped on a three‐dimensional topological sphere without intersecting other edges [16]. This iterative testing of planarity resulted in the generation of a Planar Filtered Network (PFN). Subsequent multiscale clustering analysis (MCA) of the PFN identified gene co‐expression modules, or network clusters, at various resolutions of compactness. MCA partitioned the principal module into multiple sub‐modules by searching for partitions optimized for Newman's modularity (*Q*) [17].

### 2.3 Mendelian Randomization Analysis

The MR Base database (http://app.mrbase.org/) serves as the primary source for acquiring summary statistics data from genome‐wide association studies (GWAS). This study aimed to identify key genetic factors associated with epilepsy. Specifically, the epilepsy dataset (ebi‐a‐GCST90018840) included 4382 cases and 453,928 controls. DEGs between disease and control samples were identified and utilized as the target gene group. Expression quantitative trait loci (eQTL) data were obtained from the eQTLGen consortium database (https://www.eqtlgen.org), which endeavors to study the genetic architecture of blood gene expression and understand the genetic basis of complex traits.

The “ieugwasr” R package was employed to extract genetic variants associated with eQTLs, termed instrumental variables (IVs), related to the target genes. Selected potential genetic IVs typically represent single nucleotide polymorphisms (SNPs) associated with each gene at a genome‐wide significance threshold (*p* < 1e‐5), relevant to the expression or function of the target genes. Linkage disequilibrium between SNPs was calculated to ensure that selected variations were statistically significant and independent. Only SNPs with an *r* ^2^ < 0.001 (clumping window size = 10,000 kb) and p2 < 5e‐8 were retained (where *r* ^2^ is the clumping *r* ^2^ cut‐off, defaulting at 0.001, and p2 is the secondary clumping threshold, defaulting at 5e‐8). Subsequently, the “extract_outcome_data” function in the “TwoSampleMR” R package was utilized to obtain result data associated with specific SNPs and GWAS study IDs. The “harmonise_data” function integrated the IVs and outcome data. To ensure the accuracy and reliability of the MR analysis, the data were formatted, directionally inconsistent SNPs were calibrated, and mismatched or missing genetic variants were excluded.

The reliability of causal relationships was assessed based on the number of SNPs, providing an overall estimate of the impact of both cis and some trans gene expressions in whole blood samples on epilepsy. For causal relationships involving only one SNP, the Wald ratio method was utilized for MR analysis. In cases with multiple SNPs, various methods were applied: the inverse variance weighted method, combining Wald estimates of each SNP through meta‐analysis; MR Egger regression, assuming instrument strength independent of direct effect, to evaluate unreasonable heterogeneity or bias in IVs; Weighted median, allowing accurate estimation of causal relationships even when up to 50% of IVs are ineffective; Weighted mode, estimating causality with increased detection power, less bias, and lower type I error rates compared to MR‐Egger regression. Finally, causal relationships identified in the MR analysis underwent validation analysis. The “mr_heterogeneity” function performed heterogeneity testing (Cochran's Q test) to detect significant differences in estimated causal effects among different SNPs used in the MR analysis. Pleiotropy tests were conducted to assess whether SNPs directly affect the outcome (epilepsy occurrence), thus avoiding bias in MR estimates. Leave‐one‐out analysis aided in identifying specific SNPs significantly influencing overall MR analysis results. All statistical tests were two‐sided, with *p* < 0.05 considered statistically significant.

### 2.4 Disease Gene Correlation and miRNA‐mRNA Regulatory Network

Genes related to epilepsy were downloaded from the GeneCards database (https://www.genecards.org/). The top 20 genes with the highest relevance scores were selected for correlation analysis with the expression levels of key genes obtained from MR analysis. Pearson correlation analysis and heatmap generation were conducted using R. Positive correlations. The miRNAs are small non‐coding RNAs that have been shown to regulate gene expression by promoting mRNA degradation or inhibiting mRNA translation. We further analyzed whether certain miRNAs regulate the transcription or degradation of risk genes in key genes. miRNAs related to key genes were obtained from the miRcode database (http://www.mircode.org) and visualized the miRNA network of genes using Cytoscape software 3.6.1.

### 2.5 Immune Infiltration Analysis

Immune infiltration analysis offers critical insights into the exploration and prediction of the immune microenvironment in samples from epilepsy patients. Leveraging the CIBERSORT algorithm, which operates on the principle of support vector regression, enables deconvolution analysis of immune cell subtype expression matrices. This method distinguishes among 22 types of immune cells—including B cells, T cells, monocytes, and macrophages—using 547 included biomarkers. We evaluated the relative proportions of immune cells in blood samples obtained from GSE143272, providing insight into the extent of infiltration by various immune cell types in these samples (with the permutation test number, ‘perm’, set to 100). Pearson correlation analysis and Wilcoxon rank‐sum tests were conducted to assess the relationship between gene expression levels and immune cell content.

### 2.6 Gene Set Variation Analysis (GSVA) and Gene Set Enrichment Analysis (GSEA)

GSVA represents a non‐parametric, unsupervised approach for gauging the enrichment of gene sets in transcriptomic data. It translates variations at the gene level into changes at the pathway level, facilitating the evaluation of potential alterations in biological functions across samples. In this study, gene sets were sourced from the Molecular Signatures Database (MSigDB), and expression data underwent filtering to eliminate genes with a high proportion of zero values. The GSVA algorithm was then employed to assign enrichment scores to each gene set.

GSEA excels in identifying subtle or moderate specific gene expressions within a given microarray dataset, thereby scrutinizing differences in signaling pathways between high and low expression groups. Version 7.0 annotated gene sets from the MSigDB were utilized as subtype pathway annotated gene sets. The “limma” R package facilitated linear model fitting of the matrix and application of the eBayes function, enhancing the identification of DEGs and generating the requisite gene ranking list for GSEA. Subsequently, GSEA was performed using the “clusterProfiler” R package, aiming to elucidate key biological pathways associated with the expression of genes of interest, while also assessing trends in changes in gene set enrichment scores. An adjusted p‐value of < 0.05 was considered statistically significant.

### 2.7 Construction of the Epilepsy Animal Model

We established a temporal lobe epilepsy (TLE) animal model by stereotactically injecting kainic acid (KA) into the hippocampus. TLE is one of the most common types of epilepsy. The experimental setup involved male C57BL/6 mice, aged 8 weeks, weighing 24–26 g [18]. Each mouse underwent weighing, anesthesia, and secure fixation in a stereotaxic frame. The skull was exposed to locate the hippocampus (CA1) based on coordinates (AP: −2.0, ML: 1.5, DV: −2.0) from the mouse brain atlas. Precision drilling at these coordinates was conducted using a 0.5 mm skull drill. KA (0.5 mg/mL, Cayman Chemicals) was intrahippocampally administered using a Hamilton syringe attached to a Quintessential Stereotaxic Injector (Stoelting), injecting 100 nL over 1 min. The control group received phosphate‐buffered saline (PBS). The needle remained in place for an additional 2 min post‐injection to prevent backflow [19]. The incision was sealed with Vetbond dermal glue (3 M), and the mouse was placed in a warmed recovery chamber. Seizures were monitored and scored using a modified Racine scale for 2 h post‐injection. Intrahippocampal KA injection induces convulsive status epilepticus; thus, seizures were behaviourally quantified according to Racine's criteria, excluding EEG analysis [20]. After injection, animals were housed under a 12‐h light–dark cycle (light: 8:00–20:00) in a controlled environment at 22°C ± 1°C and 50%–60% humidity, with free access to food and water.

The latent period, spanning from the initial cerebral insult to the development of chronic epilepsy, ranged from 2 to 14 days in this model [21]. Therefore, mice were sacrificed 2 weeks later (all having experienced spontaneous recurrent seizures within 1 week prior to sacrifice), with rapid extraction of whole brains and hippocampi. Whole brains were paraffin‐embedded, while hippocampi were flash‐frozen in liquid nitrogen. Subsequently, all samples were stored at −80°C for future experiments. All procedures were approved by the Animal Ethics Committee of Capital Medical University.

### 2.8 RNA Isolation and Quantitative Polymerase Chain Reaction (qPCR)

Total RNA was isolated from mouse hippocampus using chloroform and the RNeasy Mini Kit (Qiagen). cDNA was synthesized using the SweScript All‐in‐One SuperMix for qPCR. Subsequently, qPCR was performed using the Universal Blue SYBR Green qPCR Master Mix on the QuantStudio 5 Real‐Time PCR System. Gene expression levels were normalized against GAPDH. Each sample was tested in triplicate, and their Ct values were averaged. Relative expression levels were calculated using the 2^−ΔΔCT^ method. Primer sequences are provided in the Supporting Information.

### 2.9 Western Blot (WB)

Hippocampal samples from mice were lysed using buffer containing 100 mM NaCl, 50 mM Tris–HCl, 0.5% NP‐40, and a 1× concentration of both protease and phosphatase inhibitors (Thermo Fisher, 78,440). Immunoprecipitation was performed overnight at 4°C using IgG or antigen‐specific antibodies attached to Dynabeads Protein G (Thermo Fisher, 10004D). For standard WB analysis and capillary electrophoresis, cellular lysates were prepared using Laemmli buffer (Bio‐Rad) supplemented with the same inhibitor cocktail (Thermo Fisher, 78,440). Electrophoresis was conducted on a 4%–12% SDS‐PAGE gel (Invitrogen, NP0321BOX), followed by protein transfer to PVDF membranes. These membranes were then blocked using 5% BSA in TBST (0.1% Tween 20) before incubation with specific antibodies. WB images were captured using an Azure (C300) imaging system (Azure Biosystems). Capillary electrophoresis was performed using the Jess system (JS4929), following the protocols provided by ProteinSimple, San Jose, CA (SM‐W004).

For standard WB, the primary antibodies used were CDC25B (#ab124819, 1:1000, Abcam), MTX1 (#NBP1‐87741, 1:5000, Novus), RAF1 (#ab137435, 1:1000, Abcam), SSH2 (#H00085464‐D01P, 1:500, Novus), GZMA (1288‐1‐AP, 1:1000, Proteintech), SH3BP5L (#NBP1‐81381, 1:5000, Novus), FGD3 (#NBP3‐12395, 1:5000, Novus), DNMT1 (#ab13537, 1:1000, Abcam), and ACTIN (GB11001, 1:1000, Servicebio). The secondary antibodies used were Anti‐Rabbit IgG (H + L) Secondary Antibody, HRP (#31460; 1:3000, Thermo Fisher), and Anti‐Mouse IgG (H + L) Secondary Antibody, HRP (#13–4813‐85; 1:3000, Thermo Fisher). For protein capillary electrophoresis, the primary antibodies used were IQGAP1 (#ab133490, 1:50, Abcam), BKRB2 (#YN2508; 1:50, Immunoway), PAK2 (#2608S, 1:50, CST), ARPC1B (#28368‐1‐AP, 1:50, Proteintech), and RAC2 (#10735‐1‐AP; 1:50, Proteintech). The secondary antibodies used followed the manufacturer's instructions: Anti‐Rabbit Secondary HRP Antibody (#042–206, 1:50, ProteinSimple), and Anti‐Mouse Secondary HRP Antibody (#042–205, 1:50, ProteinSimple).

### 2.10 Immunofluorescence Analysis

All immunofluorescence procedures were carried out with mild agitation at room temperature. Tissue slices underwent three washes in PBS for 10 min each, followed by overnight incubation with primary antibodies. These antibodies were diluted in a solution of PBS with 0.3% Triton X100 and 5% normal serum (either goat or donkey, depending on the secondary antibody species), except when employing the Mouse‐on‐Mouse Fluorescein kit. Following primary antibody incubation, slices were subjected to another three PBS washes, followed by a 2‐h dark incubation with the secondary antibody solution prepared in PBS with 0.3% Triton X100, except when using the Mouse‐on‐Mouse kit. Subsequently, slices were briefly rinsed in PBS for 5 min and stained with the DAPI nuclear marker (# D1306, 1:10000, Molecular Probes) for 5 min. After a final PBS rinse, slices were mounted on gel‐coated slides, cover‐slipped with Prolong gold anti‐fade (# P36930, Vector Laboratories), and stored at 4°C until further analysis. Antibodies were used as follows: CDC25B (#ab124819, 1:50, Abcam), MTX1 (#NBP1‐87741, 1:500, Novus), RAF1 (#ab137435, 1:100, Abcam), SSH2 (k109475p, 1:100, Solarbio), GZMA (11288‐1‐AP, 1:100, Proteintech), SH3BP5L (#NBP1‐81381, 1:1000, Novus), FGD3 (#sc‐390,256, 1:100, Scbt), DNMT1 (#24206‐1‐AP, 1:100, Proteintech).

### 2.11 Tissue Dissociation and Cell Purification

Hippocampal tissue was placed in a sterile dish containing 10 mL of 1x DPBS (#14190144, Thermo Fisher) on ice to remove any remaining storage solution, followed by mincing. Tissue digestion was performed using 0.25% Trypsin (#25200–072, Thermo Fisher) and 10 μg/mL DNase I (#11284932001, Sigma) in PBS with 5% FBS. The dissociation occurred at 37°C with a shaking speed of 50 rpm for approximately 40 min, with intermittent harvesting of cells every 20 min to enhance yield and viability. The cell suspension was then filtered through a 40‐μm nylon filter, and red blood cells were lysed using 1X Red Blood Cell Lysis Solution (#00–4333‐57, Thermo Fisher). After washing with DPBS (2% FBS), cell viability was assessed using 0.4% Trypan blue on a Countess II Automated Cell Counter.

### 2.12 10× Library Preparation and Sequencing

For 10x library preparation, cells and beads carrying unique molecular identifiers (UMIs) and cell barcodes were combined to near saturation in Gel Beads‐in‐emulsion (GEMs). Cell lysis released polyadenylated RNAs, which hybridized to beads. These beads underwent reverse transcription, tagging each cDNA at its 5′ end with UMIs and cell labels. The process included second‐strand cDNA synthesis, adaptor ligation, and amplification. Sequencing libraries were generated focusing on 3′ transcript ends, following the Chromium Single Cell 3′ v3.1 protocol. The libraries were quantified using a High Sensitivity DNA Chip (Agilent) on a Bioanalyzer 2100 and the Qubit High Sensitivity DNA Assay (Thermo Fisher Scientific). Sequencing was performed on Xplus (Illumina) using 2×150 chemistry.

### 2.13 Single‐Cell RNA Sequencing (scRNA‐Seq) Data Processing

Using Cell Ranger (v7.1.0), reads underwent processing, alignment to the mouse GRCm38 genome via STAR, and UMI counting to generate Gene‐Barcode matrices, with non‐cell barcodes filtered out. Post‐integration into Seurat (v4.1.1) for quality control, cells were filtered based on transcript counts, gene counts, and mitochondrial gene reads percentage, within predefined thresholds. Data normalization and variable gene identification were performed, followed by data integration across samples using ‘anchors’. Principal Component Analysis (PCA) was then applied, focusing on the top 30 components, followed by Uniform Manifold Approximation and Projection (UMAP) for 2D cluster visualization. Cell clustering was executed via graph‐based clustering on PCA‐reduced data using the Louvain Method, following the construction of a shared nearest neighbor graph. Sub‐clustering utilized the same scaling, dimensionality reduction, and clustering approach (UMAP) on specific data subsets, often confined to one cell type. Significant DEGs in each cluster compared to others were identified using the Wilcoxon Rank‐Sum Test. Cell types were determined using SingleR and known marker genes [22].

### 2.14 Machine Learning

We employed seven algorithms—logistic regression (LR), decision tree (DT), random forest (RF), multilayer perceptron (MLP), support vector machine (SVM), light gradient boosting machine (LightGBM), and K‐nearest neighbor (KNN)—to build models using fivefold cross‐validation on the training cohort.

### 2.15 SHAP Analysis

SHAP (SHapley Additive exPlanations), a game‐theoretic method for explaining the output of any machine learning model, was conducted using the fastshap R package (v 0.1.1).

### 2.16 Model Evaluation

Accuracy, balanced accuracy, Cohen's Kappa, F1‐score, negative predictive value (NPV), positive predictive value (PPV), precision‐recall area under curve (PR‐AUC), precision, recall, receiver operating characteristic area under curve (ROC‐AUC), sensitivity, specificity, and Youden index were calculated for model evaluation.

### 2.17 Statistical Analysis

For normally distributed data, the independent samples t‐test compared means of two independent sample groups. For non‐normally distributed data or small sample sizes not meeting normality assumptions, the non‐parametric Mann–Whitney *U* test was used. Significance was set at *p* < 0.05, and all values are expressed as mean ± SD. Data analysis and graphing utilized R Language (version 4.2.2) and GraphPad Prism 8 software (GraphPad Software Inc.).

## 3 Results

### 3.1 DEGs And Pathway Enrichment in Epilepsy

**FIGURE 1:**
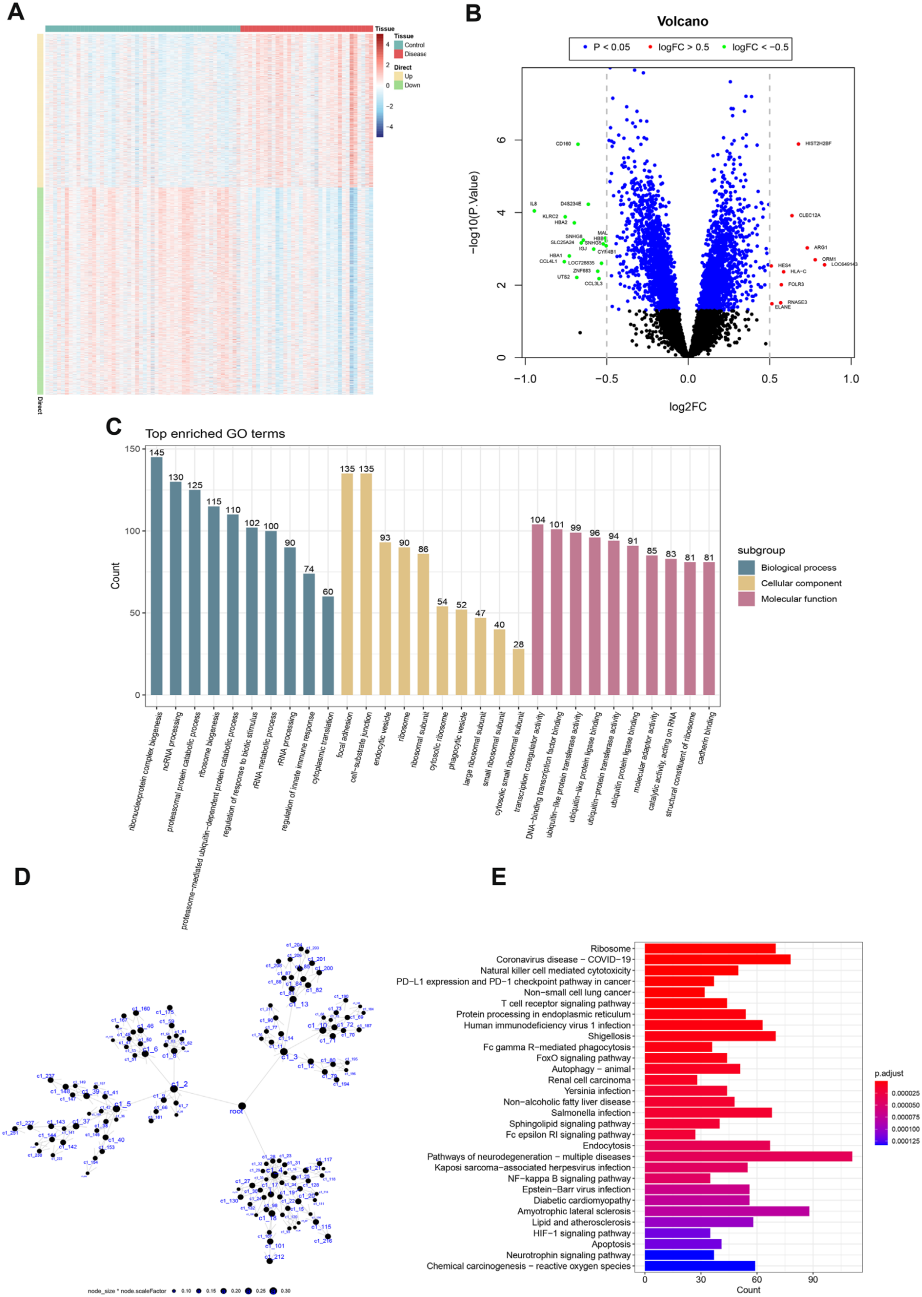
Transcriptomic insights into differential gene expression and pathway enrichment. (A, B) Analysis of two groups with limma identifies 3619 DEGs (*p* < 0.05), including 1541 upregulated and 2078 downregulated genes. The heatmap displays expression patterns (A), while the volcano plot indicates DEG distribution (B). (C) GO enrichment analyses highlights the top 10 enrichments of DEGs in the categories of biological process, cellular component, and molecular function. (D) Co‐expression network analysis through multiscale embedded gene co‐expression network analysis highlights significant gene pairs (FDR < 0.05) from the 3619 DEGs, ranking them by correlation coefficients for clustering. The network diagram emphasizes genes with ‘module.hub’ and ‘child.module’ node shapes in category ‘c’ for further candidate gene selection. (E) Kyoto Encyclopedia of Genes and Genomes pathway analysis presents the top 30 core pathways enriched with DEGs, such as T cell receptor signaling pathway, FoxO signaling pathway, and sphingolipid signaling pathway. (DEGs, differential expression genes; GO, Gene Ontology).

In GSE143272, comprising 84 participants, 50 belonged to the normal group, and 34 to the disease group. A total of 3619 DEGs were identified, consisting of 1541 upregulated genes and 2078 downregulated genes (Figure 1A,B). Biological processes enriched in ribonucleoprotein complex biogenesis and ncRNA processing, cellular components notably observed in cytoplasmic translation and focal adhesion, while molecular functions predominantly enriched in transcription coregulator activity and DNA‐binding transcription factor binding (Figure 1C). The 3619 DEGs formed the candidate gene set for MEGENA module clustering analysis. A detailed module network map was generated for the module with the largest node size value (Figure 1D), retaining genes in the network nodes marked as ‘module. hub’ and ‘child. module’ class c for subsequent analysis. KEGG analysis revealed significant enrichment of DEGs in pathways such as neurodegeneration, T cell receptor signaling, and natural killer cell‐mediated cytotoxicity (Figure 1E). Ultimately, 248 genes were selected for the candidate gene set. The raw data and code are provided in Appendix S1.

### 3.2 Identification of Significant Causal Relationships Between Gene Expression and Epilepsy Risk

**FIGURE 2:**
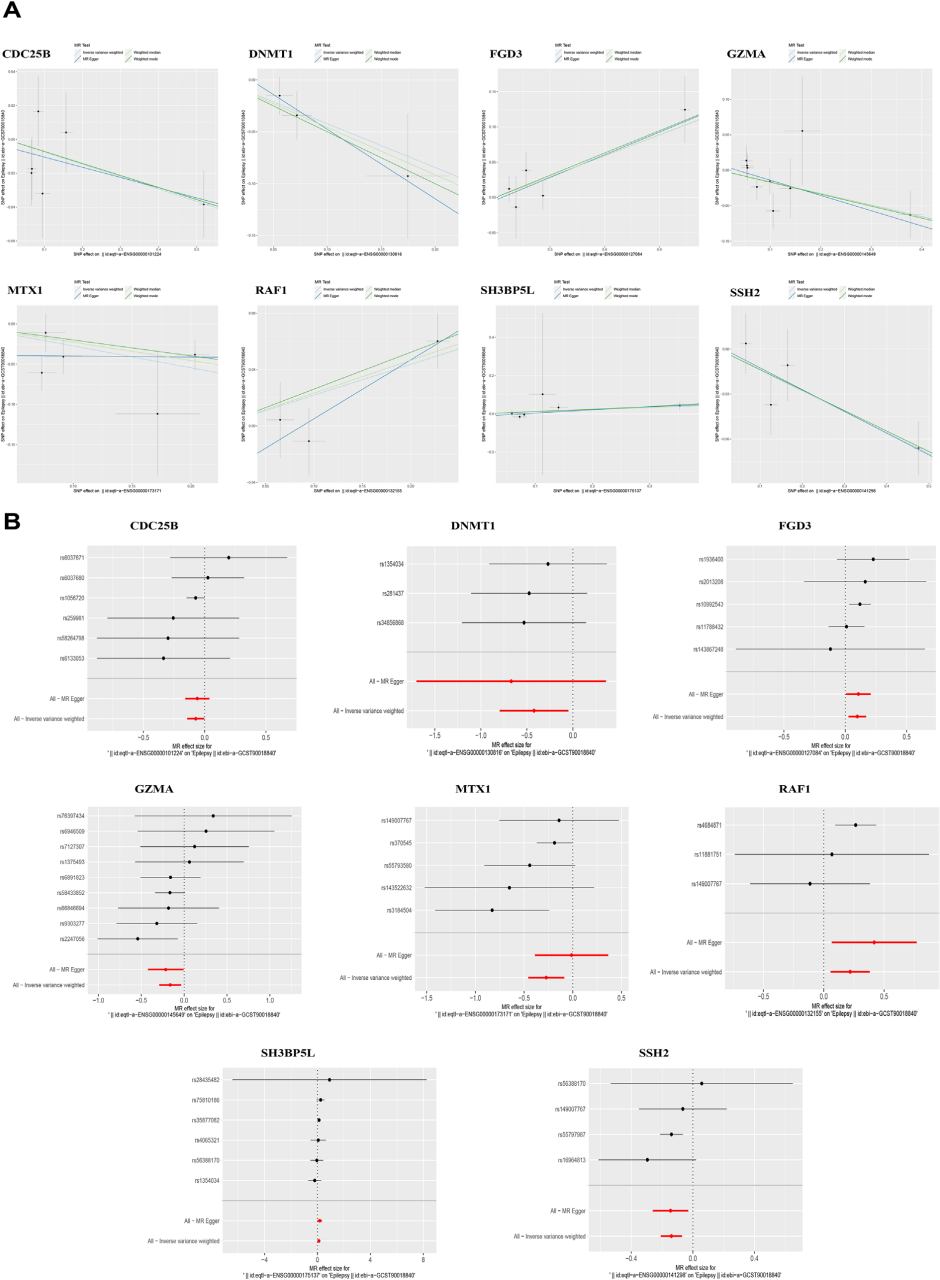
Summary of Mendelian randomization analysis results on epilepsy. (A) The collection of scatter plots depicting the MR analysis of the causal relationship between the expression of 8 genes (*CDC25B*, *DNMT1*, *FGD3*, *GZMA*, *MTX1*, *RAF1*, *SH3BP5L*, *SSH2*) and the positive eQTL outcome for epilepsy (*p* < 0.05). Each graph is dedicated to a single gene (name written above each graph), where every point signifies an individual SNP selected as an instrumental variable, detailing its impact on gene expression (*x*‐axis) and its corresponding influence on epilepsy risk (*y*‐axis). The analysis employs four MR methods—Inverse Variance Weighted, Weighted Median, MR Egger, and Weighted Mode—to test the relationship, with slopes visualizing the effect size of each method. Error bars on each data point depict the 95% CI for the effect size estimations. (B) Forest plots illustrate association between 8 genes (see A for definitions) and epilepsy using individual or combined SNPs with MR‐Egger's or inverse variance‐weighted methods. Bars depict effect size and 95% CI. (MR, Mendelian randomization; CI, confidence intervals)

To identify key genes, we analyzed summary statistics from 458,310 samples (453,928 controls and 4382 cases) linked to epilepsy, resulting in the outcome ID: ebi‐a‐GCST90018840. From the initial 248 candidate genes, causal relationships with the outcome were extracted for 143 genes using the ‘extract_instruments’ and ‘extract_outcome_data’ functions. MR analysis further revealed causal relationships for eight key genes corresponding to eQTL‐positive outcomes (*p* < 0.05): *CDC25B* (OR = 0.93; 95%CI = 0.87–0.99; *p* = 0.04), *DNMT1* (0.66; 0.45–0.95; *p* = 0.03), *GZMA* (0.85; 0.75–0.97; *p* = 0.01), *MTX1* (0.76; 0.63–0.92; *p* < 0.01), and *SSH2* (0.87; 0.81–0.93; *p* < 0.01) may be associated with a lower risk of epilepsy, while *FGD3* (1.19; 1.10–1.03; *p* < 0.01), *RAF1* (1.24; 1.06–1.46; *p* < 0.01), and *SH3BP5L* (1.12; 1.00–1.24; *p* = 0.04) with a higher risk (Figure 2A). No heterogeneity was identified in the causal relationships for the eight key genes (*p* > 0.05, Figure 2B). Sensitivity analysis using Leave‐One‐Out demonstrated that removing any one SNP did not significantly impact the overall error line, suggesting robustness in the selected causal relationships. Relevant data are provided in Appendix S1.

### 3.3 Disease Gene Expression Level and miRNA‐mRNA Regulatory Network

The heatmap illustrates the correlation matrix for 20 disease‐regulating genes and eight key genes (Figure S1). The expression levels of eight key genes were significantly correlated with the expression levels of disease regulatory genes. Among them, *DNMT1* and *TBC1D24* were significantly negatively correlated (*r* = −0.695), while *MTX1* and *TBC1D24* were significantly positively correlated (*r* = 0.82). We screened *CDC25B*, *DNMT1*, *FGD3*, *GZMA*, *MTX1*, *RAF1*, *SH3BP5L*, *SSH2* using the miRcode database and performed reverse prediction on these genes. A total of 84 miRNAs and 292 mRNA‐miRNA interaction pairs were identified (Figure S1).

### 3.4 Key Genes and Their Connection to the Peripheral Immune Environment in Epilepsy

**FIGURE 3:**
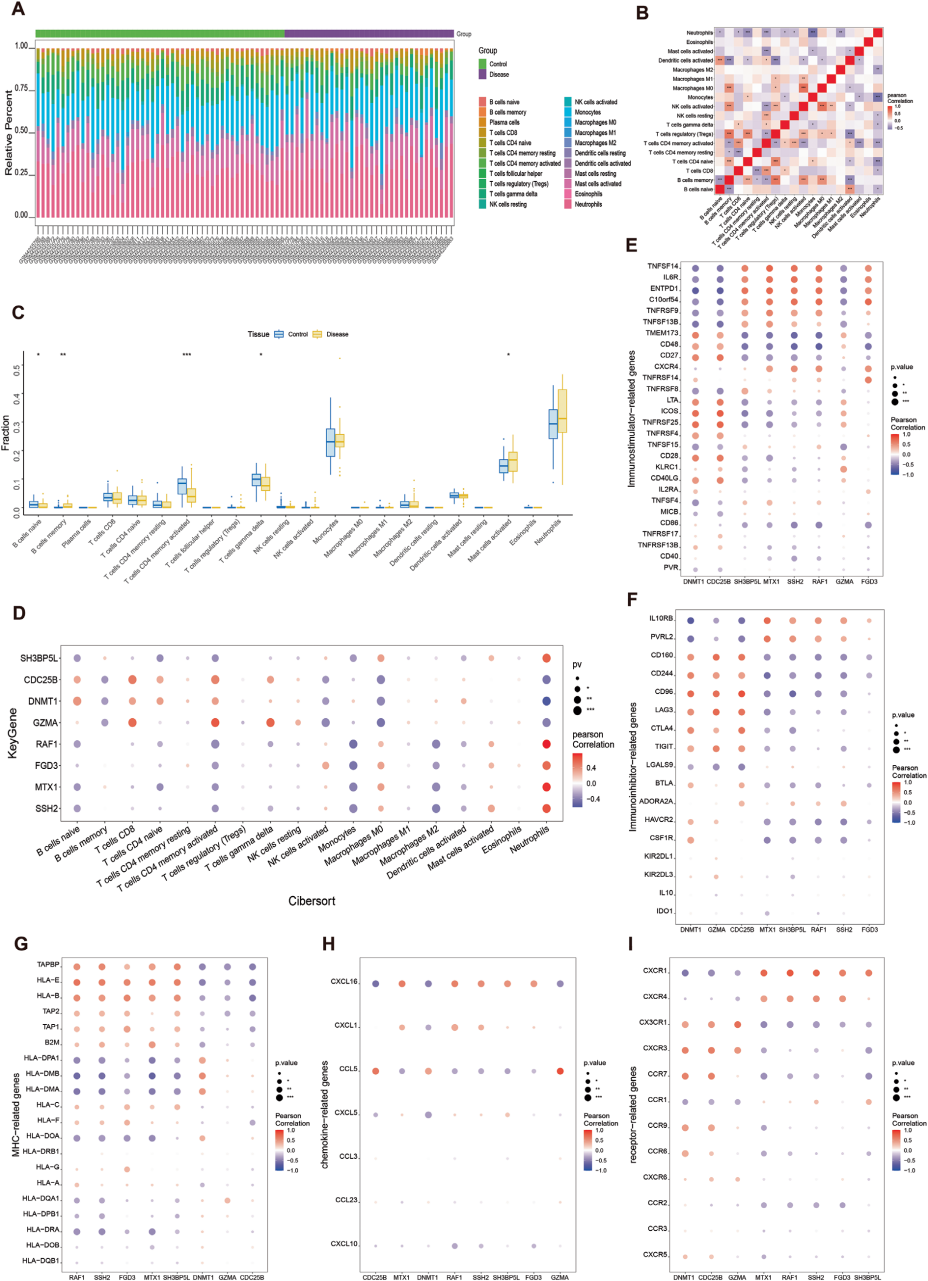
Immune cell profiling and key gene‐immune microenvironment associations in epilepsy. (A) The comparison of infiltrating immune cells between epilepsy patients and health control. (B) Heatmap illustrating the correlations among immune cells, where color depth signifies the strength of association, and shades of red indicate positive while shades of blue represent negative correlations. (C) Box plot representation of differential immune cell presence between control and disease groups, highlighting disparities in populations such as naive B cells, memory B cells, activated CD4 memory T cells, gamma delta T cells, and activated mast cells, with statistical significance denoted (Wilcoxon rank‐sum test, *p < 0.05; **p < 0.01; ***p < 0.001; ns, not significantly different). (D–I) Correlations between the expression of eight key genes and various immune modulators, including (D) immune cells, (E) immuno‐stimulator related genes, (F) immuno‐inhibitor related genes, (G) MHC related genes, (H) chemokine related genes, and (I) immune receptor related genes. The color scale reflects the magnitude of Pearson correlation coefficient (red: Positive, blue: Negative) and the dot size indicates the *p* value (*p < 0.05; **p < 0.01; ***p < 0.001; ns, not significantly different).

The immune microenvironment, comprising immune cells, fibroblasts, extracellular matrix, growth factors, inflammatory factors, and specific physicochemical characteristics, significantly impacts disease diagnosis, survival outcomes, and clinical treatment sensitivity. In Figure 3A, we observe the infiltration levels of various immune cells, while Figure 3B illustrates the correlations among these immune cells. Notably, in patients with epilepsy, the peripheral immune environment exhibits a marked increase in activated mast cells and memory B cells, accompanied by significant decreases in CD4 memory activated T cells and γδ T cells (Figure 3C).

To delve deeper, we explored the relationship between eight key genes and immune cells, uncovering strong correlations between several genes and various immune cell types (Figure 3D). These correlations were derived from the TISIDB database, encompassing immunostimulatory factors, immunoinhibitory factors, MHC‐related genes, chemokines, and immune‐related receptors. As depicted in Figure 3E–I, our results indicate robust correlations between key genes and a diverse array of immune factors. These findings underscore the intimate connection of these key genes with peripheral immune cell infiltration and suggest their significant role in shaping the peripheral immune microenvironment.

### 3.5 GSVA And GSEA Identify Key Pathways of Eight Genes in Epilepsy

Subsequently, we investigated the specific signaling pathways enriched by the key genes to explore the potential molecular mechanisms influencing the progression of epilepsy. GSVA revealed high expression of *CDC25B*, enriched in the unfolded protein response and fatty acid metabolism pathways; *DNMT1*, enriched in the P53 pathway and DNA repair; *FGD3*, enriched in cholesterol homeostasis and apoptosis; *GZMA*, enriched in E2F targets and IL2/STAT5 signaling; *MTX1* and *RAF1*, enriched in the reactive oxygen species pathway, with *MTX1* also enriched in PI3K/AKT/mTOR signaling and *RAF1* in haem metabolism; *SH3BP5L*, enriched in bile acid and xenobiotic metabolism; and *SSH2*, enriched in Notch and Wnt/β‐Catenin signaling (Figure S2A).

GSEA analysis indicated that *CDC25B* is enriched in pathways of primary immunodeficiency, ribosome, and T cell receptor signaling pathway; *DNMT1* in the cell cycle, pyruvate metabolism, and relaxin signaling pathway; *FGD3* in the GnRH signaling pathway, necroptosis, and sphingolipid metabolism; *GZMA* in allograft rejection, chemokine signaling pathway, and Th17 cell differentiation; *MTX1* in the cAMP signaling pathway, endocytosis, and TNF signaling pathway; *RAF1* in the estrogen signaling pathway, Fc epsilon RI signaling pathway, and insulin signaling pathway; *SH3BP5L* in the JAK–STAT signaling pathway, MAPK signaling pathway, and prolactin signaling pathway; *SSH2* in the adipocytokine signaling pathway, GnRH signaling pathway, and IL‐17 signaling pathway (Figure S2B).

### 3.6 Key genes' mRNA and Proteins Elevated in the Hippocampus of Epileptic Mice

**FIGURE 4:**
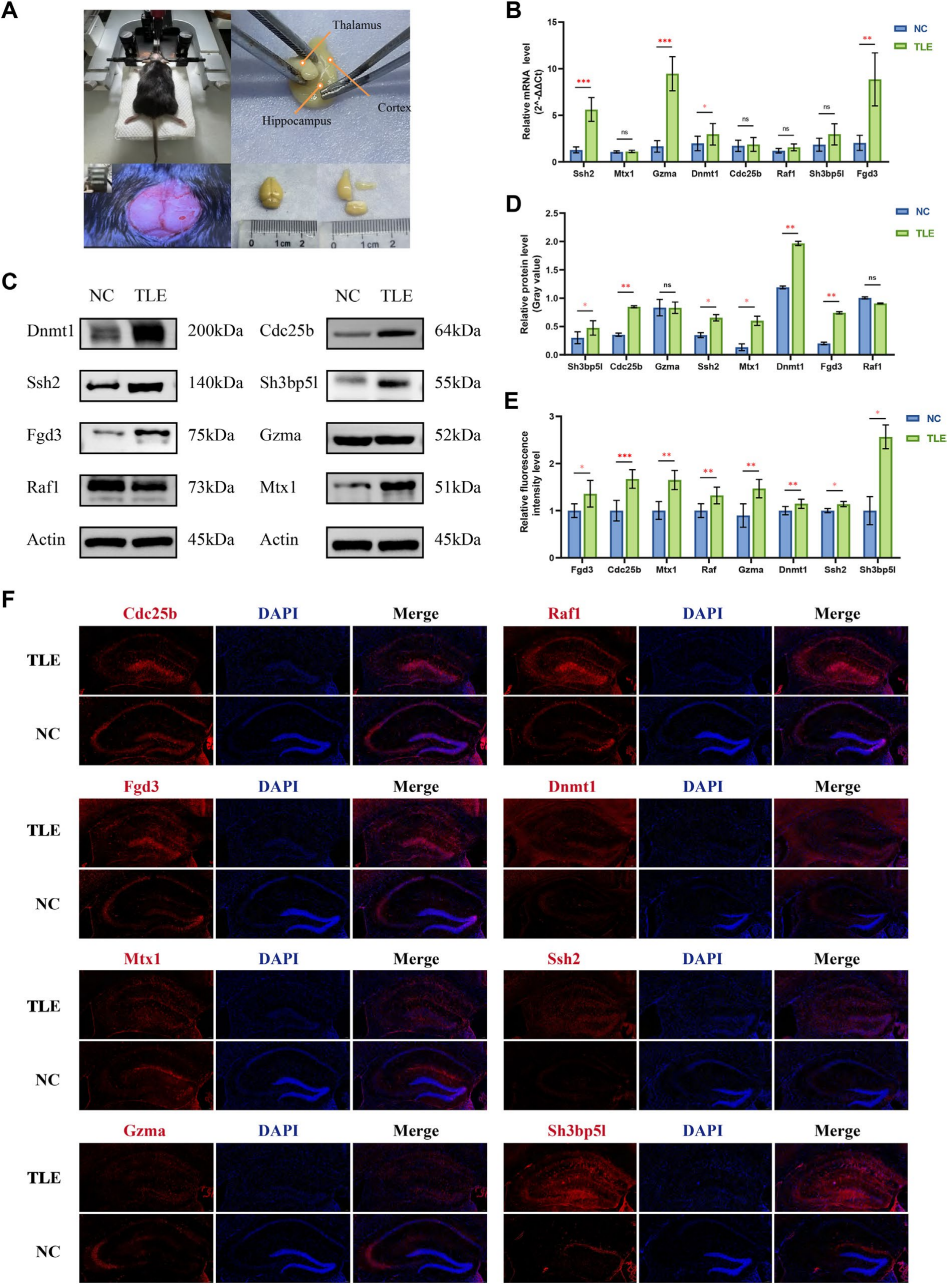
Expression of key genes at mRNA and protein levels in the hippocampus of TLE animal model. (A) Construction of the TLE mouse model. It primarily illustrates the process of animal anesthesia and fixation, kainic acid injection localization, brain tissue separation on ice, and hippocampus extraction. (B) Expression levels of core genes' corresponding mRNA in the hippocampus of NC and TLE groups identified by qPCR. Data are shown as mean ± SE. (C) Western blot analysis of hippocampal lysates displaying the protein levels of Dnmt1, Cdc25b, Ssh2, Fgd3, Gzma, Raf1, and Mx1 with Actin serving as a loading control. Approximate molecular weights are indicated. (D) Quantification of Western blot analysis of the protein bands in Figure C, through relative gray values compared across NC and TLE hippocampus samples. (E) Quantitative analyses of immunofluorescence staining showing the fluorescence intensity between NC and TLE in Figure F. (F) Representative immunofluorescence images showing the hippocampal localization of Cdc25b, Raf1, Fgd3, Dnmt1, Mx1, and Ssh2 (red) with DAPI staining the nuclei (blue) in NC and TLE groups (Scale bar, 100 μm). (TLE, temporal lobe epilepsy; NC, normal control. *p < 0.05, **p < 0.01, ***p < 0.001, ns, not significant.)

To validate the mRNA and protein expression of key genes in vivo, we established a TLE mouse model (Figure 4A). qPCR analysis was performed on hippocampal samples from 12 mice in the TLE group, treated with KA, and 12 from the control group, treated with PBS. The mRNA levels of *Ssh2*, *Gzma*, *Dnmt1*, and *Fgd3* were significantly higher in the hippocampus of the TLE group compared to the control (Figure 4B). For both WB and immunofluorescence experiments, each group consisted of six mice. The expression of Sh3bp5l, Cdc25b, Ssh2, Mtx1, Dnmt1, and Fgd3 was notably elevated in the hippocampus of the TLE group compared to the control (Figure 4C,D). Immunofluorescence analysis revealed a significant upregulation of all related proteins in the hippocampus of the TLE group compared to controls (Figure 4E,F). Overall, *Fgd3*, *Ssh2*, and *Dnmt1* showed significantly elevated mRNA and protein expressions in the TLE hippocampus. Appendix S1 provides the original data.

### 3.7 scRNA‐Seq for Mouse Hippocampus of TLE Model

Single‐cell analysis was conducted on cell suspensions derived from hippocampal tissue. The scRNA‐seq data were categorized into a TLE group and a blank control group, with four hippocampal samples each from mouse models treated with KA and PBS, respectively. Of the initial cell count, 13,121 cells in the control group and 11,433 cells in the TLE group met quality control standards, with a base error rate of less than 0.02%. The correlation coefficient between nCount_RNA, representing the number of unique molecular identifiers (UMIs), and nFeature_RNA, representing the number of genes, was 0.93. Additionally, there was a negative correlation coefficient of −0.23 between nCount_RNA and percent.mito, indicating good quality of the filtered cells. Utilizing the top 2000 highly variable genes from each sample, we selected information from the first 30 dimensions for dataset representation and conducted dimensionality reduction analysis. Further details are provided in Appendix S2.

**FIGURE 5:**
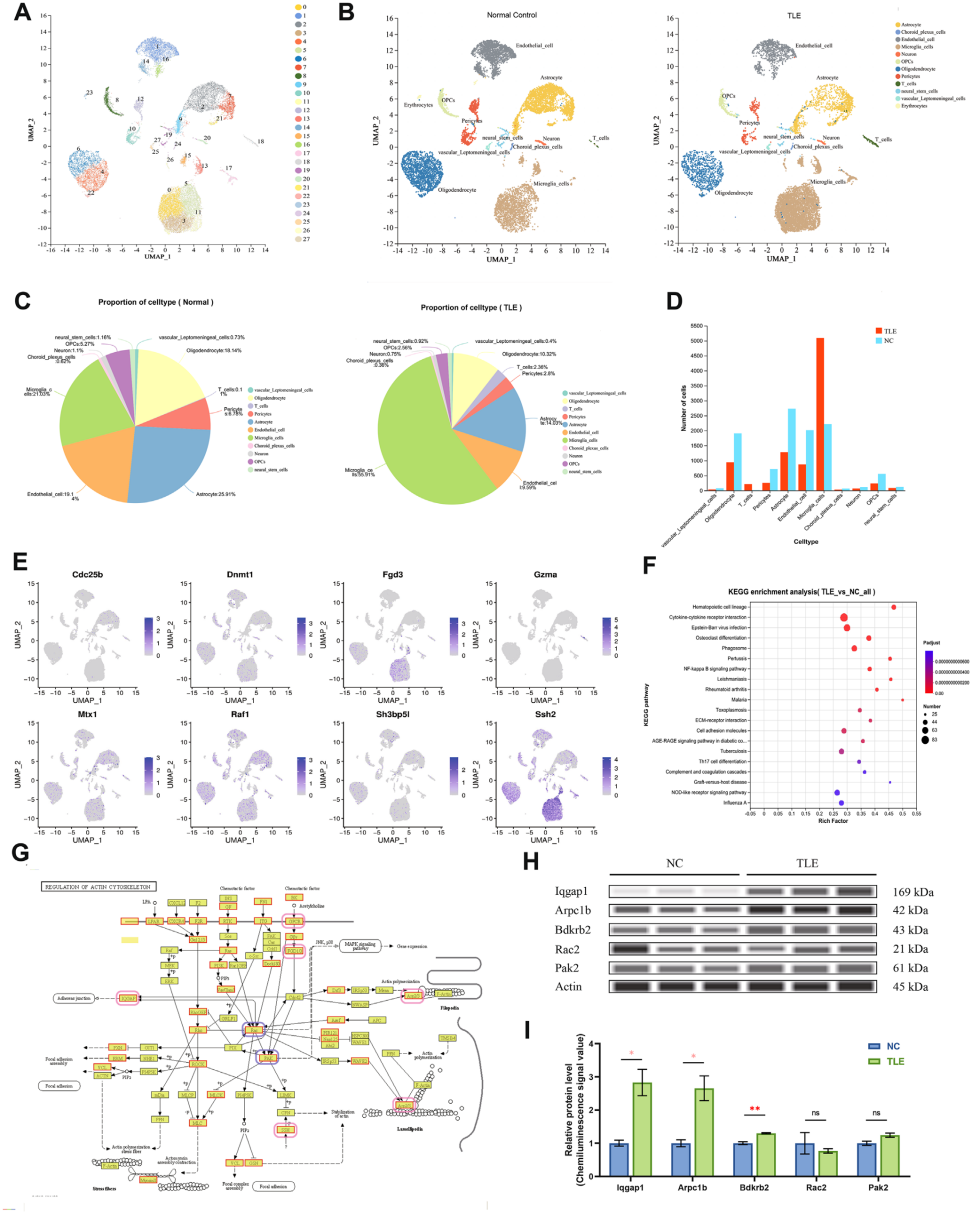
Single‐cell transcriptomic analysis reveals cellular heterogeneity in the NC and TLE group. (A) UMAP visualization of the single‐cell RNA‐seq data from normal and TLE hippocampus samples, displaying diverse cell populations. The horizontal and vertical axes represent components after dimensionality reduction. Each point in the figure represents a cell, with cells in close proximity considered to be of the same type. Different cell groups are distinguished by different colors. (B) UMAP plots highlighting the distribution of 12 identified cell types in NC (four samples, left) and TLE groups (four samples, right). The horizontal and vertical axes represent components after dimensionality reduction. Each point in the figure represents a cell, with cells in close proximity considered to be of the same type. Different cell groups are distinguished by different colors. (C) Pie charts showing the proportion of different cell types in the NC (left) and TLE (right) groups, illustrating the changes in cellular composition. (D) Bar graph depicting the count or numbers of each cell type in NC and TLE groups. (E) Visualization of the expression of eight key genes (*Cdc25b*, *Dnmt1*, *Fgd3*, *Gzma*, *Mtx1*, *Raf1*, *Sh3bp5l*, *and Ssh2*) across various cell types in the hippocampal tissue via a UMAP plot of gene expression. It is observed that, except for Fgd3 and Ssh2 which are specifically highly expressed in microglia, and Gzma in a small population of T cells, other genes do not exhibit cell type‐specific expression and are generally distributed across cell types. (F) Bubble chart of pathways significantly enriched in DEGs from single cell RNA sequence analysis of microglia comparing TLE and NC groups. Top 20 key pathways are listed on the *y*‐axis, with the rich factor (*x*‐axis) indicating enrichment strength. Bubble size symbolize the count of genes involved, while color intensity reflects the significance of enrichment, with darker red indicating higher statistical significance. (G) KEGG pathway enrichment analysis while comparing microglia in TLE versus NC, for the target gene set (*Fgd3* and *Ssh2*). This KEGG pathway enrichment analysis delineates the regulation of the actin cytoskeleton, with a specific emphasis on the roles of GPCRs (*Bdkrb2*), FGD1/3 (*Fgd3*), IQGAP (*Iqgap1*), Rac (*Rac2*), PAK (*Pak2*), SSH (*Ssh2*), and the Arp2/3 (*Arpc1b*) complex. Arrow (→) indicates a promoting or activating effect. T‐shaped line (⊣) indicates an inhibitory or blocking effect. All entities with colored backgrounds in the diagram are part of the KEGG annotation results for genes/transcripts under TLE versus NC comparison. Yellow background indicates known genes/transcripts, while green background represents new genes/transcripts (none). Red borders denote upregulated genes, and blue borders indicate downregulated genes (none). Genes encircled in pink and blue ellipses are those for which protein expression validation was conducted subsequently (Figure E). Pink signifies that the corresponding gene's protein level in the TLE hippocampus is significantly upregulated, consistent with predictions, whereas blue indicates no significant upregulation was found. (H) Capillary‐based immunoblots showing the expression of key proteins upstream and downstream of Fgd3 and Ssh in the regulation of the actin cytoskeleton pathway. (I) Quantification of Western blot analysis of the protein bands, using relative chemiluminescence signal values compared across NC and TLE hippocampus samples. (*p < 0.05, **p < 0.01, ***p < 0.001, ns, not significant). (TLE, temporal lobe epilepsy; NC, normal control.)

After removing batch effects and performing clustering analysis based on transcriptome similarity, cells were divided into 28 major clusters (Figure 5A), with their top 10 marker genes, proportion, and inter‐cluster correlations presented in Appendix S2. Following cell annotation using SingleR and manual curation, 12 cell types were identified: microglia, astrocytes, oligodendrocytes, choroid plexus cells, endothelial cells, neurons, oligodendrocyte precursor cells, pericytes, neural stem cells, T cells, vascular leptomeningeal cells, and erythrocytes (Figure 5B). In the control group, astrocytes were the most prevalent (25.9%), while microglia dominated the TLE group (55.9%) (Figure 5C). Generally, microglia were the most numerous cell type (7306 cells), followed by astrocytes (4006 cells) and oligodendrocytes (2850 cells) (Figure 5D). In the TLE group, apart from an increase in microglia and T cells, other cell types were reduced compared to controls (Figure 5D). Since our single‐cell analysis was based on suspension preparation to ensure the capture of information from as many different cell types as possible, the number of neuronal cells was relatively low.

The standardized expression of eight key genes is visualized on a UMAP plot (Figure 5E). In AUCell scoring, the gene set comprising eight key genes exhibited the highest scores in microglia (Appendix S2), indicating a central role of microglia and associated immune functions in epilepsy development. Notably, *Ssh2* and *Fgd3* are predominantly expressed in microglial cells, while the distribution of other genes lacks specificity. DEGs obtained from scRNA‐seq also identified *Ssh2* and *Fgd3*. Furthermore, significantly enriched pathways from scRNA‐seq DEGs (Figure 5F, Appendix S2) were displayed. *Ssh2* and *Fgd3* were notably upregulated and concentrated in the regulation of actin cytoskeleton, among the significant pathways identified in scRNA‐seq DEGs (Figure 5G). Among the upstream and downstream proteins that have a direct promoting effect with Ssh2 and Fgd3, Iqgap1, Arpc1b, and Bdkrb2 were significantly upregulated (Figure 5H,I).

### 3.8 Re‐Clustering Analysis of Microglia and Machine Learning

**FIGURE 6:**
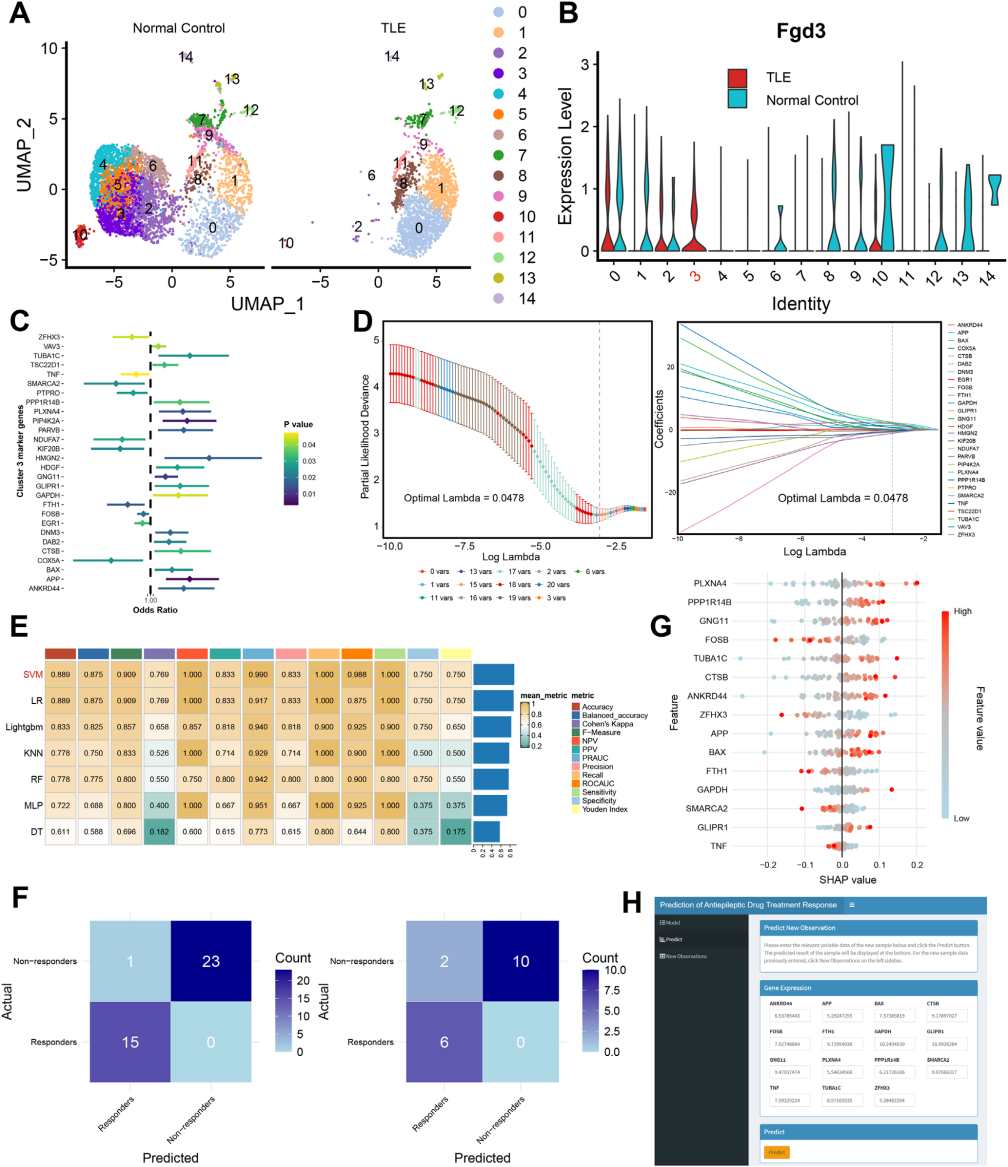
Immune cell profiling and key gene‐immune microenvironment associations in epilepsy. (A) UMAP plot of re‐clustered microglia categorized by tissue type, identifying a total of 15 microglial sub‐groups. (B) Expression of FGD3 across the 15 microglial sub‐groups, showing the most prominent expression in Cluster 3, which is predominantly associated with the disease (TLE) group. (C) Analysis of marker genes in Cluster 3 microglia within the GSE143272 cohort using univariate logistic regression identified 28 genes related to drug treatment response. (D) LASSO regression analysis for identifying key genes predictive of antiepileptic drug response, including optimal lambda selection (0.0478) based on partial likelihood deviance (left) and associated gene coefficients (right). (E) Performance comparison of seven machine learning models on the testing dataset. (F) Confusion matrices for the SVM model on training (left) and testing (right) sets. (G) SHAP analysis of the SVM model, with higher SHAP values indicating greater contributions to drug resistance. (H) The initial prediction model for antiepileptic drug treatment response, which is deployed on an online platform. (TLE, temporal lobe epilepsy; SHAP, SHapley Additive exPlanations; SVM, support vector machine; NR, non‐responders; R, responders).

Since FGD3 is primarily expressed in microglia, we conducted an in‐depth analysis of microglia. By re‐clustering, we categorized microglia into 15 subpopulations (Figure 6A). Further analysis revealed that *Fgd3* is highly expressed in Cluster 3 (Figure 6B). Notably, Cluster 3 microglia were only detected in the TLE group and were entirely absent in normal tissue, suggesting that this cluster represents an epilepsy‐specific microglial subpopulation with disease‐specific high expression of *Fgd3*.

We then aimed to develop a predictive model for antiepileptic drug response in newly diagnosed epilepsy patients based on marker genes of Cluster 3 microglia. In the GSE143272 cohort, univariate logistic regression analysis identified 28 genes associated with drug response (Figure 6C). After further LASSO selection, 15 key genes were identified (Figure 6D). Using these genes, we built seven machine learning models and evaluated their performance on the test set (Figure 6E). The SVM model demonstrated the best performance, with its confusion matrix shown in Figure 6F. SHAP analysis explored each core gene's contribution to the model outcome, indicating that the high expression of certain genes (such as *PLXNA4*, *PPP1R14B*, *GNG11*, *CTSB*, *and BAX*) positively contributed to poor drug response (Figure 6G).

### 3.9 Development of Web Tools to Facilitate Clinical Applications

To facilitate clinical application, we deployed the trained model on an online platform (https://jsxwhosp.shinyapps.io/drugresponseprediction/). Clinicians can use this model for preliminary assessment of antiepileptic drug response in newly diagnosed epilepsy patients, assisting in clinical decision‐making (Figure 6H).

## 4 Discussion

As one of the most prevalent neurological disorders, epilepsy is primarily characterized by abnormal excessive or synchronous neuronal activity in the brain [23]. Upon entering the chronic phase, particularly during the DRE stage, this propensity for seizures is accompanied by a range of neurobiological, cognitive, and psychological consequences. Despite growing interest and attention to genetic and inflammatory factors in epilepsy, systematic studies remain limited [24, 25, 26, 27]. Bioinformatics analyses have provided valuable insights into understanding disease mechanisms [28, 29]. Here, we identified eight genes in peripheral blood with significant causal links to epilepsy risk. Subsequently, we discussed the potential characteristics of both peripheral and central immune environments in epilepsy and analyzed the relationship between these genes and the DRE immune landscape.

In patients with epilepsy, the infiltration of peripheral immune cells into the brain plays a crucial role in the pathophysiology of epilepsy. This process is not only associated with the disruption of the blood–brain barrier but also promotes neuroinflammation and the occurrence and persistence of seizures through various mediators within the neurovascular unit [30]. Mast cells, known for releasing histamine and inflammatory mediators involved in allergic reactions and immune defense, exhibit a significant increase in peripheral circulation in epilepsy according to our results. Upon compromise of the blood–brain barrier, they exacerbate chronic neurodegenerative diseases by enhancing hippocampal neuron synaptic excitotoxicity through histamine release and interaction with microglia [31]. In our study, another noteworthy peripheral immune cell was the T cell, where we observed a reduction in activated memory CD4+ T cells and γδ T cells, reflecting the involvement of adaptive pro‐inflammatory immunity in epilepsy. In the kainic acid model, we identified clusters of cytotoxic T cells with a significant increase in the epilepsy hippocampus, expressing characteristic CD8+ genes. It is believed that a higher infiltration of CD8+ T‐cells in the CA1 region of the hippocampus is strongly positively correlated with increased neuronal loss in that area [32, 33]. This feature has been extensively validated in brain tissues of individuals with mesial temporal lobe epilepsy [31]. Microglia were the only cells, aside from T cells, that significantly increased in the epilepsy hippocampus and were the most numerous. While astrocytes, oligodendrocytes, and others also participated in epilepsy's immune processes, microglia, as the primary innate immune responders, were the focus of our analysis.

Exploring genetic regulation variances between the brain and blood is crucial for identifying genes linked to brain traits and disorders [34]. Apart from *DNMT1*, the roles of the other key genes in epilepsy have remained relatively scarce. DNMT1 has garnered attention for its role in epigenetic modifications in epilepsy [35, 36]. It mediates gene expression by increasing promoter methylation or independently of its catalytic activity, thus contributing to DNA damage repair [37], involvement in adipocyte mitochondrial fission [38], and regulation of neuronal tissue compatibility complex expression [39], aligning with our pathway enrichment findings. Moreover, both overexpression and deficiency of DNMT1 may lead to dysregulation of neural differentiation in epilepsy development [35]. Genetic variations of *CDC25B* can lead to defects in neurogenesis [40] and even result in conditions such as cataracts, dilated cardiomyopathy, and multiple endocrine syndromes [41]. GZMA, primarily secreted by cytotoxic lymphocytes like CD8+ T cells, is a granzyme‐like serine protease that modulates processes of excitotoxicity‐induced neuronal plasticity and death [42]. In our study, GZMA is most closely associated with CD8+ T cells, both in the blood of epilepsy patients and in the hippocampus of epilepsy models. MTX1, essential for mitochondrial transport in human neurons, is an outer membrane protein whose knockdown may affect dynamin‐mediated retrograde transport [43]. RAF1 is a member of the RAF kinase family which activates the MAPK pathway as direct KRAS effectors [44]. Suppressing its expression could prevent microglial overactivation and promote neuronal survival [45]. SH3BP5L is a guanine nucleotide exchange factor for Rab11, with limited reports on its function [46]. As a guanine nucleotide exchange factor, FGD3 influences cell morphology and migration [47]. SSH2 is part of a gene family comprising three members (SSH1, SSH2, and SSH3), all of which have been shown to regulate crucial cellular processes, including invasion, migration, and motility [48]. In our study, both FGD3 and SSH2 were significantly upregulated at the transcriptomic and proteomic levels in epilepsy. This elevation is likely associated with the progression and exacerbation of epilepsy. Currently, there were no studies on FGD3 and SSH2 in the central nervous system. We discovered upregulation of FGD3 and SSH2 in the hippocampus of TLE, exhibiting extensive expression in microglia. This indicates that both are involved in the motility of microglia, but their effects are antagonistic. Beyond actin regulation, they significantly enriched in pathways like cholesterol homeostasis and adipocytokine signaling in TLE. Their downstream ARPC1B, crucial in glioma‐associated macrophages, facilitated glioma cell migration, invasion, epithelial‐mesenchymal transition, and fostered an immunosuppressive microenvironment [49], mirroring our findings. Thus, the BDKRB2‐FGD3‐ARPC1B axis and SSH2 likely respond to modulating microglial chemotaxis, polarization, and other functions. Overall, genes identified from eQTL and GWAS analyses offer directions for linking genetics with disease phenotypes and developing novel therapeutic targets.

The primary limitation of this study is the challenge in obtaining extensive and definitive lesion tissue from epilepsy patients due to ethical concerns, let alone acquiring homogeneous normal human brain tissue for comparison. Therefore, inevitable differences exist between animal models and human epilepsy. Moreover, while the KA mouse model accurately simulates seizures and neuroimmune environments, it primarily addresses mesial TLE (particularly with hippocampal sclerosis). Lastly, in our Mendelian randomization and subsequent experimental validation, only FGD3 and SSH2 showed consistent expression trends across different levels. The changes in other key genes remain complex and require further exploration. Our future endeavors will concentrate on systematically validating the findings of this study and integrating them with other research outcomes to elucidate the pathogenesis and resistance mechanisms of epilepsy.

## 5 Conclusion

By focusing on genetic predisposition, we have identified eight key genes significantly causally linked to epilepsy and discussed their relationship with the immune environment in central nervous system. Specifically, the expression of *FGD3* and *SSH2* was upregulated at both the transcriptomic and proteomic levels, and they were characteristically expressed in microglial cells, potentially associated with actin regulation.

## Author Contributions

Guoguang Zhao, Penghu Wei, and Jianwei Shi designed the study. Jianwei Shi and Jing Xie wrote the manuscript and conducted the analysis. Jing Xie, Yongzhi Shan, and Yanfeng Yang provided statistical guidance. Jianwei Shi, Zuliang Ye, Bin Fu, Quanlei Liu, and Jing Xie conducted the experiments. Ting Tang, Quanlei Liu, and Jinkun Xu provided advice on the discussion. The manuscript was completed under the supervision of Penghu Wei, Yongzhi Shan, and Guoguang Zhao.

## Ethics Statement

This study did not involve human subjects. The animal experiments were approved by the Animal Ethics Committee of Xuanwu Hospital, Capital Medical University.

## Consent

The authors have nothing to report.

## Conflicts of Interest

The authors declare no conflicts of interest.

## Supporting information


Figure S1.

Appendix S1.

Appendix S2.

Data S1.

## Data Availability

The authors confirm that the data supporting the findings of this study are available within the article and its Supporting Information. The Supporting Information mentioned in the article are available on Zenodo (https://zenodo.org/uploads/10826906, DOI: 10.5281/zenodo.10826906). However, the complete raw data for single‐cell and spatial transcriptomics analyses contain crucial information for our ongoing research. Therefore, we intend to upload it to Gene Expression Omnibus or another international open‐access platform by 2026. Until then, we are willing to share these data with any researcher or team upon reasonable request.
