# Supplementary material for: Integrated Mendelian Randomization and Single‐Cell Transcriptomics Analysis Identifies Critical Blood Biomarkers and Potential Mechanisms in Epilepsy
Source: CNS Neurosci Ther. 2025 Jan 3;31(1):e70172. doi: 10.1111/cns.70172 (PMC11702437; doi:10.1111/cns.70172)
Supplement: Supplementary file 1 — Figure S1. [file CNS-31-e70172-s003.zip › Supplementary Figures/Supplementary Figure Legends.docx]

**Supplementary Figure: Regulatory network analysis of identified key genes.**

A. Heatmap of Pearson’s correlation coefficients between 8 key genes and epilepsy-related genes through the GeneCards database. Each dot represents the correlation coefficient between a pair of genes. Blue dots represent negative correlations, red dots represent positive correlations, and white ones indicate no correlation. The size of the dots represents the p-value (pv), where larger dots indicate smaller p-values, signifying higher statistical significance of the correlation. Two enlarged sub-panels highlight specific gene pairs: DNMT1 and TBC1D24 with a negative correlation, MTX1 and TBC1D24 with a positive correlation. The line represents the best fit linear regression.

(p < 0.05).

B. An mRNA-miRNA network diagram was constructed by screening the eight key genes through the miRcode database and performing reverse prediction. A total of 84 miRNAs and 292 mRNA-miRNA pairs were identified.
