# Supplementary figures and images for: Integrated Mendelian Randomization and Single‐Cell Transcriptomics Analysis Identifies Critical Blood Biomarkers and Potential Mechanisms in Epilepsy (part 1 of 2)
Source: CNS Neurosci Ther. 2025 Jan 3;31(1):e70172. doi: 10.1111/cns.70172 (PMC11702437; doi:10.1111/cns.70172)

# A

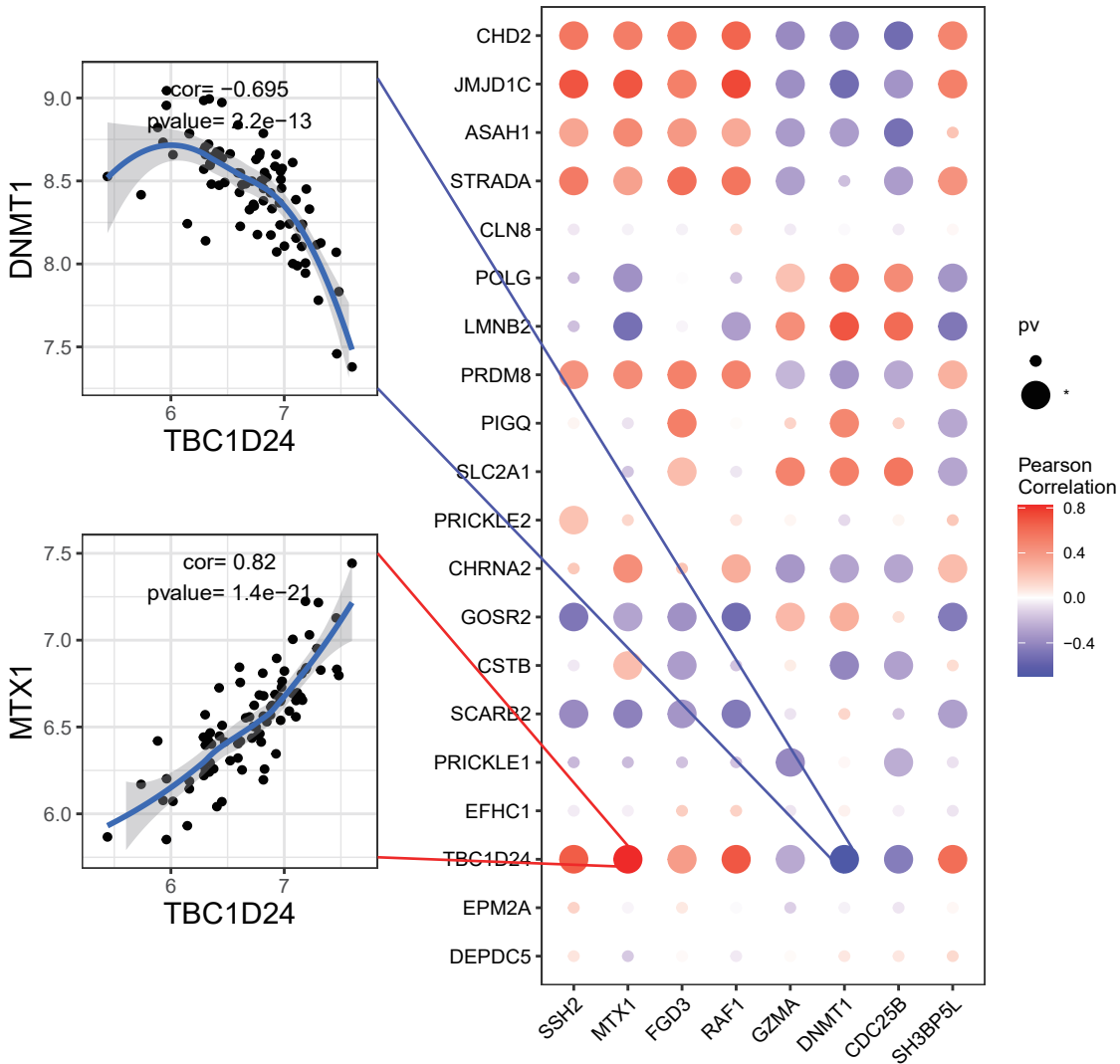

# B

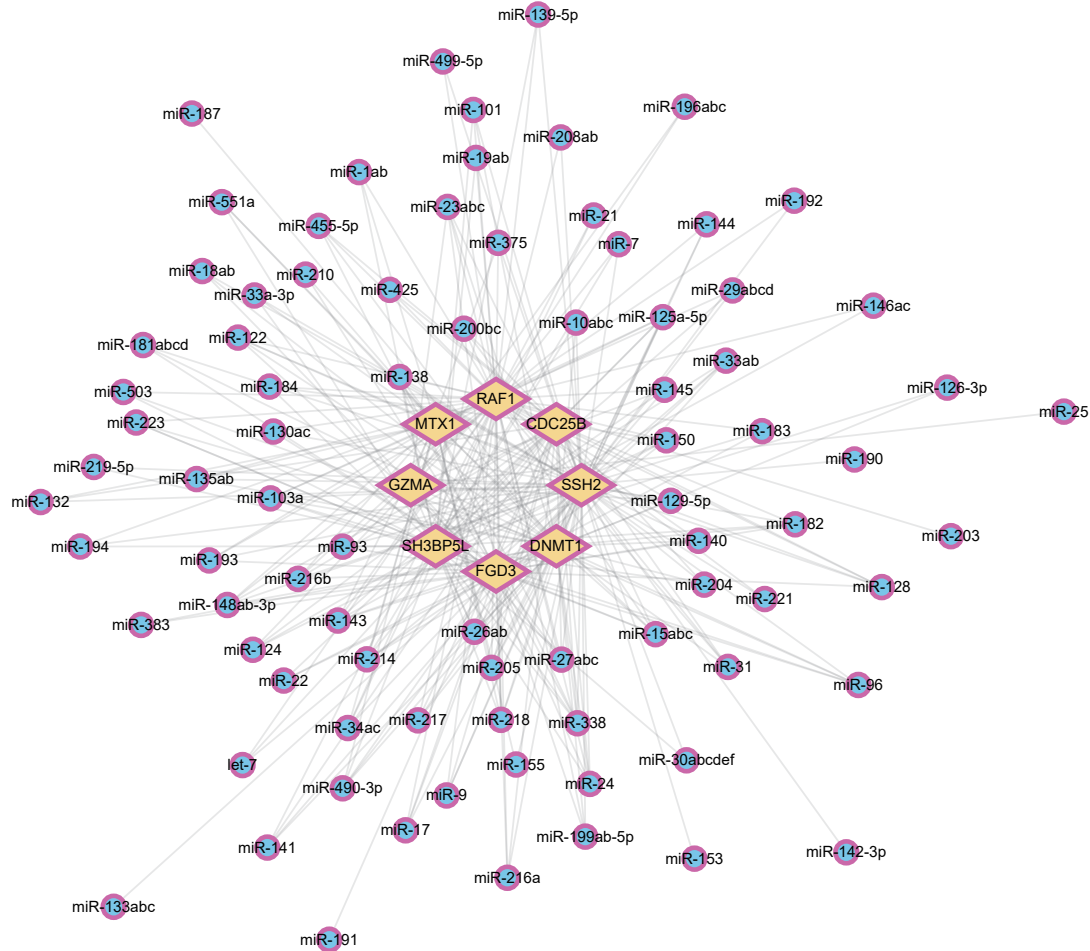

Supplement: Supplementary file 1 — Figure S1. [file CNS-31-e70172-s003.zip › Supplementary Figures/Figure S1.pdf]

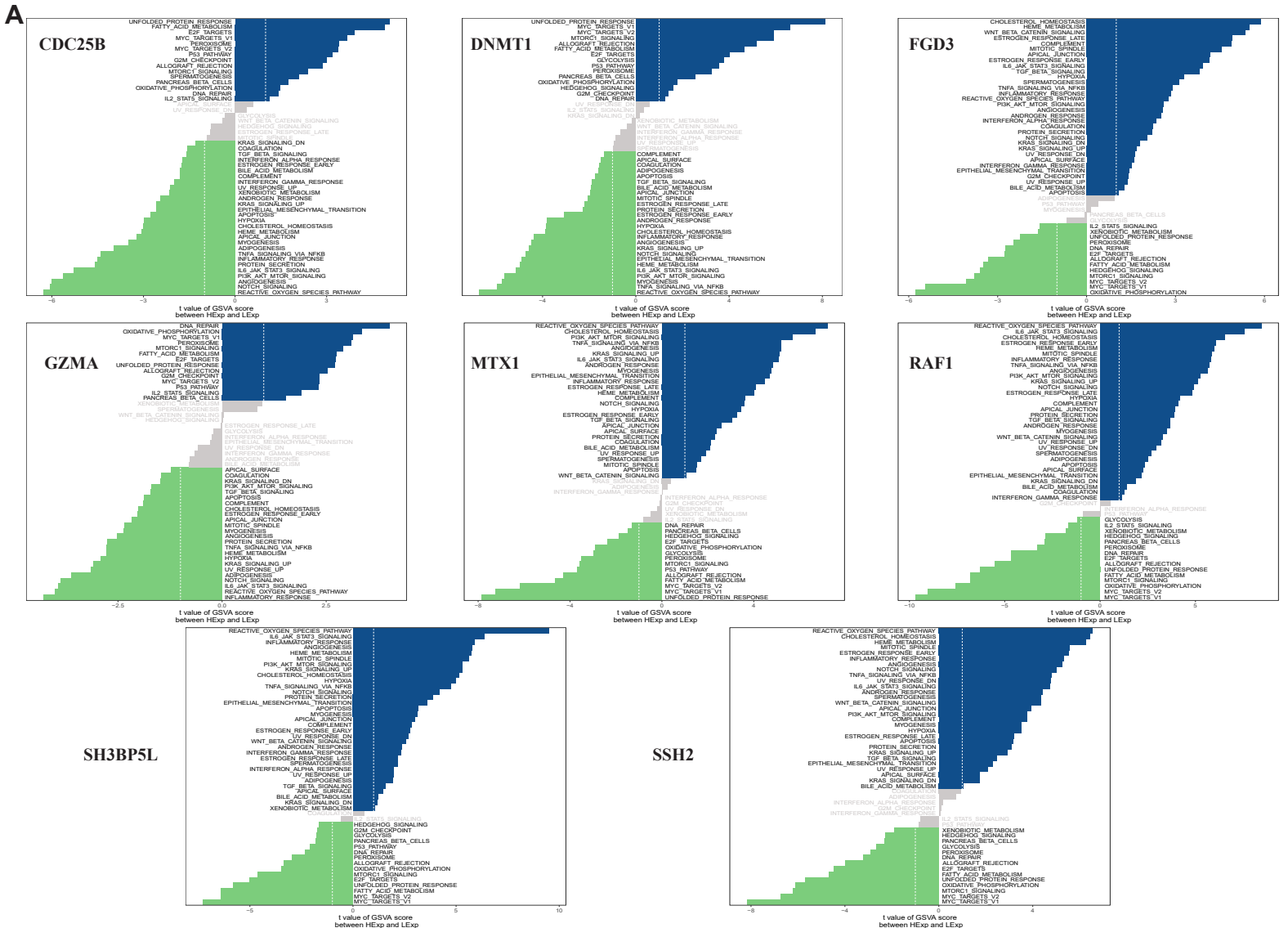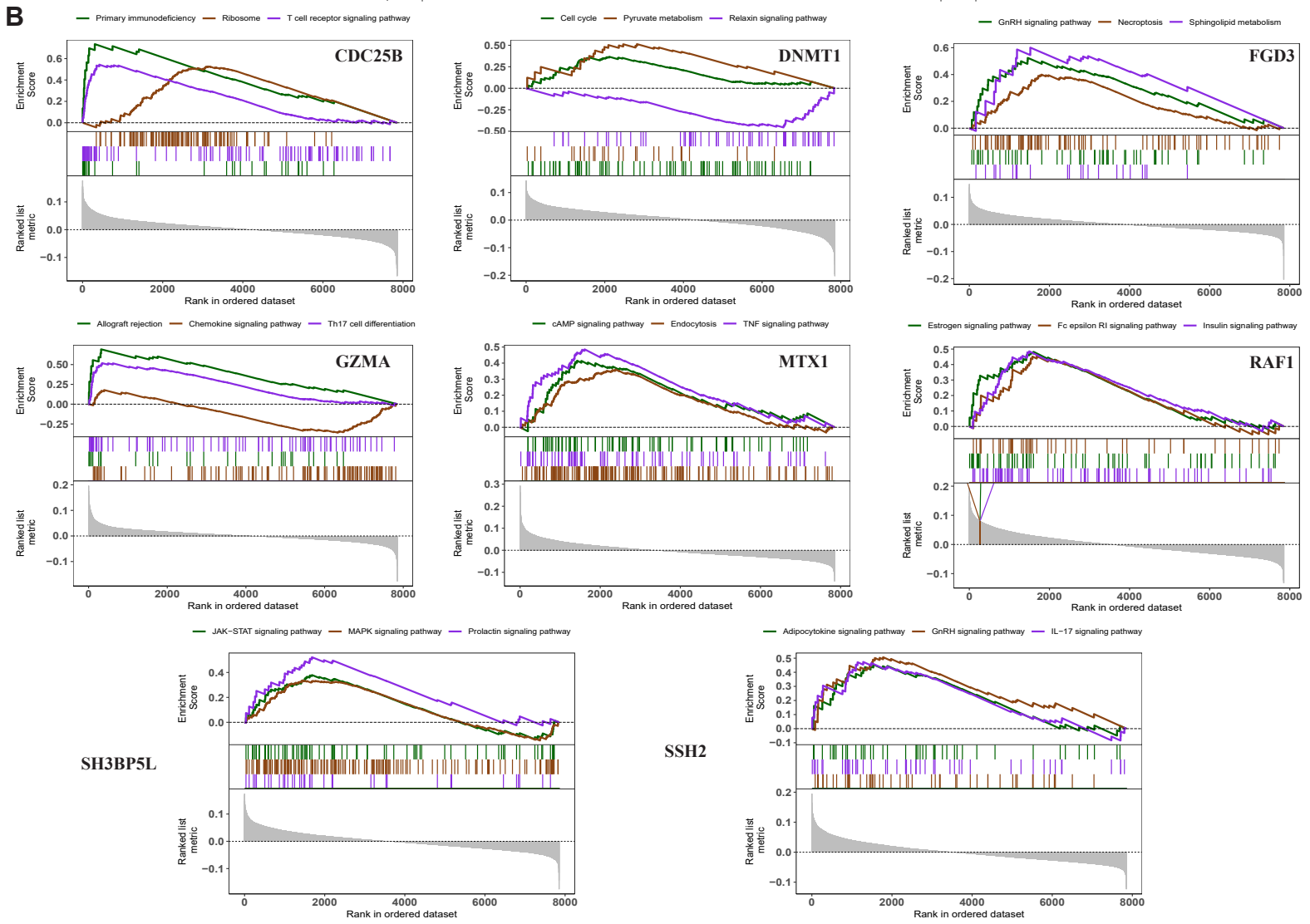

Supplement: Supplementary file 1 — Figure S1. [file CNS-31-e70172-s003.zip › Supplementary Figures/Figure S2.pdf]

# MR Method

- Inverse variance weighted
- MR Egger

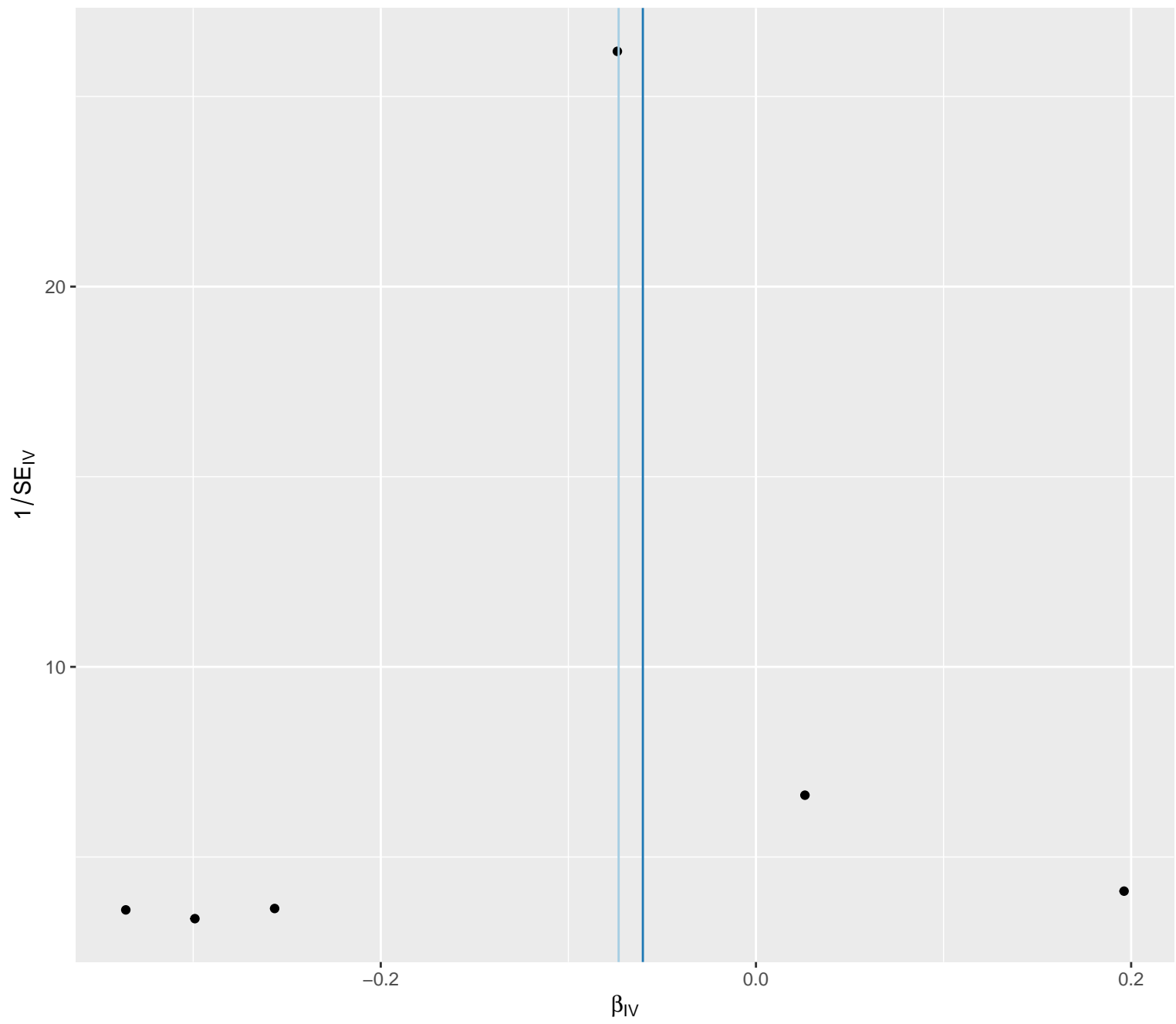

Supplement: Supplementary file 2 — Appendix S1. [file CNS-31-e70172-s001.zip › Supplementary File 1/1_Code and Data for Bio+MR/MF_Heterogeneity analysis/FunnelPlot/CDC25B_H.pdf]

# MR Method

- Inverse variance weighted
- MR Egger

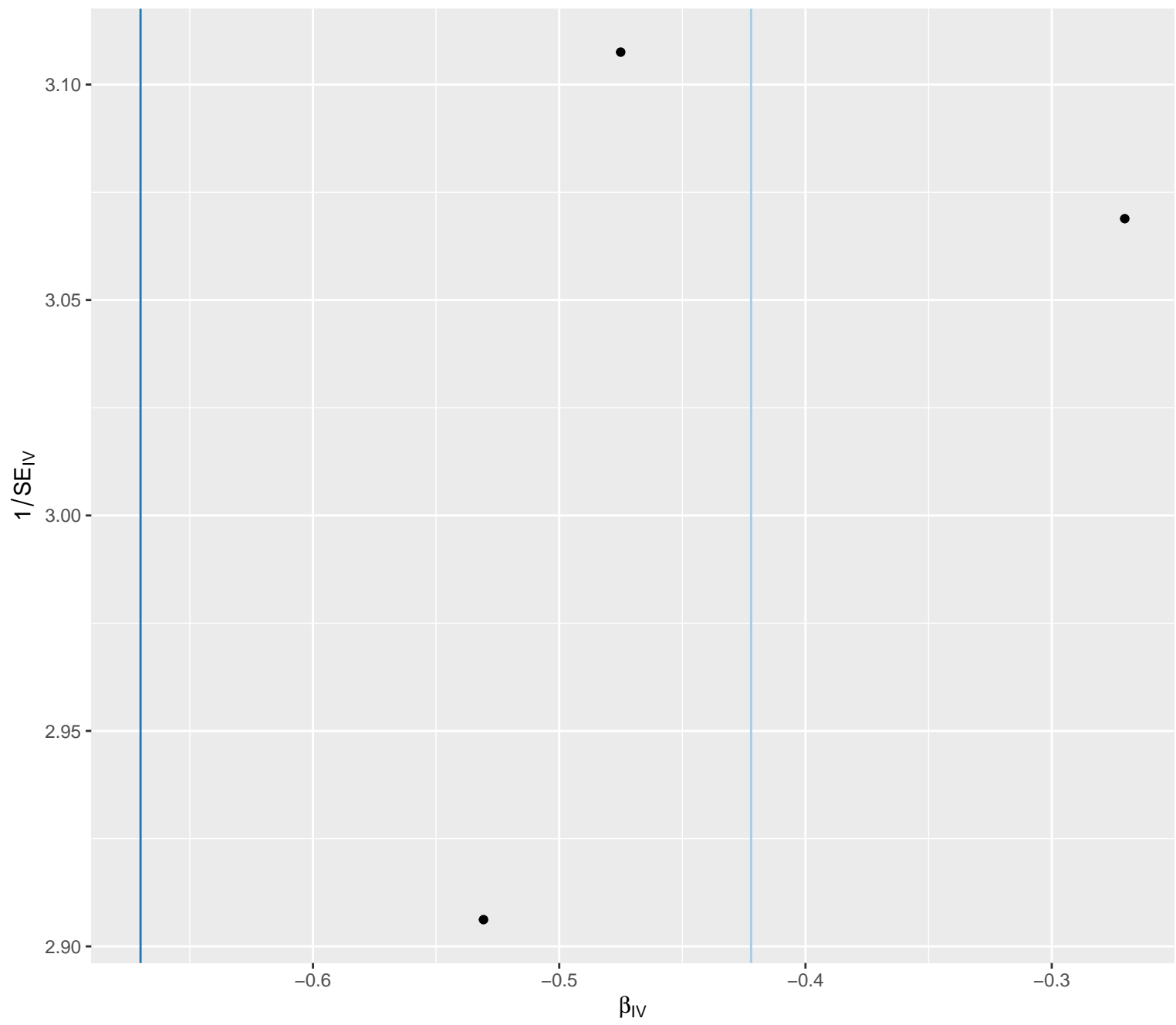

Supplement: Supplementary file 2 — Appendix S1. [file CNS-31-e70172-s001.zip › Supplementary File 1/1_Code and Data for Bio+MR/MF_Heterogeneity analysis/FunnelPlot/DNMT1_H.pdf]

# MR Method

- Inverse variance weighted
- MR Egger

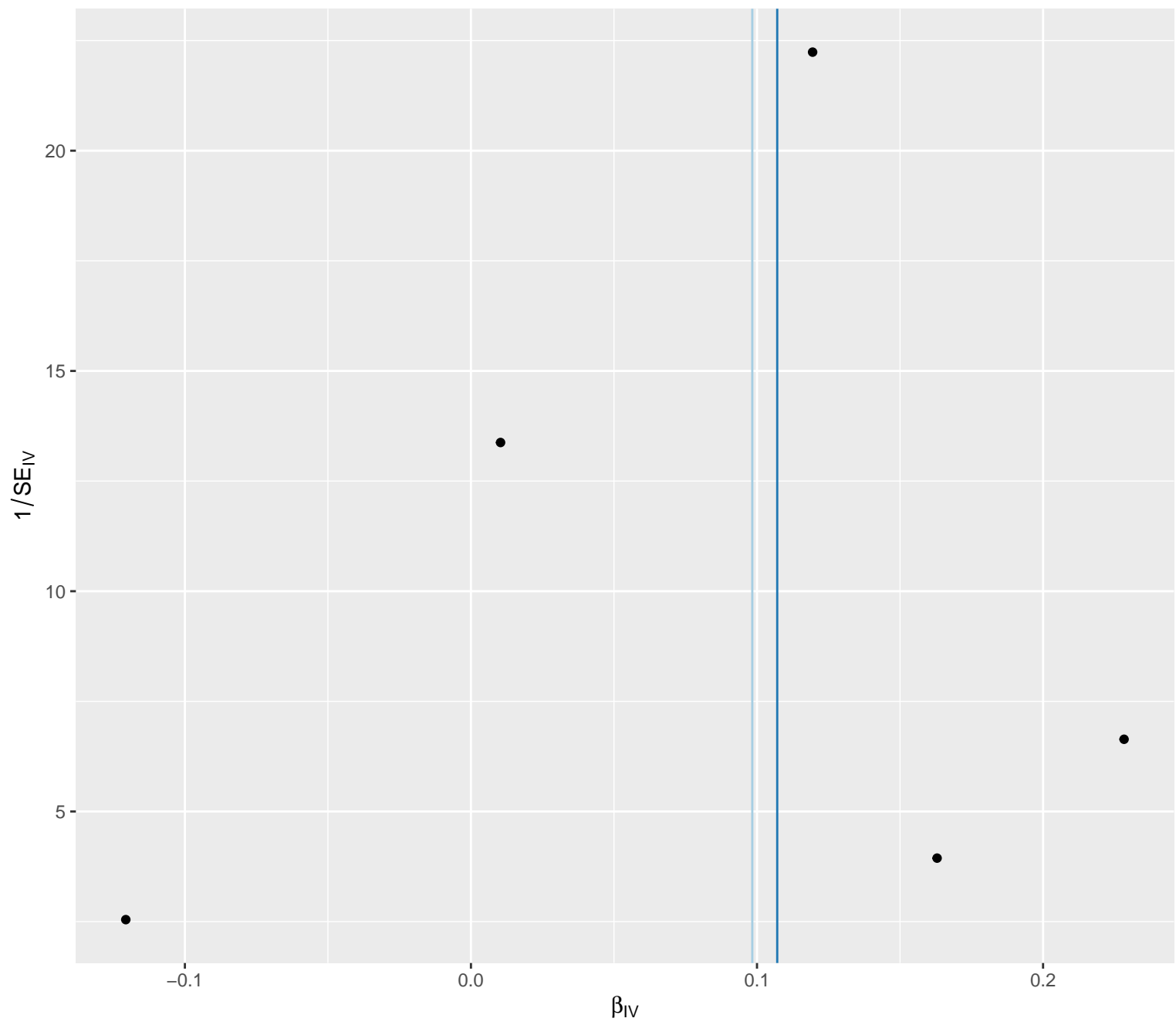

Supplement: Supplementary file 2 — Appendix S1. [file CNS-31-e70172-s001.zip › Supplementary File 1/1_Code and Data for Bio+MR/MF_Heterogeneity analysis/FunnelPlot/FGD3_H.pdf]

# MR Method

- Inverse variance weighted
- MR Egger

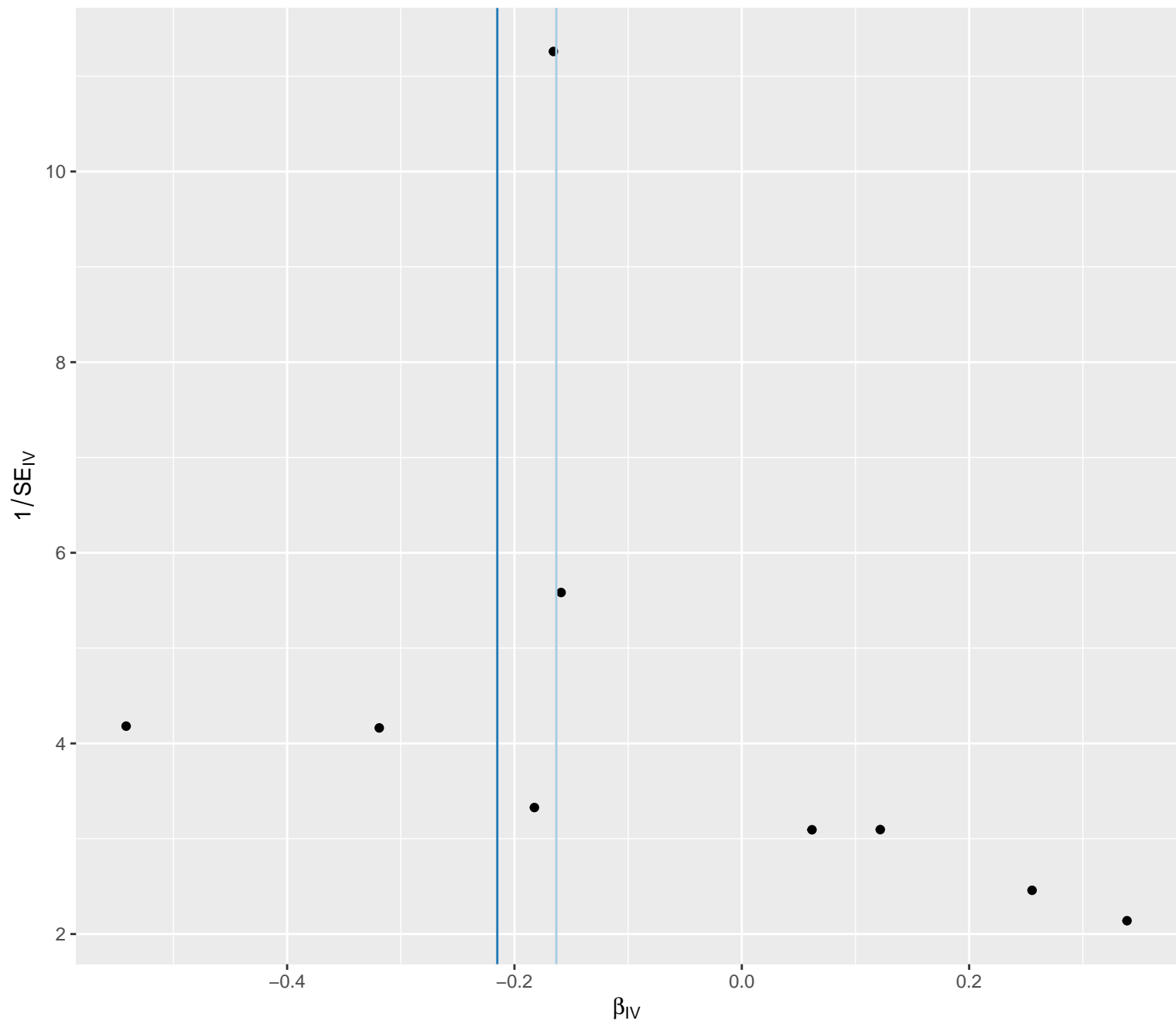

Supplement: Supplementary file 2 — Appendix S1. [file CNS-31-e70172-s001.zip › Supplementary File 1/1_Code and Data for Bio+MR/MF_Heterogeneity analysis/FunnelPlot/GZMA_H.pdf]

# MR Method

- Inverse variance weighted
- MR Egger

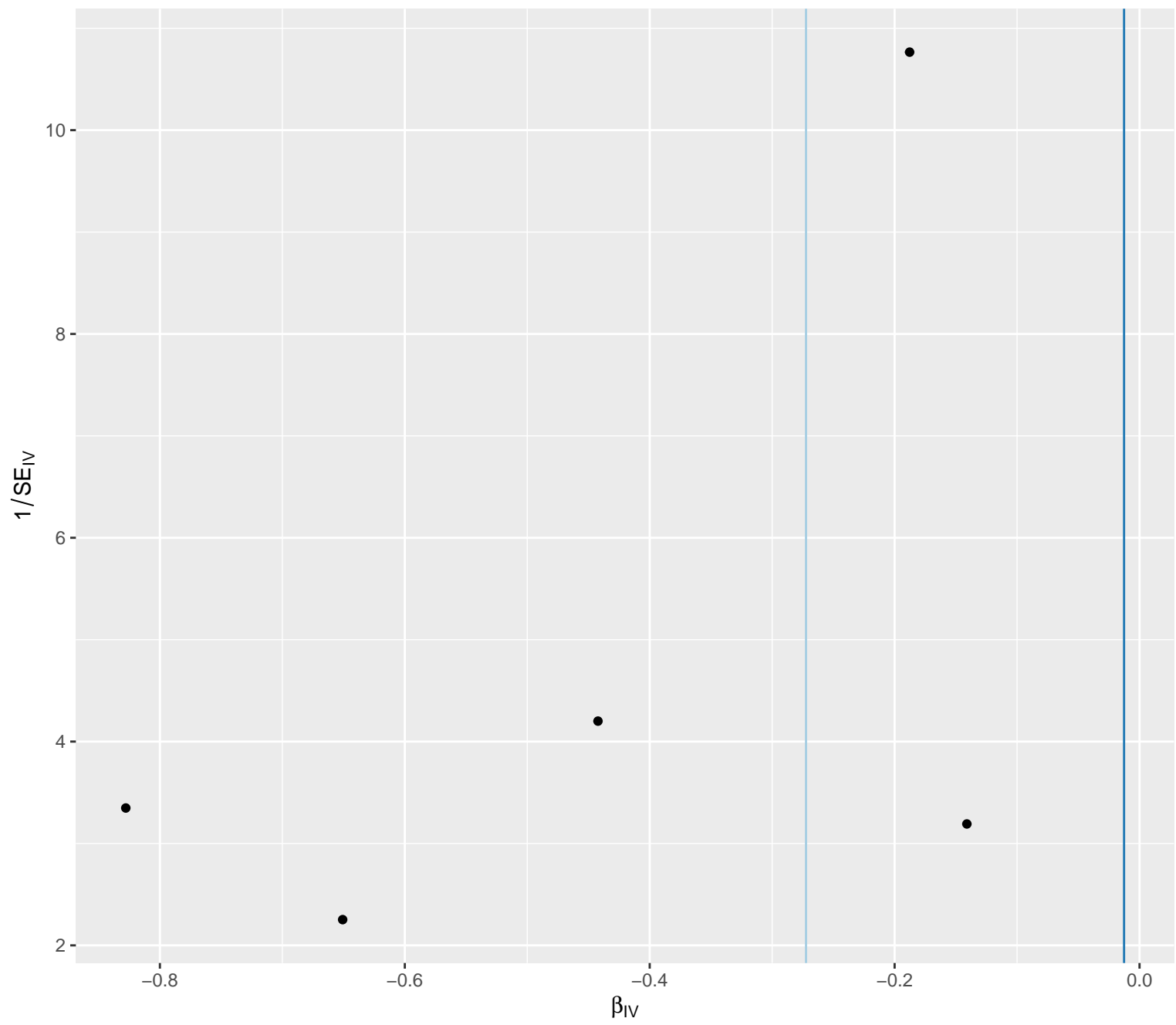

Supplement: Supplementary file 2 — Appendix S1. [file CNS-31-e70172-s001.zip › Supplementary File 1/1_Code and Data for Bio+MR/MF_Heterogeneity analysis/FunnelPlot/MTX1_H.pdf]

# MR Method

- Inverse variance weighted
- MR Egger

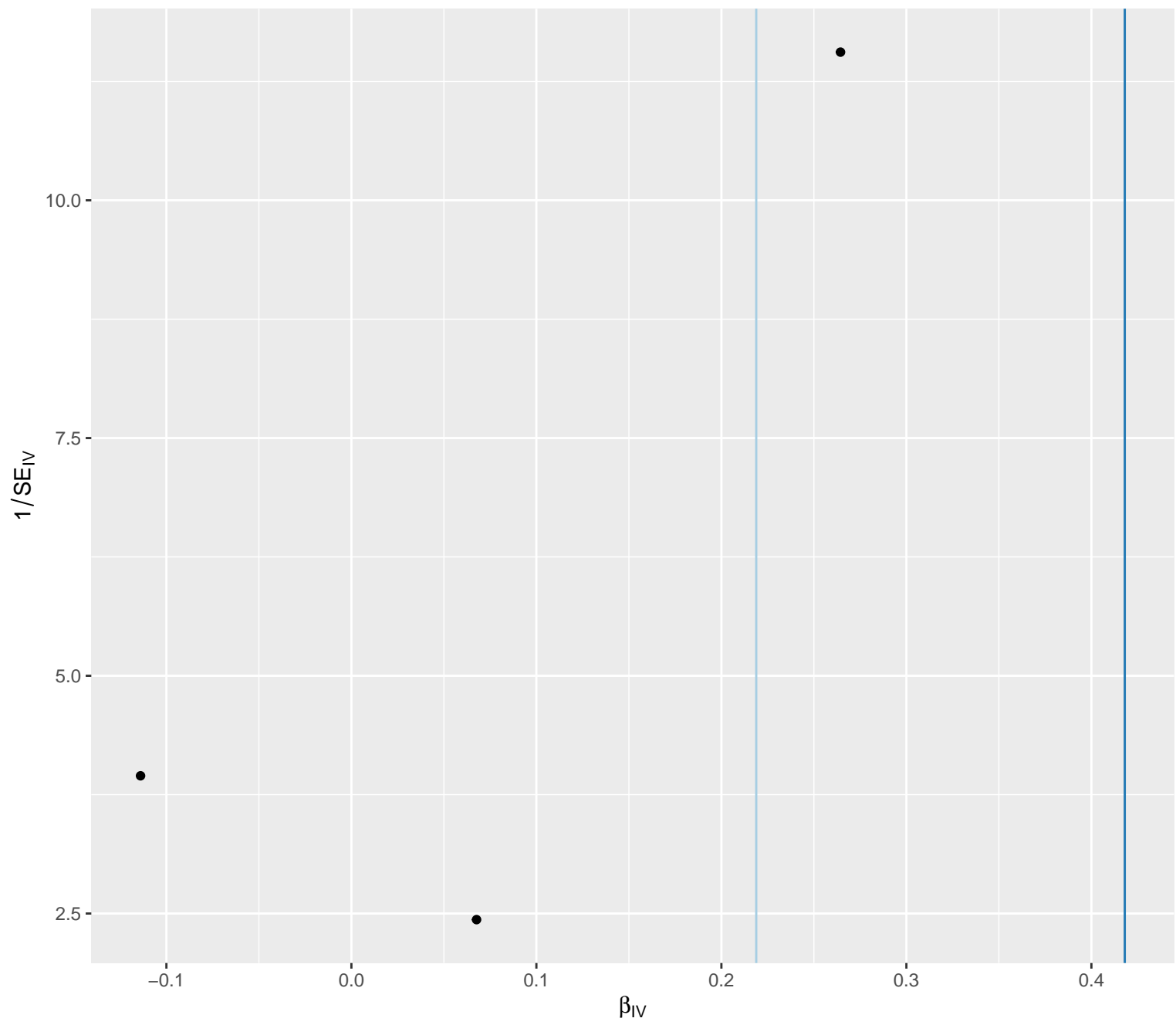

Supplement: Supplementary file 2 — Appendix S1. [file CNS-31-e70172-s001.zip › Supplementary File 1/1_Code and Data for Bio+MR/MF_Heterogeneity analysis/FunnelPlot/RAF1_H.pdf]

# MR Method

- Inverse variance weighted
- MR Egger

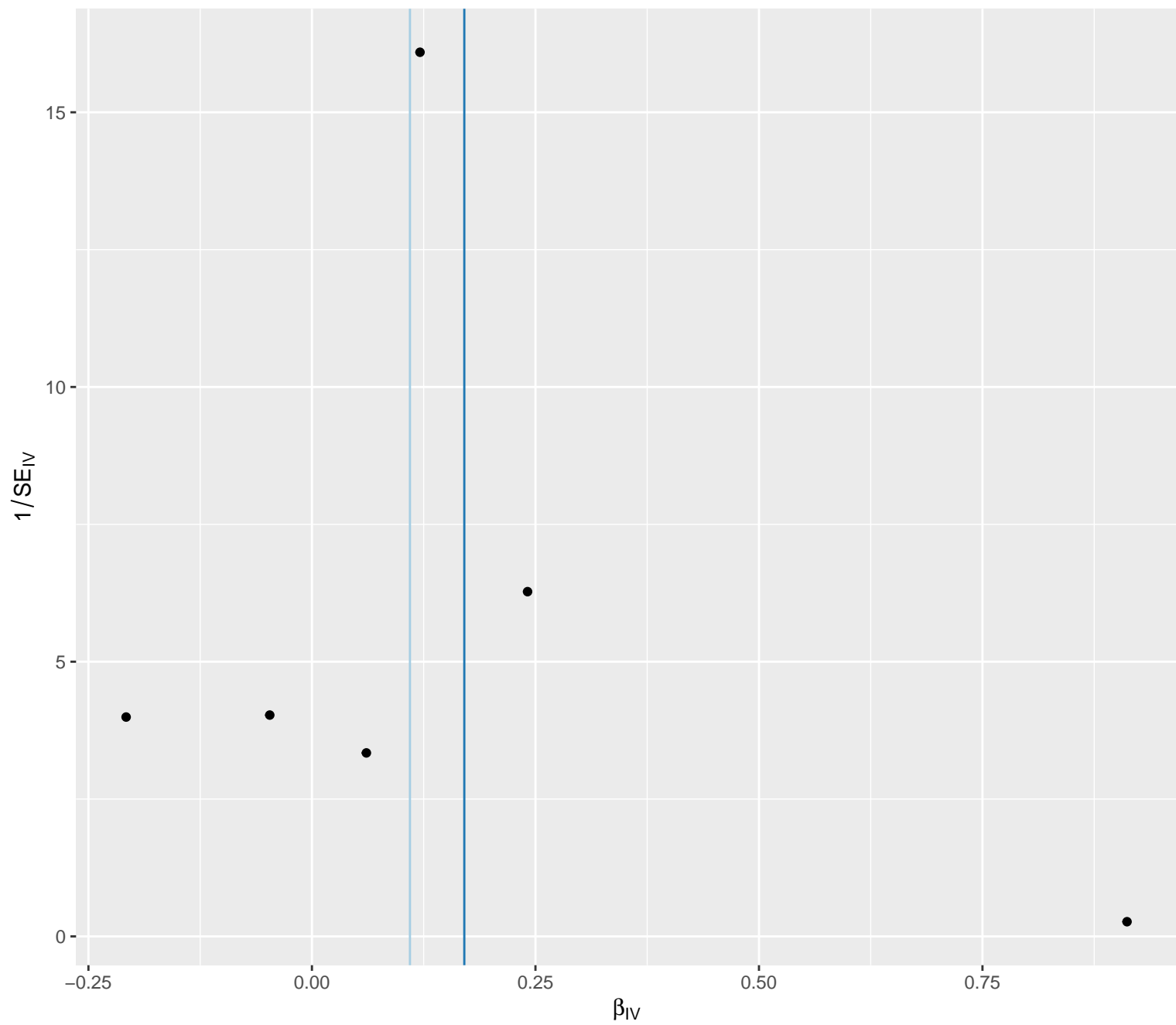

Supplement: Supplementary file 2 — Appendix S1. [file CNS-31-e70172-s001.zip › Supplementary File 1/1_Code and Data for Bio+MR/MF_Heterogeneity analysis/FunnelPlot/SH3BP5L_H.pdf]

# MR Method

- Inverse variance weighted
- MR Egger

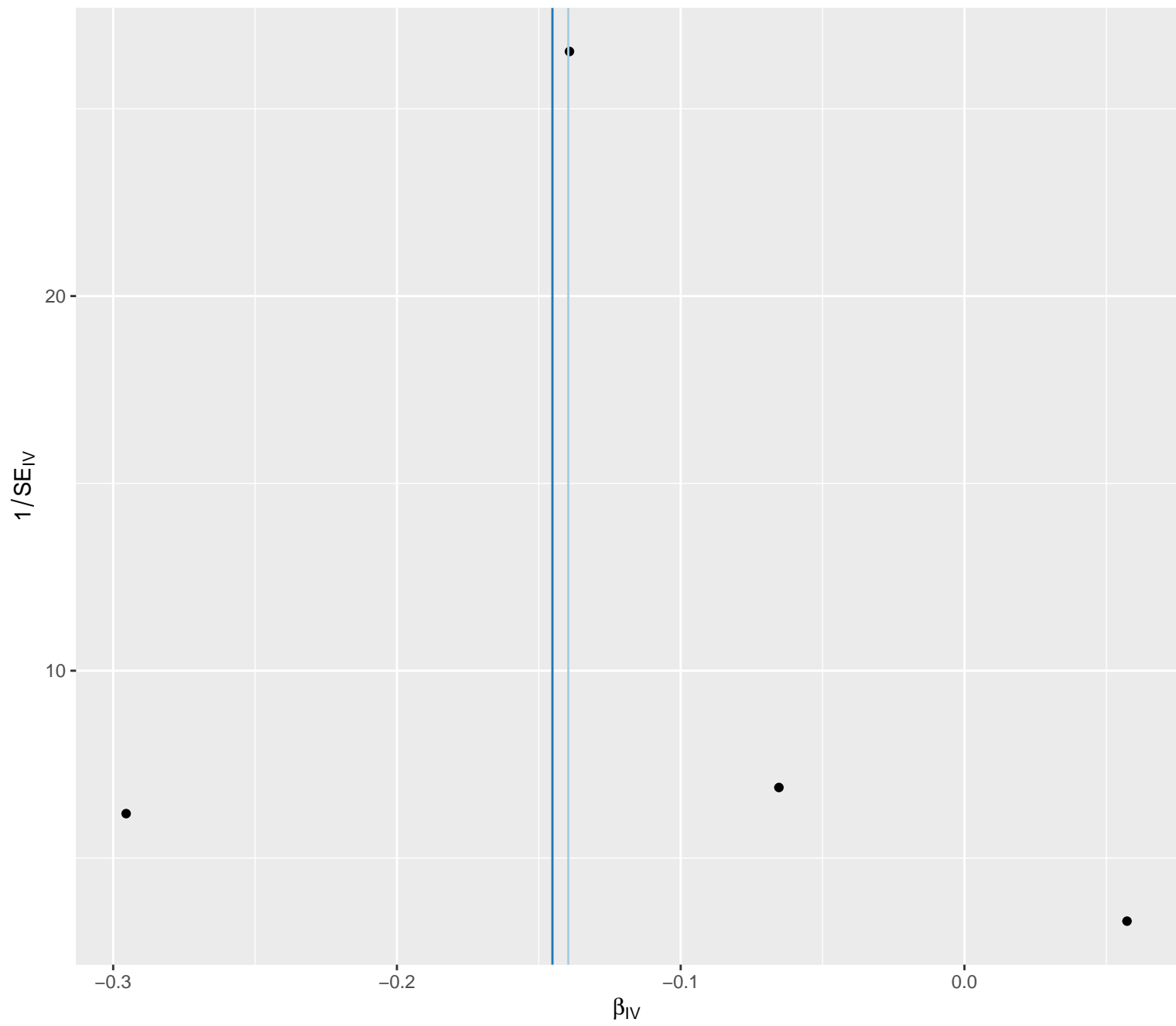

Supplement: Supplementary file 2 — Appendix S1. [file CNS-31-e70172-s001.zip › Supplementary File 1/1_Code and Data for Bio+MR/MF_Heterogeneity analysis/FunnelPlot/SSH2_H.pdf]

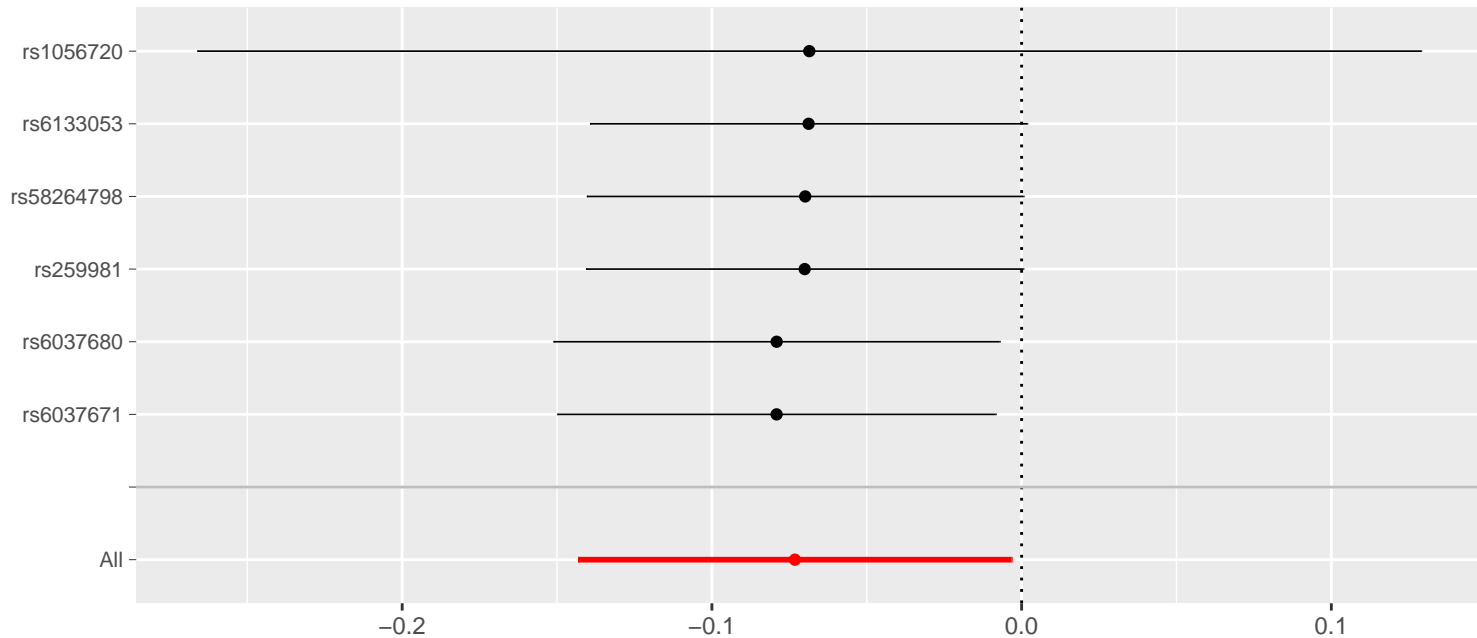

Supplement: Supplementary file 2 — Appendix S1. [file CNS-31-e70172-s001.zip › Supplementary File 1/1_Code and Data for Bio+MR/MF_Sensitive analysis/LeaveoneoutPlot/CDC25B_S.pdf]

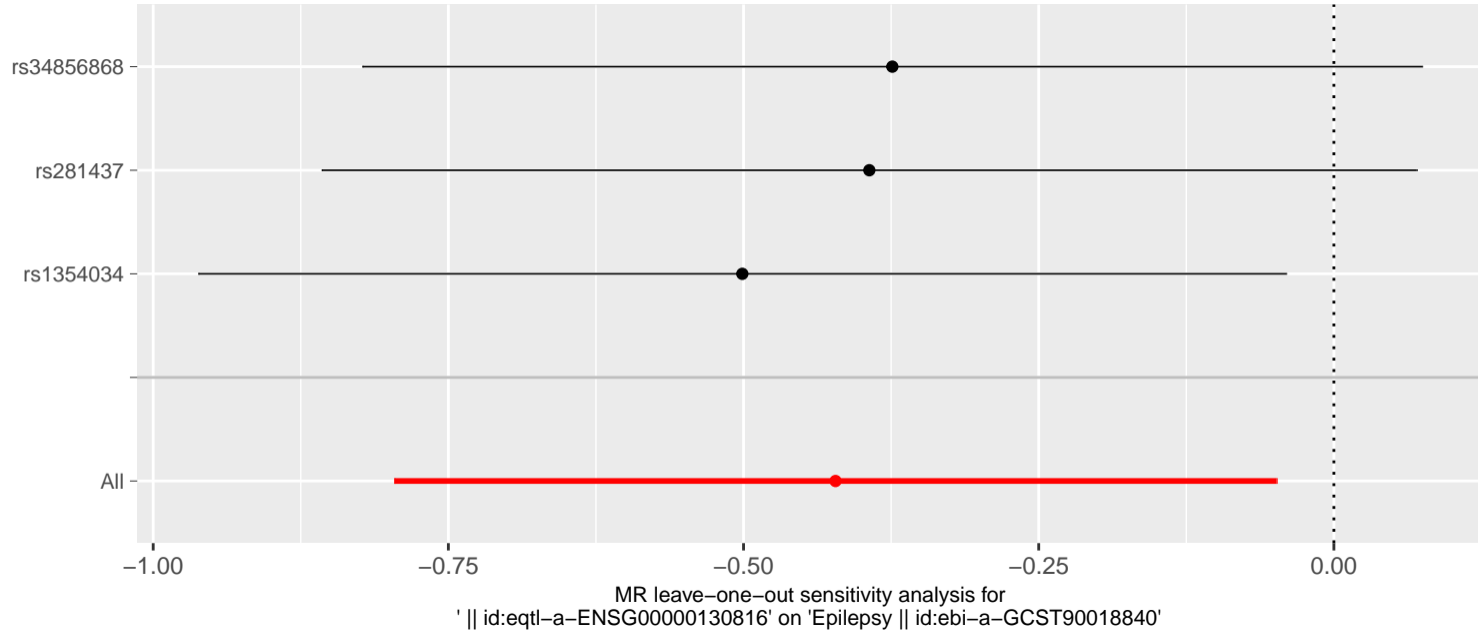

Supplement: Supplementary file 2 — Appendix S1. [file CNS-31-e70172-s001.zip › Supplementary File 1/1_Code and Data for Bio+MR/MF_Sensitive analysis/LeaveoneoutPlot/DNMT1_S.pdf]

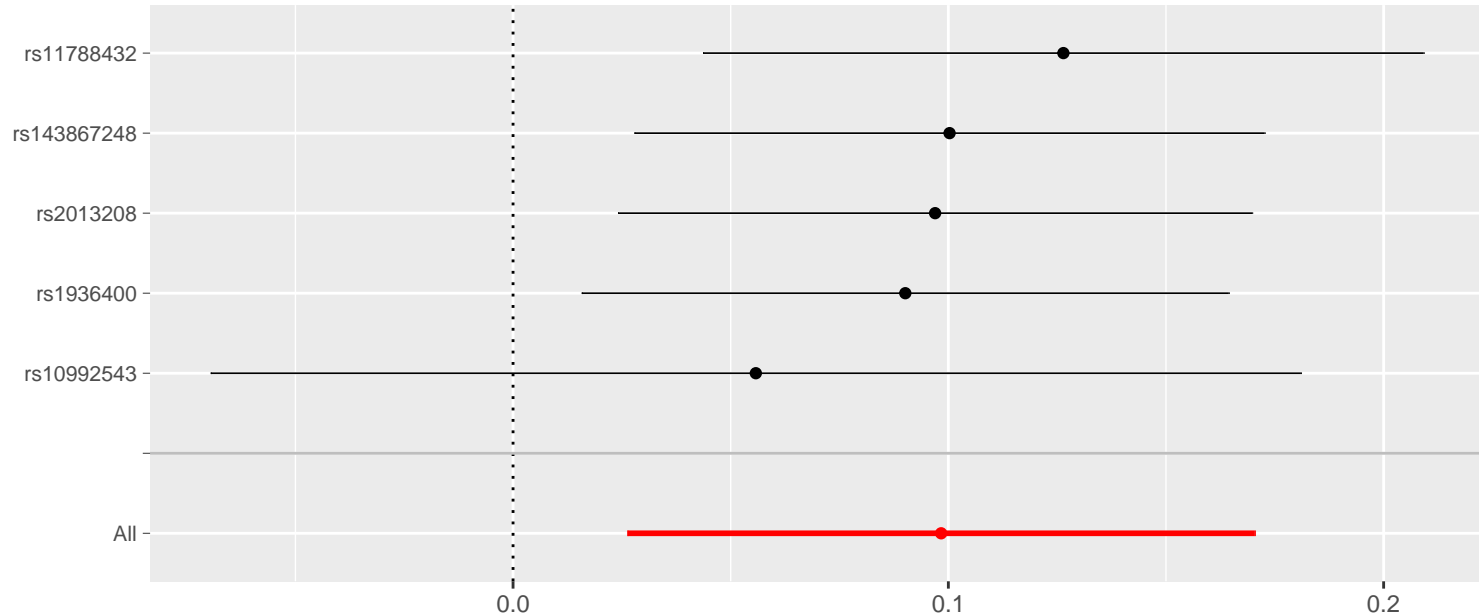

MR leave-one-out sensitivity analysis for  
' || id:eqtl-a-ENSG00000127084' on 'Epilepsy || id:ebi-a-GCST90018840'

Supplement: Supplementary file 2 — Appendix S1. [file CNS-31-e70172-s001.zip › Supplementary File 1/1_Code and Data for Bio+MR/MF_Sensitive analysis/LeaveoneoutPlot/FGD3_S.pdf]

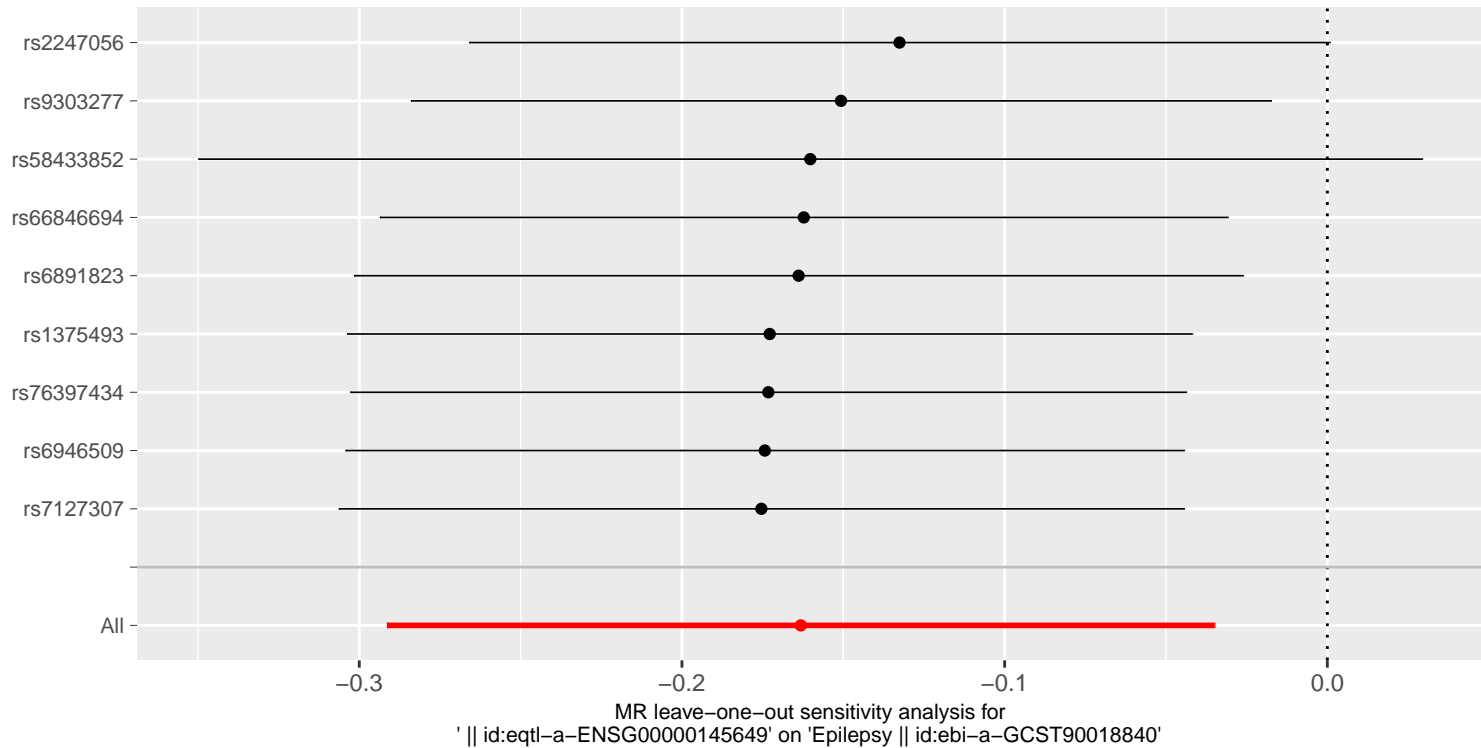

Supplement: Supplementary file 2 — Appendix S1. [file CNS-31-e70172-s001.zip › Supplementary File 1/1_Code and Data for Bio+MR/MF_Sensitive analysis/LeaveoneoutPlot/GZMA_S.pdf]

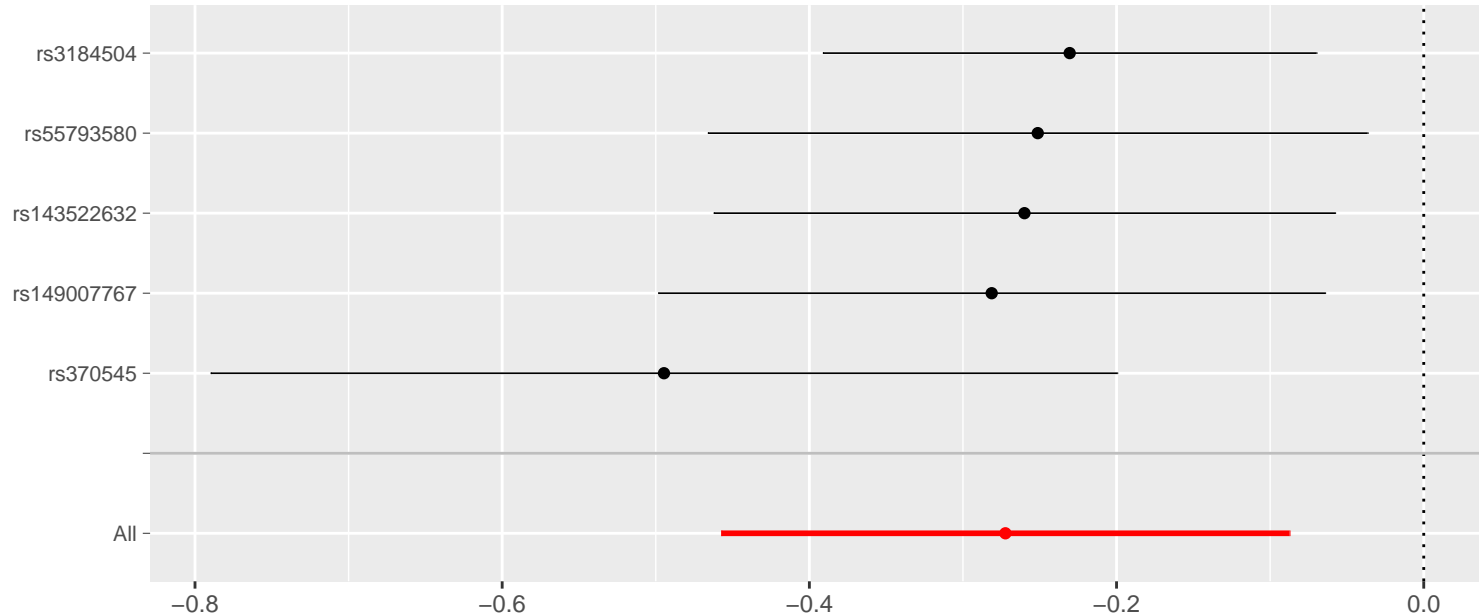

Supplement: Supplementary file 2 — Appendix S1. [file CNS-31-e70172-s001.zip › Supplementary File 1/1_Code and Data for Bio+MR/MF_Sensitive analysis/LeaveoneoutPlot/MTX1_S.pdf]

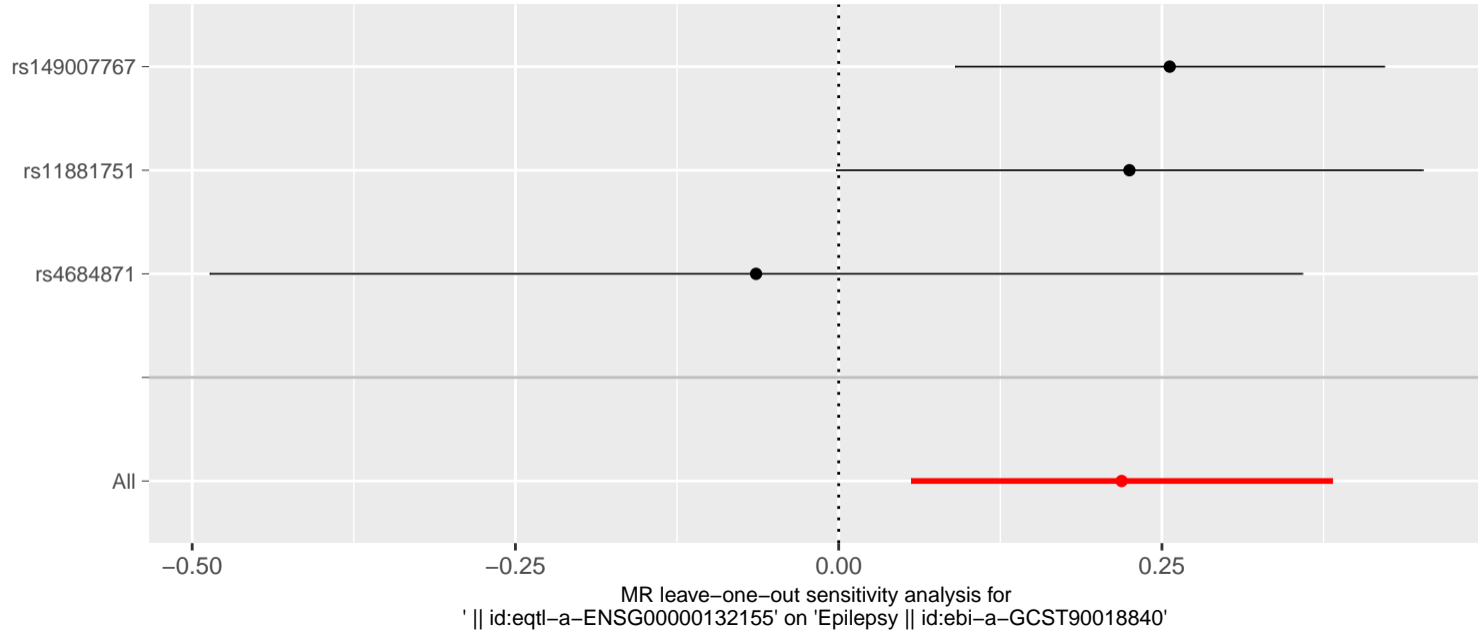

Supplement: Supplementary file 2 — Appendix S1. [file CNS-31-e70172-s001.zip › Supplementary File 1/1_Code and Data for Bio+MR/MF_Sensitive analysis/LeaveoneoutPlot/RAF1_S.pdf]

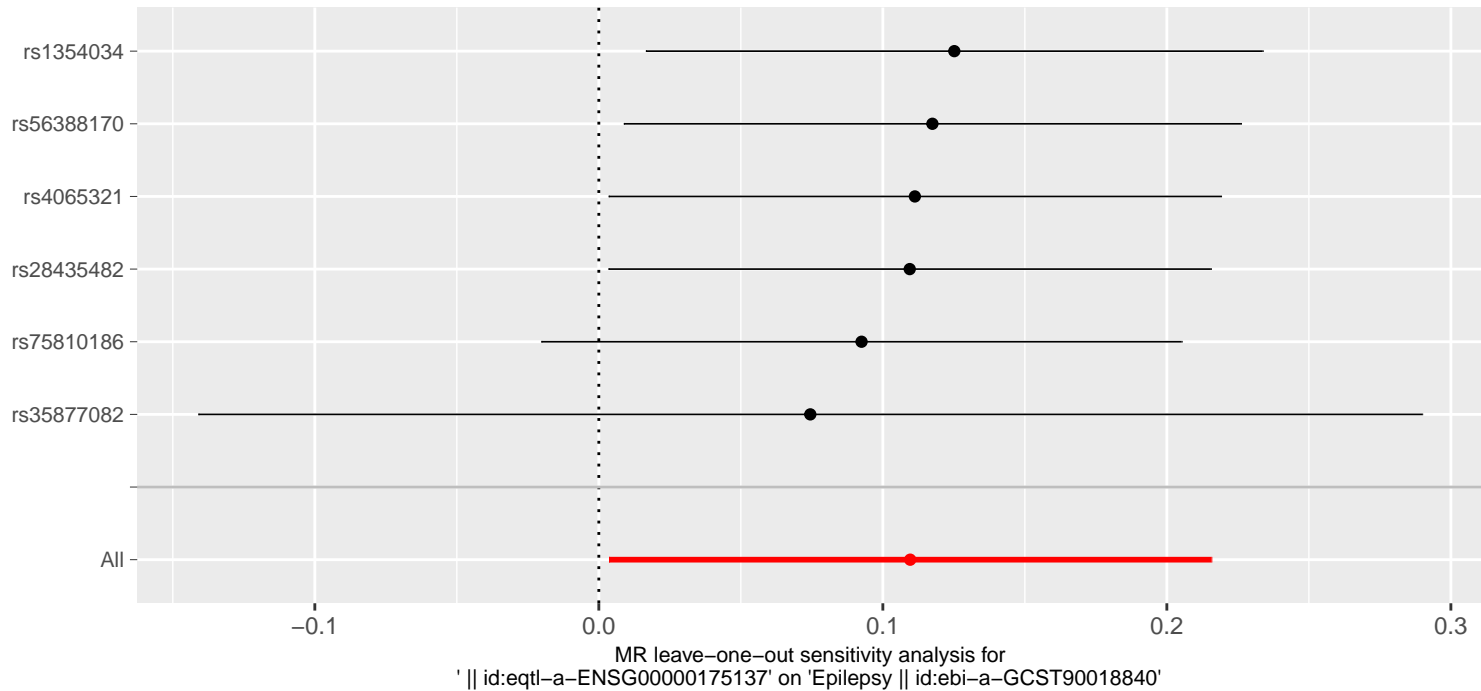

Supplement: Supplementary file 2 — Appendix S1. [file CNS-31-e70172-s001.zip › Supplementary File 1/1_Code and Data for Bio+MR/MF_Sensitive analysis/LeaveoneoutPlot/SH3BP5L_S.pdf]

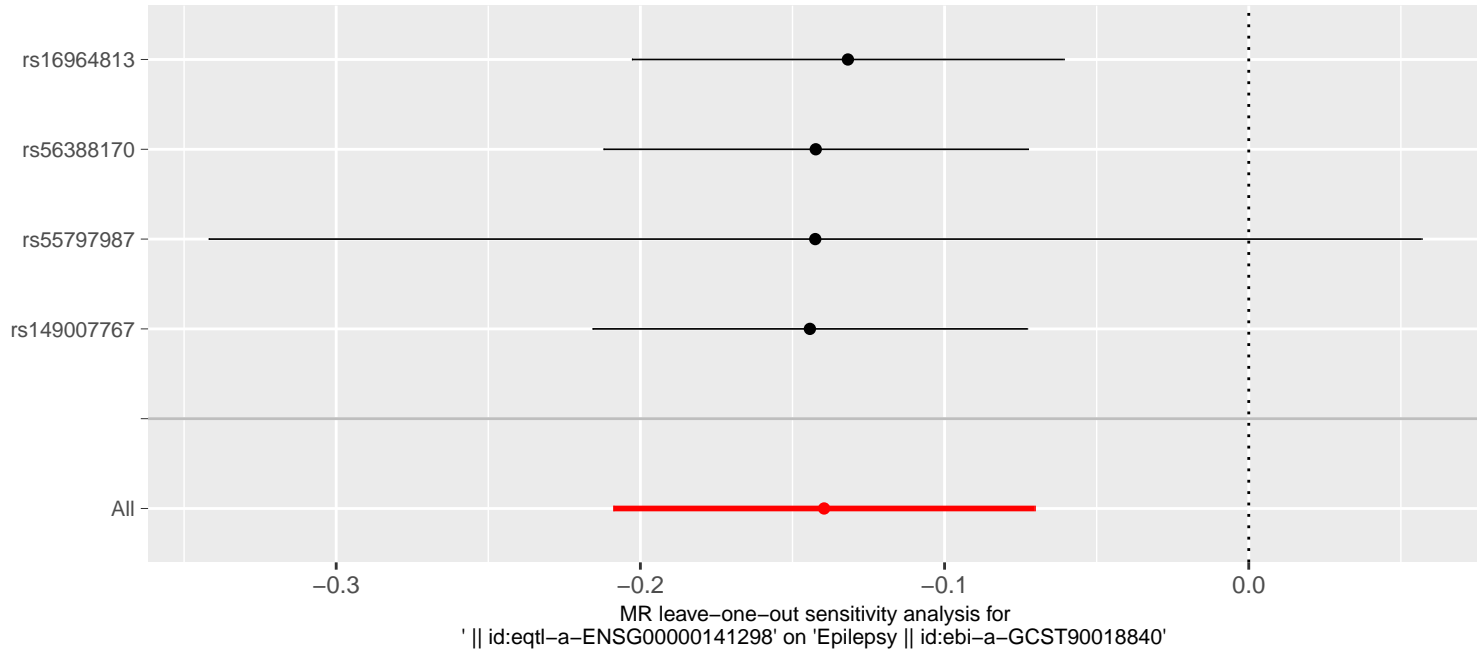

Supplement: Supplementary file 2 — Appendix S1. [file CNS-31-e70172-s001.zip › Supplementary File 1/1_Code and Data for Bio+MR/MF_Sensitive analysis/LeaveoneoutPlot/SSH2_S.pdf]

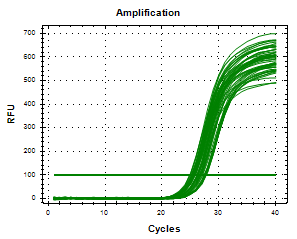

Supplement: Supplementary file 2 — Appendix S1. [file CNS-31-e70172-s001.zip › Supplementary File 1/2_qPCR/Amplification Curve/Batch 1_9x2/CDC25B_B1.png]

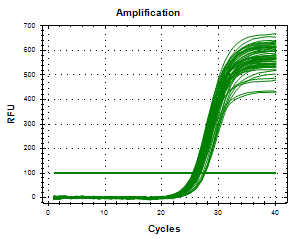

Supplement: Supplementary file 2 — Appendix S1. [file CNS-31-e70172-s001.zip › Supplementary File 1/2_qPCR/Amplification Curve/Batch 1_9x2/DNMT1_B1.png]

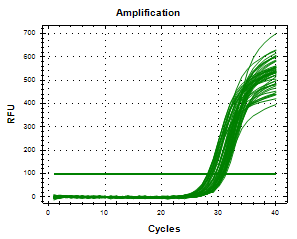

Supplement: Supplementary file 2 — Appendix S1. [file CNS-31-e70172-s001.zip › Supplementary File 1/2_qPCR/Amplification Curve/Batch 1_9x2/FGD3_B1.png]

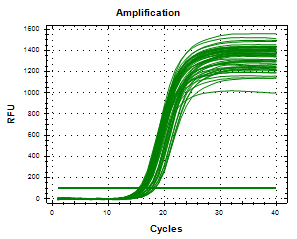

Supplement: Supplementary file 2 — Appendix S1. [file CNS-31-e70172-s001.zip › Supplementary File 1/2_qPCR/Amplification Curve/Batch 1_9x2/GAPDH_B1.png]

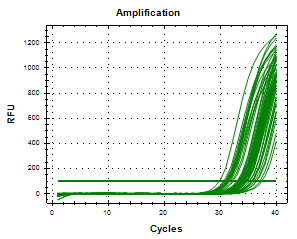

Supplement: Supplementary file 2 — Appendix S1. [file CNS-31-e70172-s001.zip › Supplementary File 1/2_qPCR/Amplification Curve/Batch 1_9x2/GZMA_B1.png]

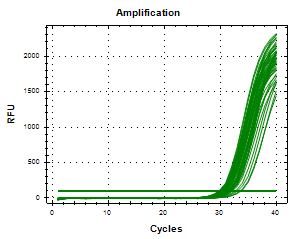

Supplement: Supplementary file 2 — Appendix S1. [file CNS-31-e70172-s001.zip › Supplementary File 1/2_qPCR/Amplification Curve/Batch 1_9x2/MTX1_B1.png]

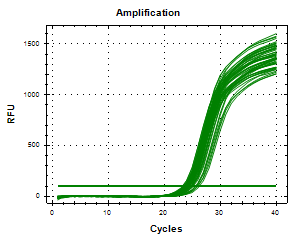

Supplement: Supplementary file 2 — Appendix S1. [file CNS-31-e70172-s001.zip › Supplementary File 1/2_qPCR/Amplification Curve/Batch 1_9x2/RAF1_B1.png]

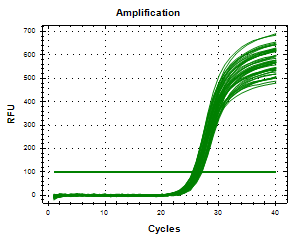

Supplement: Supplementary file 2 — Appendix S1. [file CNS-31-e70172-s001.zip › Supplementary File 1/2_qPCR/Amplification Curve/Batch 1_9x2/sh3bp5l_B1.png]

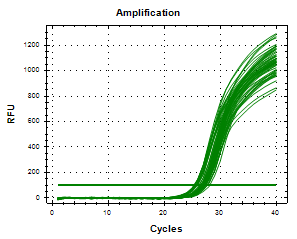

Supplement: Supplementary file 2 — Appendix S1. [file CNS-31-e70172-s001.zip › Supplementary File 1/2_qPCR/Amplification Curve/Batch 1_9x2/SSH2_B1.png]

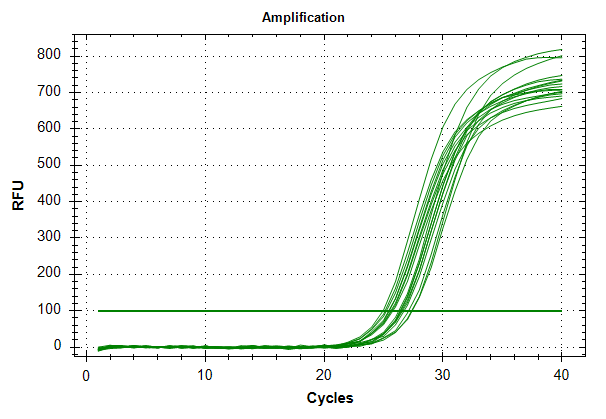

Supplement: Supplementary file 2 — Appendix S1. [file CNS-31-e70172-s001.zip › Supplementary File 1/2_qPCR/Amplification Curve/Batch 2_3x2/CDC25B_B2.png]

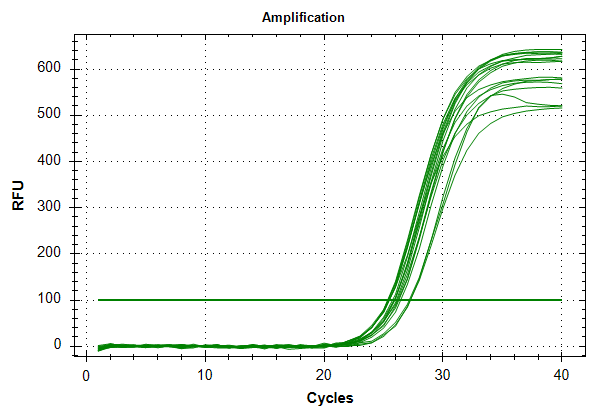

Supplement: Supplementary file 2 — Appendix S1. [file CNS-31-e70172-s001.zip › Supplementary File 1/2_qPCR/Amplification Curve/Batch 2_3x2/DNMT1_B2.png]

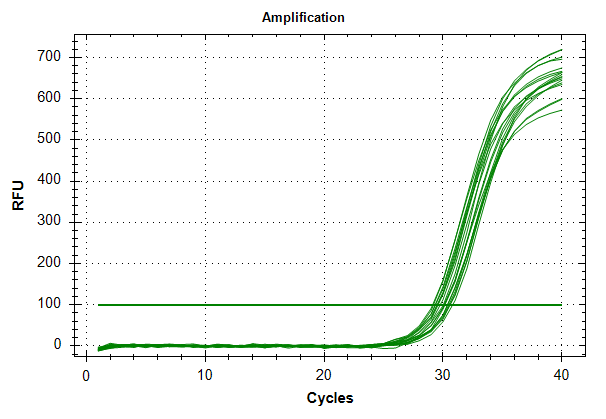

Supplement: Supplementary file 2 — Appendix S1. [file CNS-31-e70172-s001.zip › Supplementary File 1/2_qPCR/Amplification Curve/Batch 2_3x2/FGD3_B2.png]

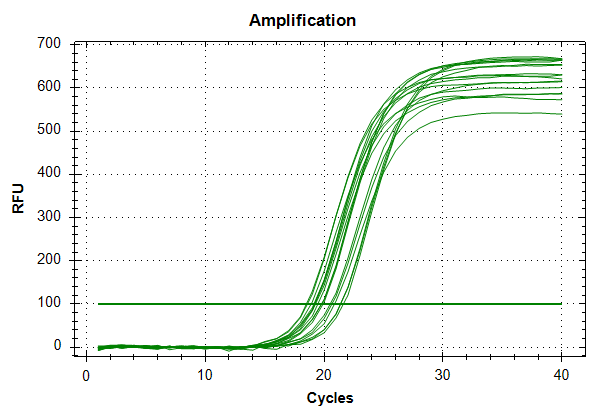

Supplement: Supplementary file 2 — Appendix S1. [file CNS-31-e70172-s001.zip › Supplementary File 1/2_qPCR/Amplification Curve/Batch 2_3x2/GAPDH_B2.png]

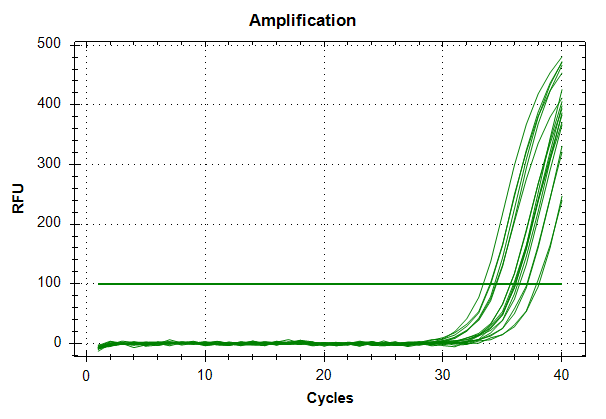

Supplement: Supplementary file 2 — Appendix S1. [file CNS-31-e70172-s001.zip › Supplementary File 1/2_qPCR/Amplification Curve/Batch 2_3x2/GZMA_B2.png]

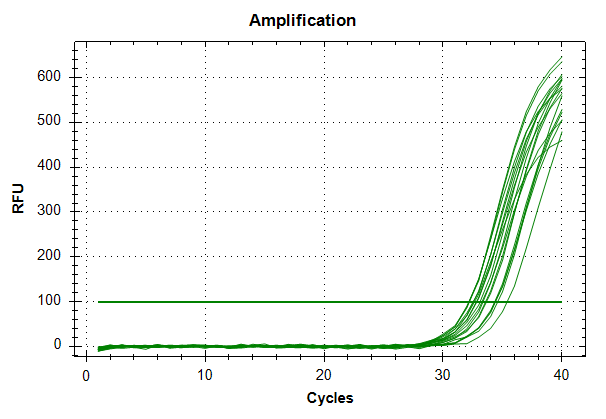

Supplement: Supplementary file 2 — Appendix S1. [file CNS-31-e70172-s001.zip › Supplementary File 1/2_qPCR/Amplification Curve/Batch 2_3x2/MTX1_B2.png]

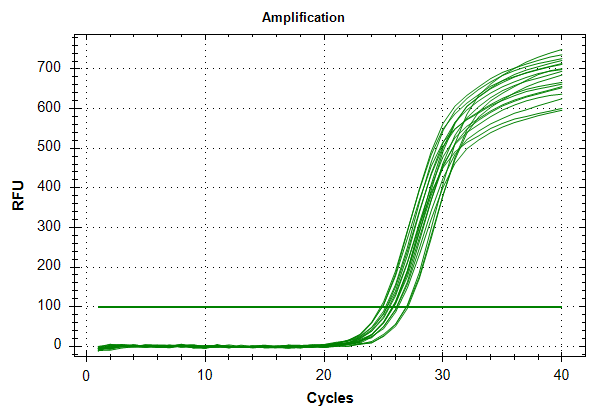

Supplement: Supplementary file 2 — Appendix S1. [file CNS-31-e70172-s001.zip › Supplementary File 1/2_qPCR/Amplification Curve/Batch 2_3x2/RAF1_B2.png]

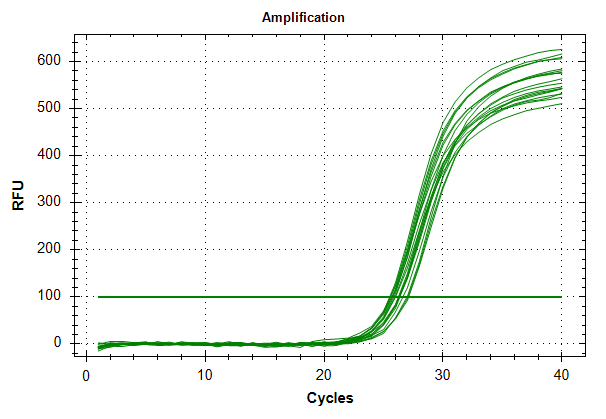

Supplement: Supplementary file 2 — Appendix S1. [file CNS-31-e70172-s001.zip › Supplementary File 1/2_qPCR/Amplification Curve/Batch 2_3x2/SH3BP5L_B2.png]

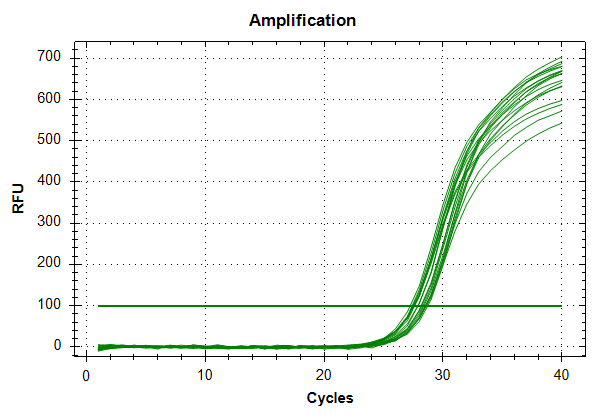

Supplement: Supplementary file 2 — Appendix S1. [file CNS-31-e70172-s001.zip › Supplementary File 1/2_qPCR/Amplification Curve/Batch 2_3x2/SSH2_B2.png]

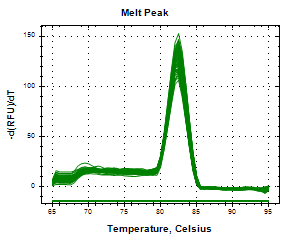

Supplement: Supplementary file 2 — Appendix S1. [file CNS-31-e70172-s001.zip › Supplementary File 1/2_qPCR/Melting Curve/Batch 1_9x2/CDC25B_M1.png]

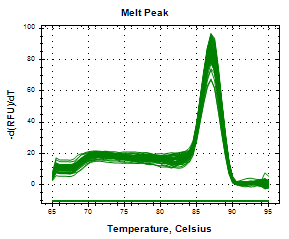

Supplement: Supplementary file 2 — Appendix S1. [file CNS-31-e70172-s001.zip › Supplementary File 1/2_qPCR/Melting Curve/Batch 1_9x2/DNMT1_M1.png]

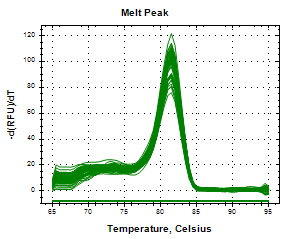

Supplement: Supplementary file 2 — Appendix S1. [file CNS-31-e70172-s001.zip › Supplementary File 1/2_qPCR/Melting Curve/Batch 1_9x2/FGD3_M1.png]

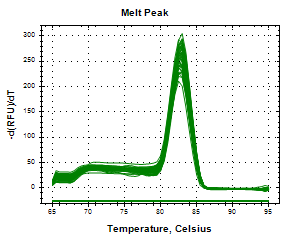

Supplement: Supplementary file 2 — Appendix S1. [file CNS-31-e70172-s001.zip › Supplementary File 1/2_qPCR/Melting Curve/Batch 1_9x2/GAPDH_M1.png]

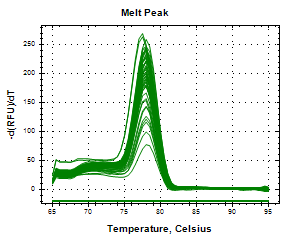

Supplement: Supplementary file 2 — Appendix S1. [file CNS-31-e70172-s001.zip › Supplementary File 1/2_qPCR/Melting Curve/Batch 1_9x2/GZMA_M1.png]

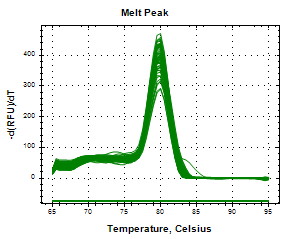

Supplement: Supplementary file 2 — Appendix S1. [file CNS-31-e70172-s001.zip › Supplementary File 1/2_qPCR/Melting Curve/Batch 1_9x2/MTX1_M1.png]

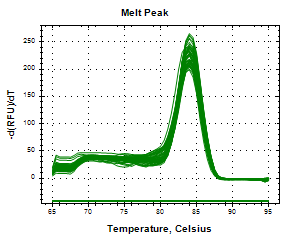

Supplement: Supplementary file 2 — Appendix S1. [file CNS-31-e70172-s001.zip › Supplementary File 1/2_qPCR/Melting Curve/Batch 1_9x2/RAF1_M1.png]

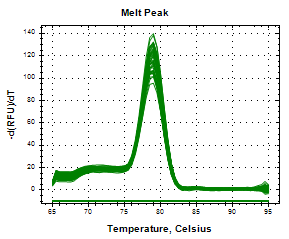

Supplement: Supplementary file 2 — Appendix S1. [file CNS-31-e70172-s001.zip › Supplementary File 1/2_qPCR/Melting Curve/Batch 1_9x2/sh3bp5l_M1.png]

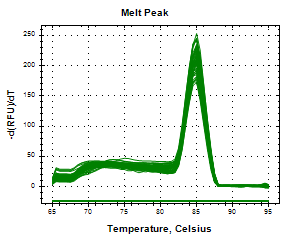

Supplement: Supplementary file 2 — Appendix S1. [file CNS-31-e70172-s001.zip › Supplementary File 1/2_qPCR/Melting Curve/Batch 1_9x2/SSH2_M1.png]

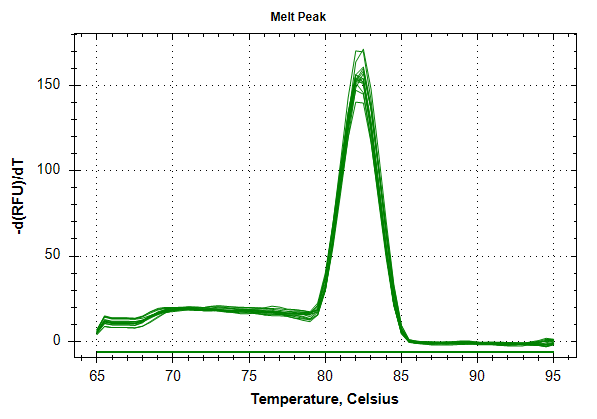

Supplement: Supplementary file 2 — Appendix S1. [file CNS-31-e70172-s001.zip › Supplementary File 1/2_qPCR/Melting Curve/Batch 2_3x2/CDC25B_M2.png]

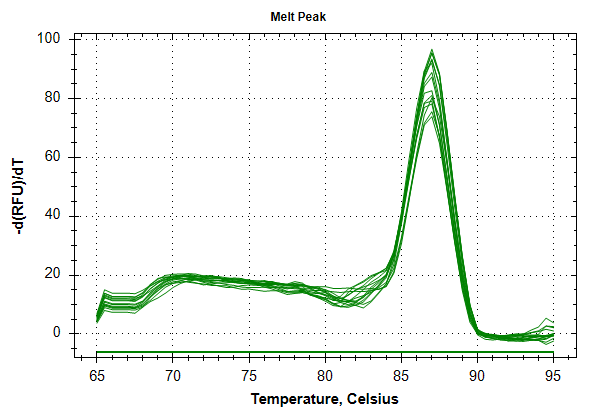

Supplement: Supplementary file 2 — Appendix S1. [file CNS-31-e70172-s001.zip › Supplementary File 1/2_qPCR/Melting Curve/Batch 2_3x2/DNMT1_M2.png]

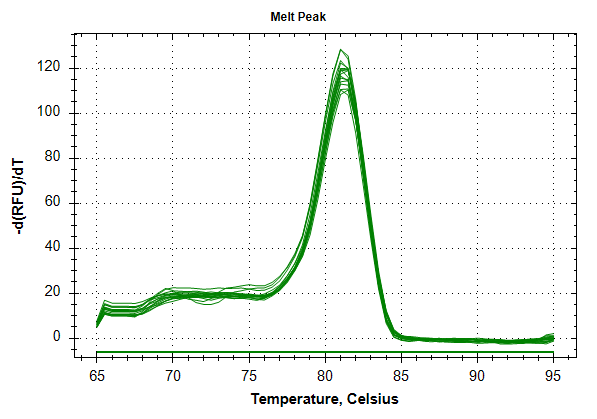

Supplement: Supplementary file 2 — Appendix S1. [file CNS-31-e70172-s001.zip › Supplementary File 1/2_qPCR/Melting Curve/Batch 2_3x2/FGD3_M2.png]

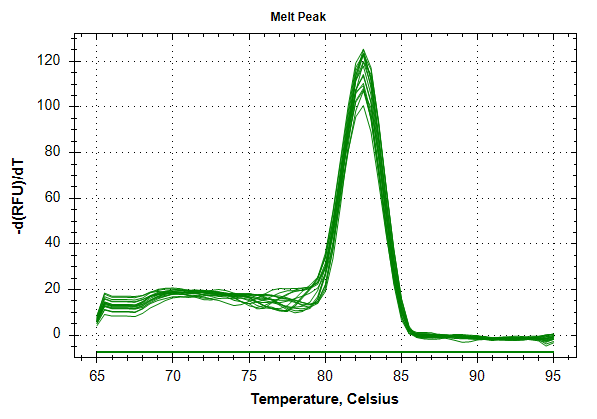

Supplement: Supplementary file 2 — Appendix S1. [file CNS-31-e70172-s001.zip › Supplementary File 1/2_qPCR/Melting Curve/Batch 2_3x2/GAPDH_M2.png]

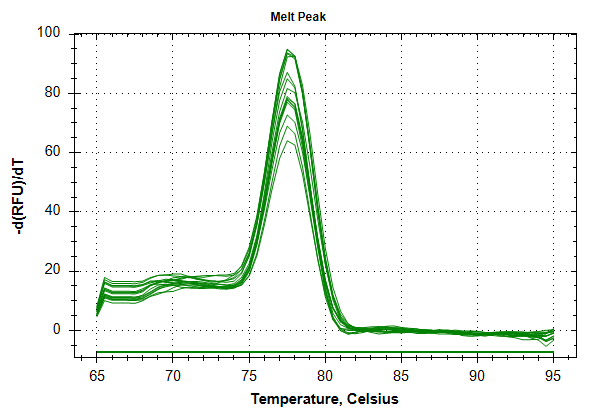

Supplement: Supplementary file 2 — Appendix S1. [file CNS-31-e70172-s001.zip › Supplementary File 1/2_qPCR/Melting Curve/Batch 2_3x2/GZMA_M2.png]

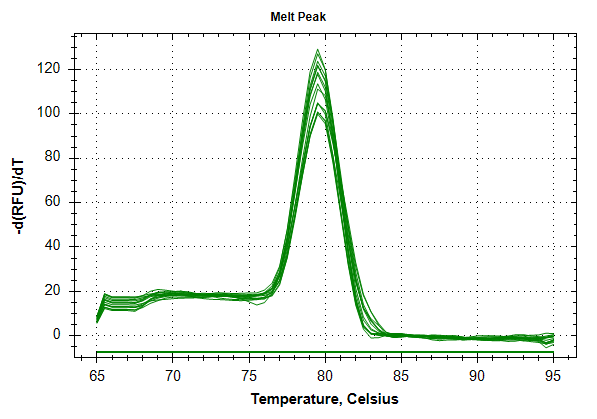

Supplement: Supplementary file 2 — Appendix S1. [file CNS-31-e70172-s001.zip › Supplementary File 1/2_qPCR/Melting Curve/Batch 2_3x2/MTX1_M2.png]

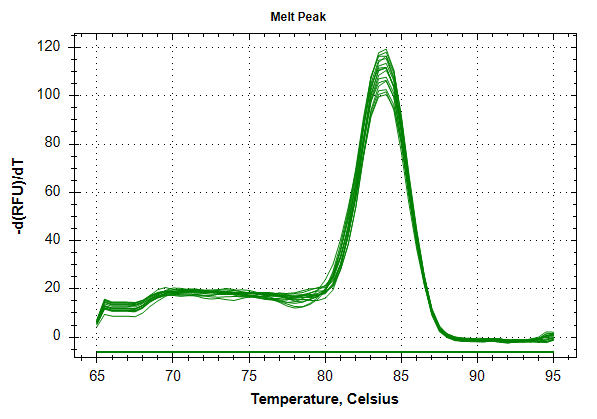

Supplement: Supplementary file 2 — Appendix S1. [file CNS-31-e70172-s001.zip › Supplementary File 1/2_qPCR/Melting Curve/Batch 2_3x2/RAF1_M2.png]

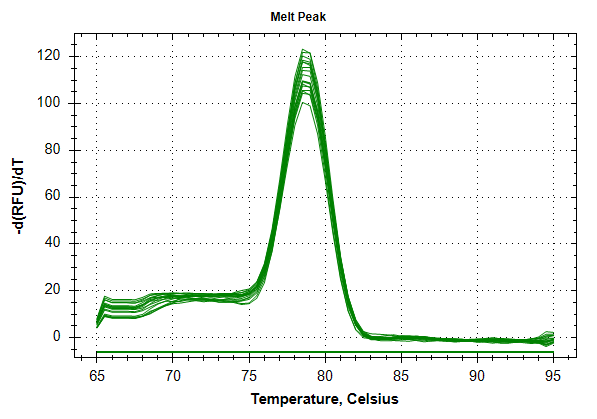

Supplement: Supplementary file 2 — Appendix S1. [file CNS-31-e70172-s001.zip › Supplementary File 1/2_qPCR/Melting Curve/Batch 2_3x2/SH3BP5L_M2.png]

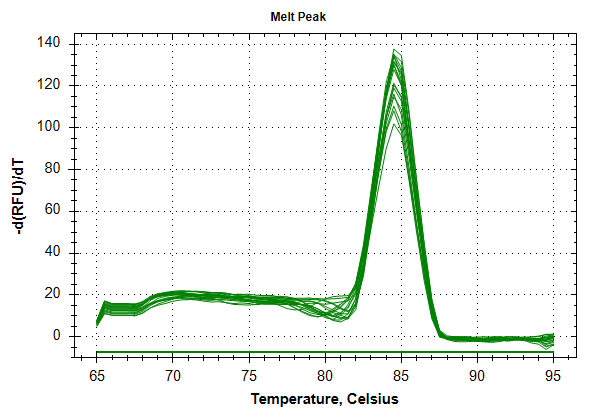

Supplement: Supplementary file 2 — Appendix S1. [file CNS-31-e70172-s001.zip › Supplementary File 1/2_qPCR/Melting Curve/Batch 2_3x2/SSH2_M2.png]

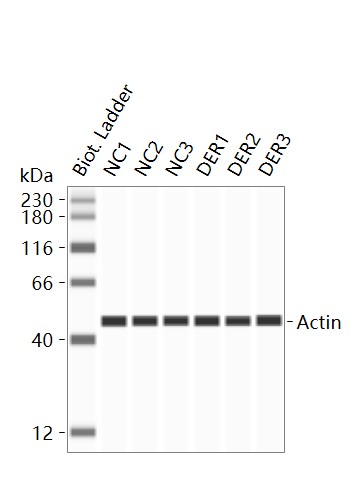

Supplement: Supplementary file 2 — Appendix S1. [file CNS-31-e70172-s001.zip › Supplementary File 1/3_WB/Digital WB/Actin_WB.jpg]

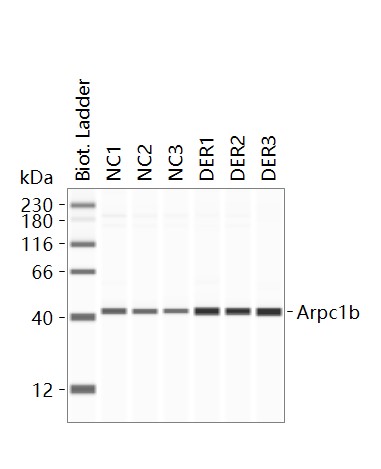

Supplement: Supplementary file 2 — Appendix S1. [file CNS-31-e70172-s001.zip › Supplementary File 1/3_WB/Digital WB/Arpc1b_WB.jpg]

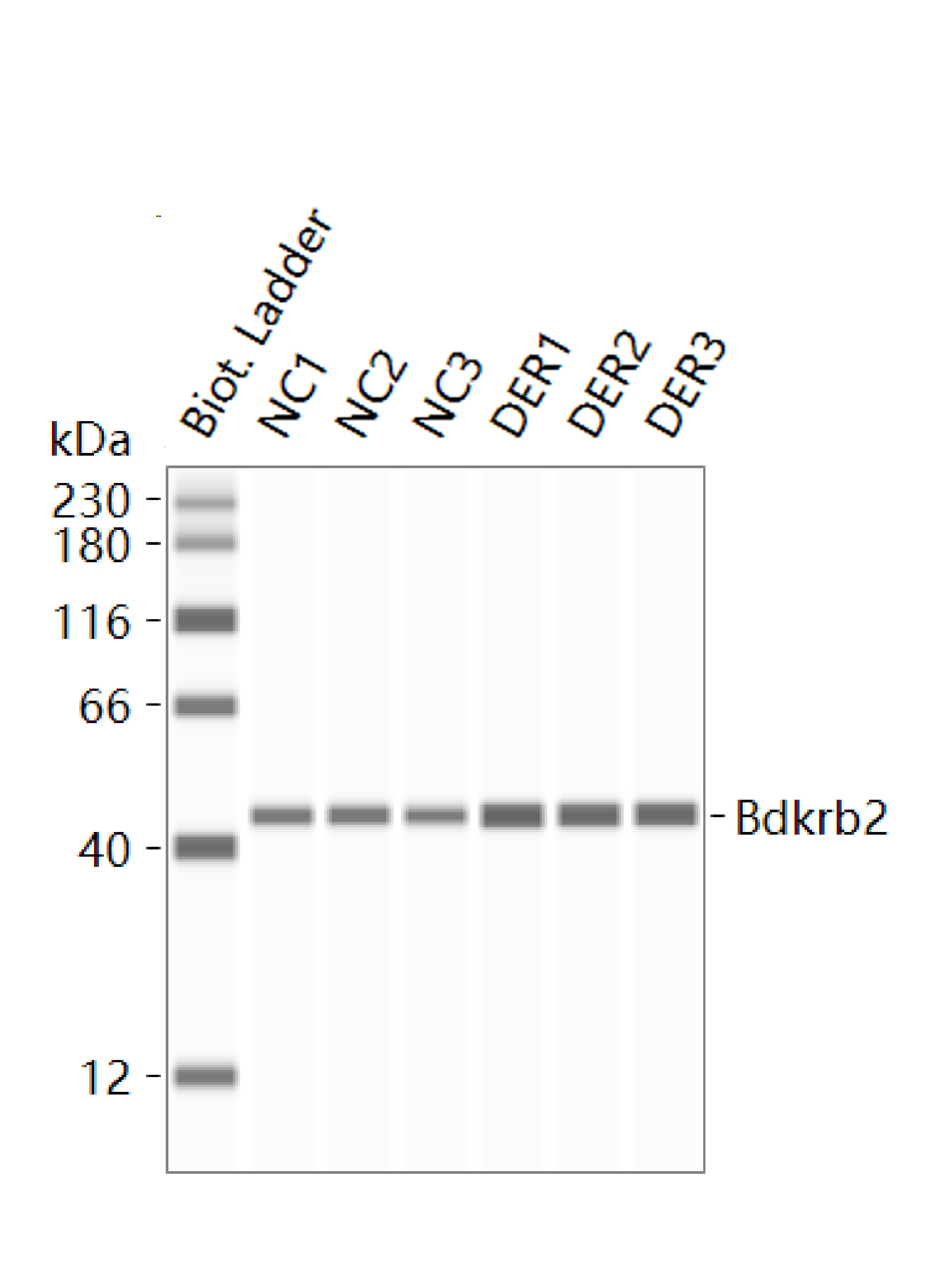

Supplement: Supplementary file 2 — Appendix S1. [file CNS-31-e70172-s001.zip › Supplementary File 1/3_WB/Digital WB/bdkrb2_WB.jpg]

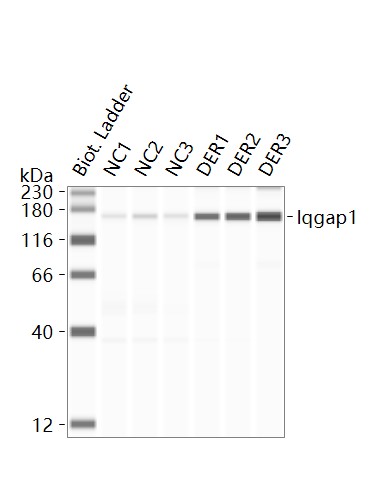

Supplement: Supplementary file 2 — Appendix S1. [file CNS-31-e70172-s001.zip › Supplementary File 1/3_WB/Digital WB/Iqgap1_WB.jpg]

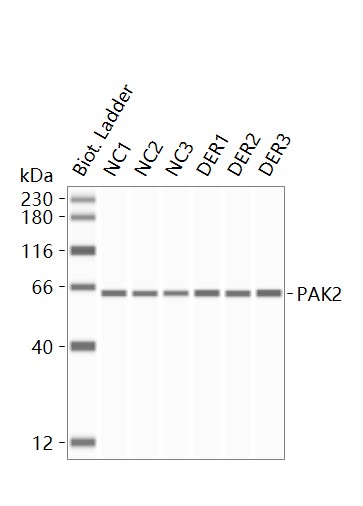

Supplement: Supplementary file 2 — Appendix S1. [file CNS-31-e70172-s001.zip › Supplementary File 1/3_WB/Digital WB/Pak2_WB.jpg]

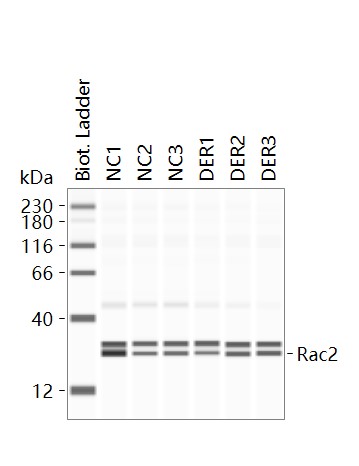

Supplement: Supplementary file 2 — Appendix S1. [file CNS-31-e70172-s001.zip › Supplementary File 1/3_WB/Digital WB/Rac2_WB.jpg]

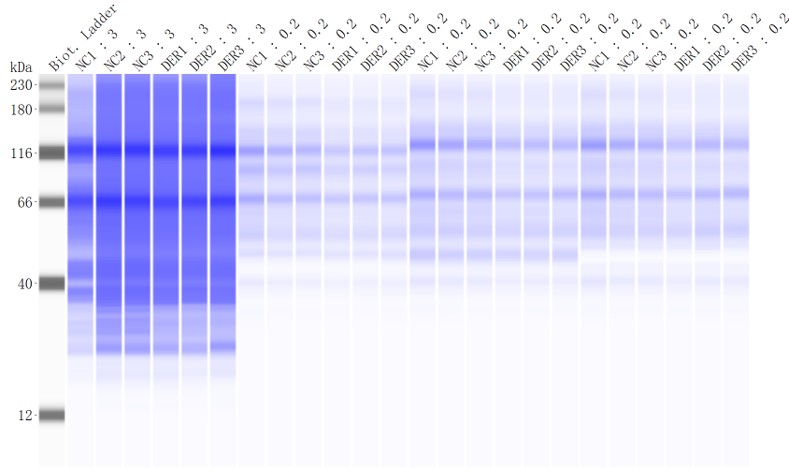

Supplement: Supplementary file 2 — Appendix S1. [file CNS-31-e70172-s001.zip › Supplementary File 1/3_WB/Digital WB/Total Protein_WB.jpg]

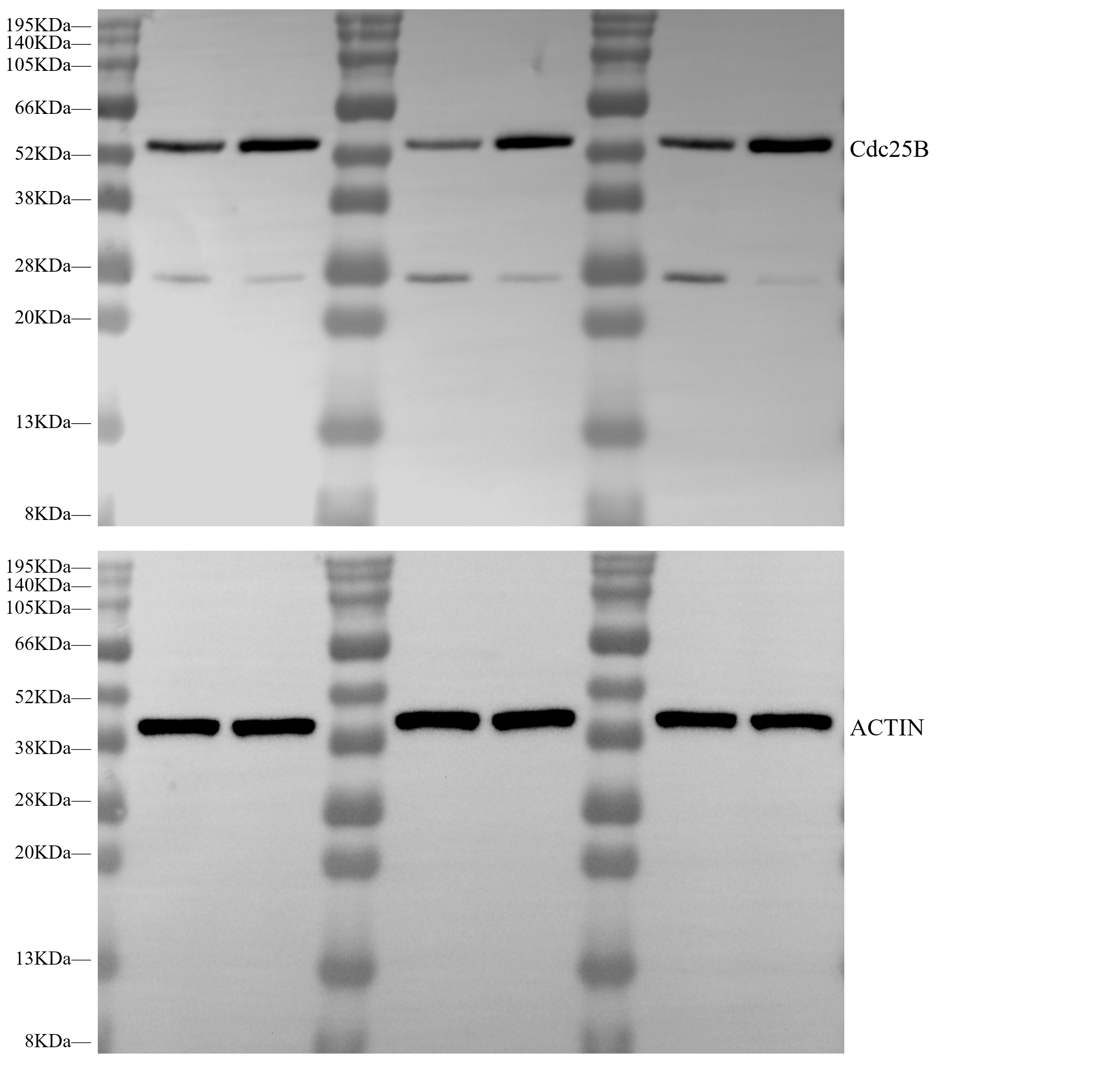

Supplement: Supplementary file 2 — Appendix S1. [file CNS-31-e70172-s001.zip › Supplementary File 1/3_WB/Traditional WB/CDC25B_WB.tif]

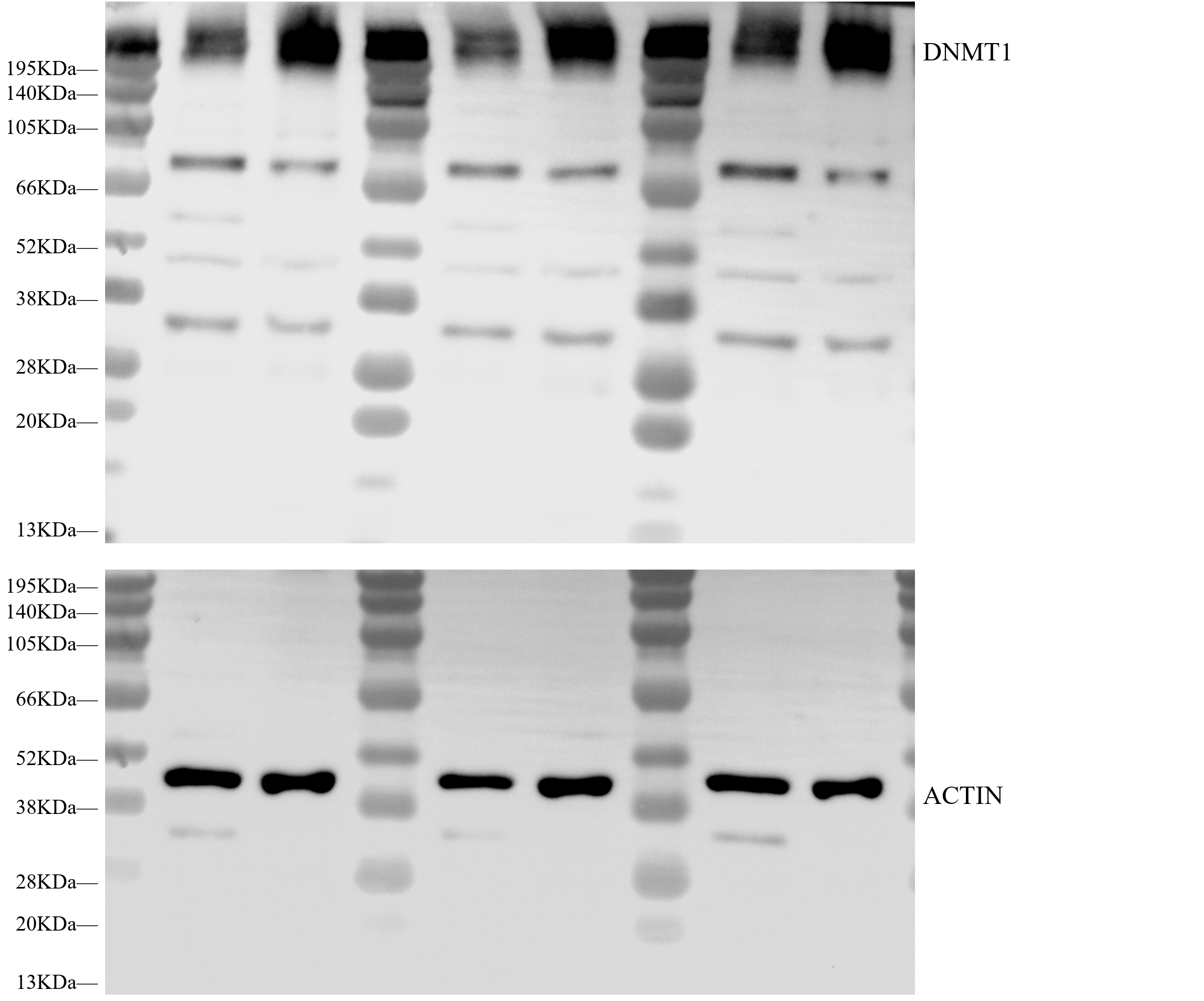

Supplement: Supplementary file 2 — Appendix S1. [file CNS-31-e70172-s001.zip › Supplementary File 1/3_WB/Traditional WB/DNMT1_WB.tif]

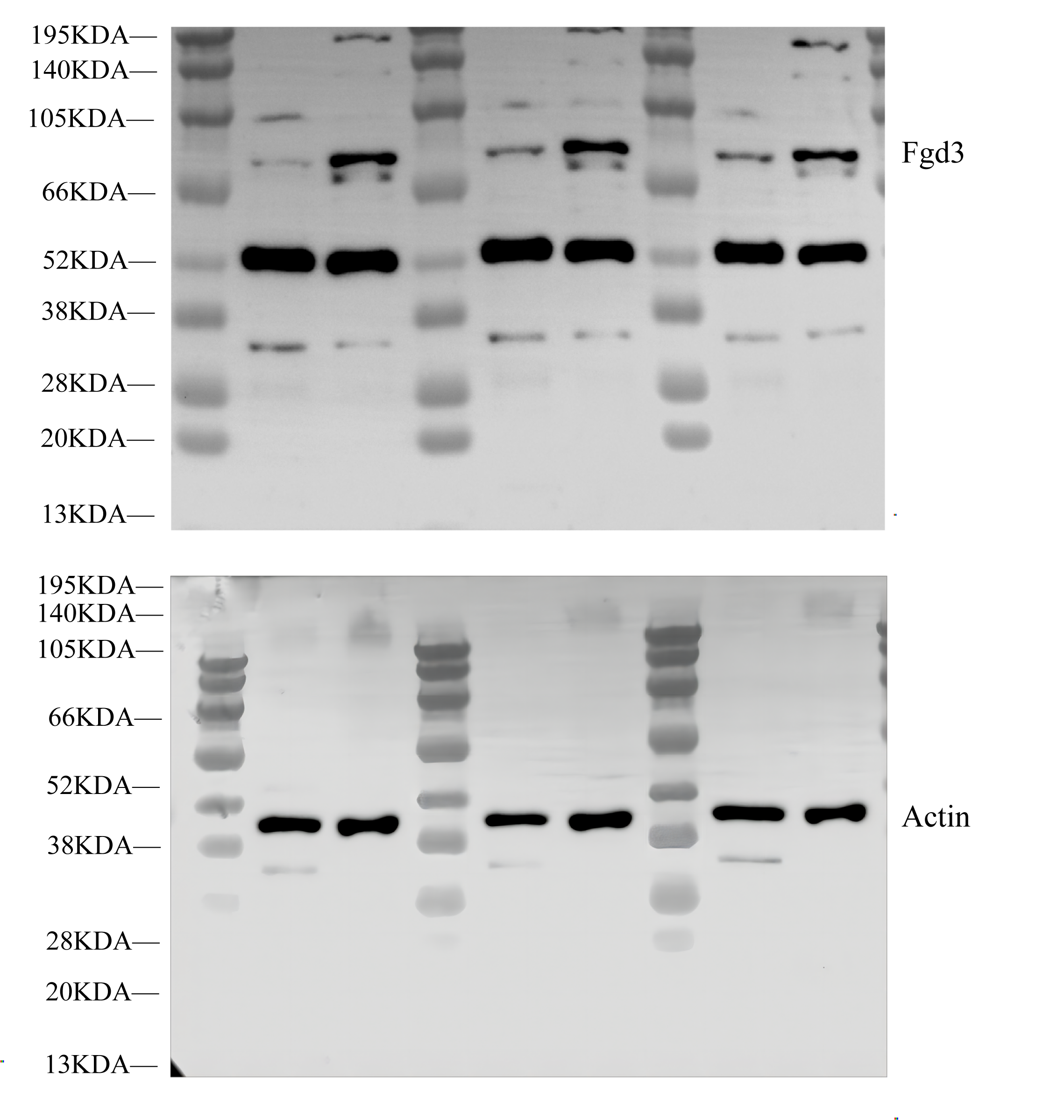

Supplement: Supplementary file 2 — Appendix S1. [file CNS-31-e70172-s001.zip › Supplementary File 1/3_WB/Traditional WB/FGD3_WB.tif]

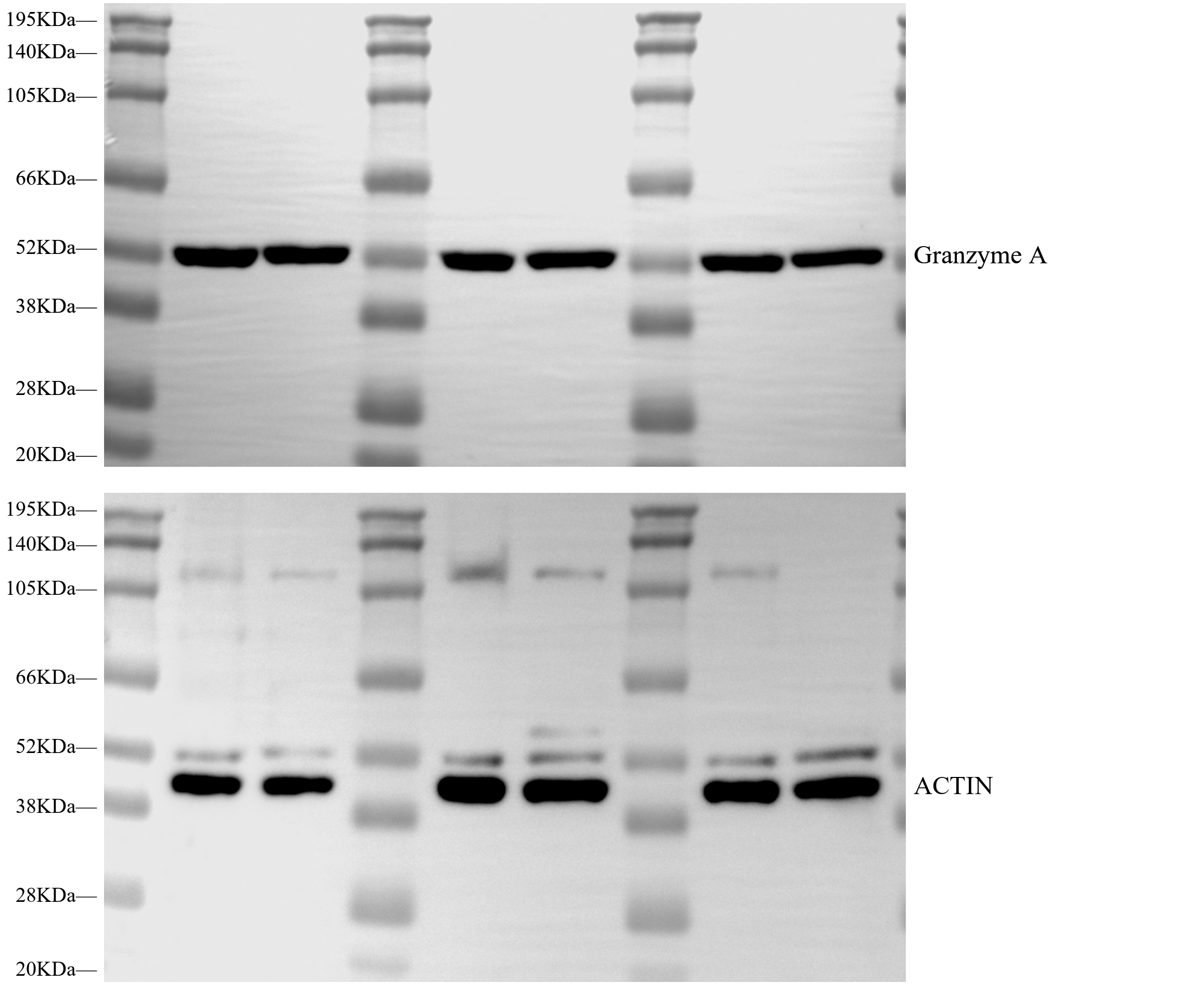

Supplement: Supplementary file 2 — Appendix S1. [file CNS-31-e70172-s001.zip › Supplementary File 1/3_WB/Traditional WB/GZMA_WB.tif]

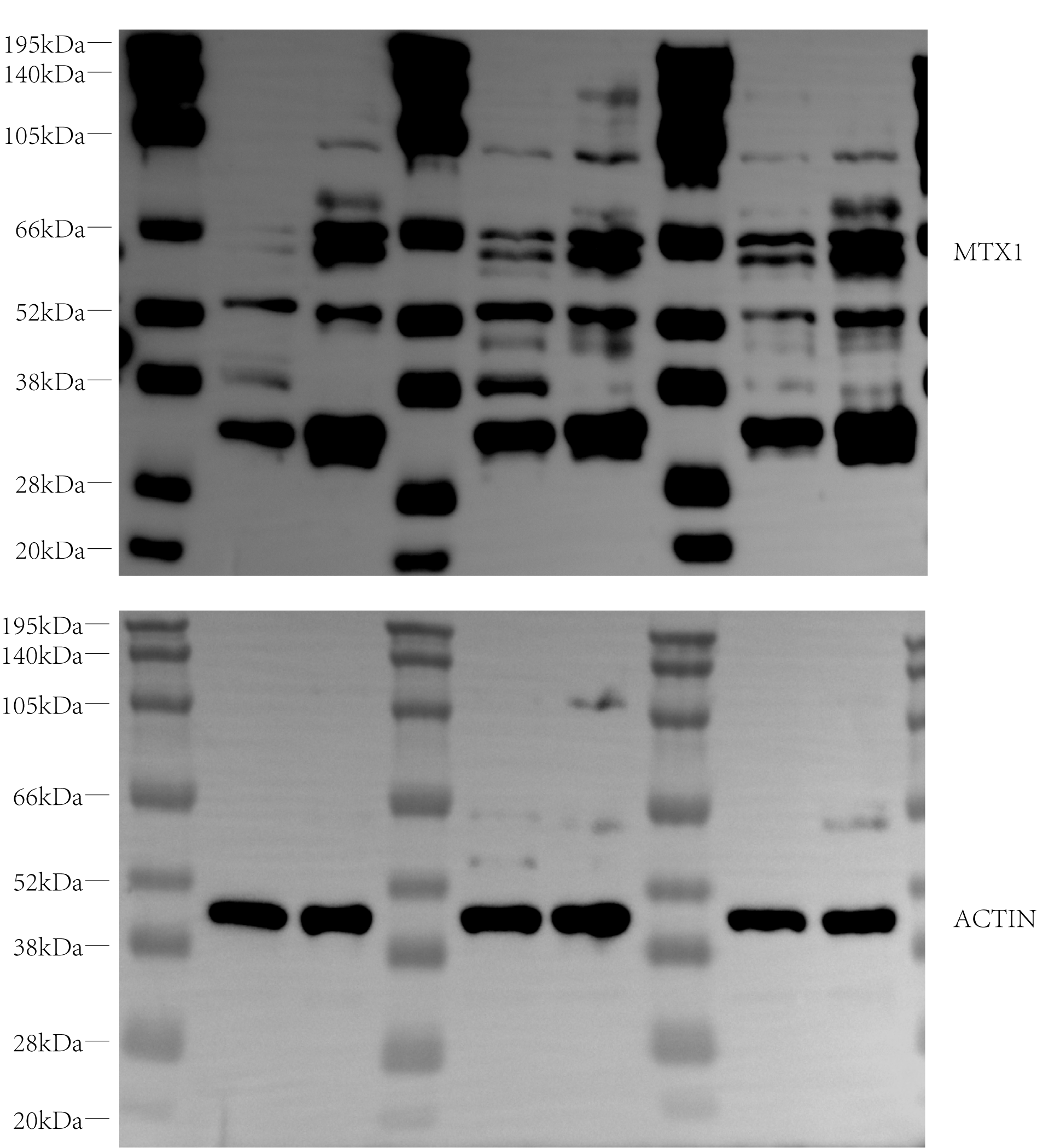

Supplement: Supplementary file 2 — Appendix S1. [file CNS-31-e70172-s001.zip › Supplementary File 1/3_WB/Traditional WB/MTX1_WB.tif]

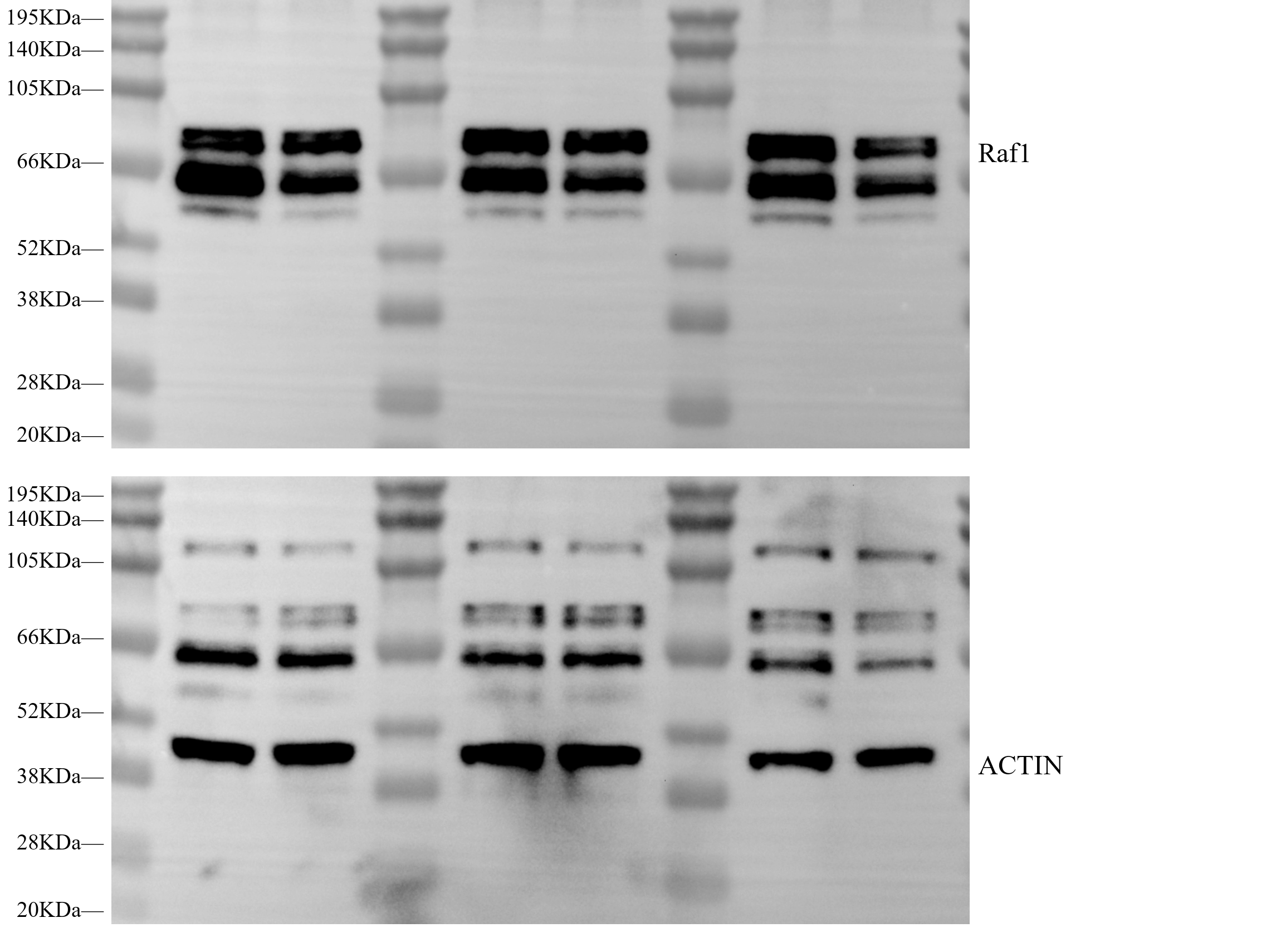

Supplement: Supplementary file 2 — Appendix S1. [file CNS-31-e70172-s001.zip › Supplementary File 1/3_WB/Traditional WB/RAF1_WB.tif]

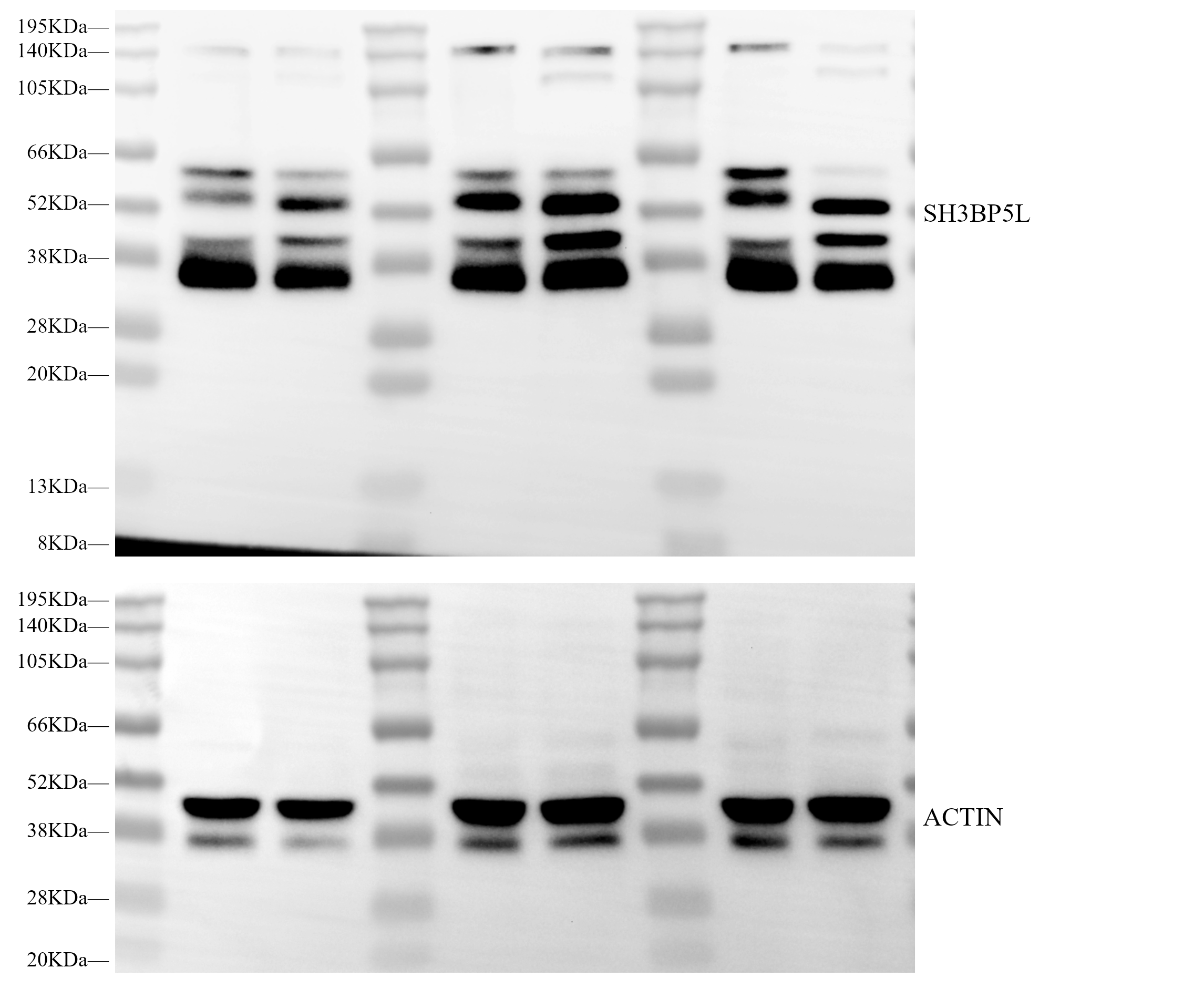

Supplement: Supplementary file 2 — Appendix S1. [file CNS-31-e70172-s001.zip › Supplementary File 1/3_WB/Traditional WB/SH3BP5L_WB.tif]

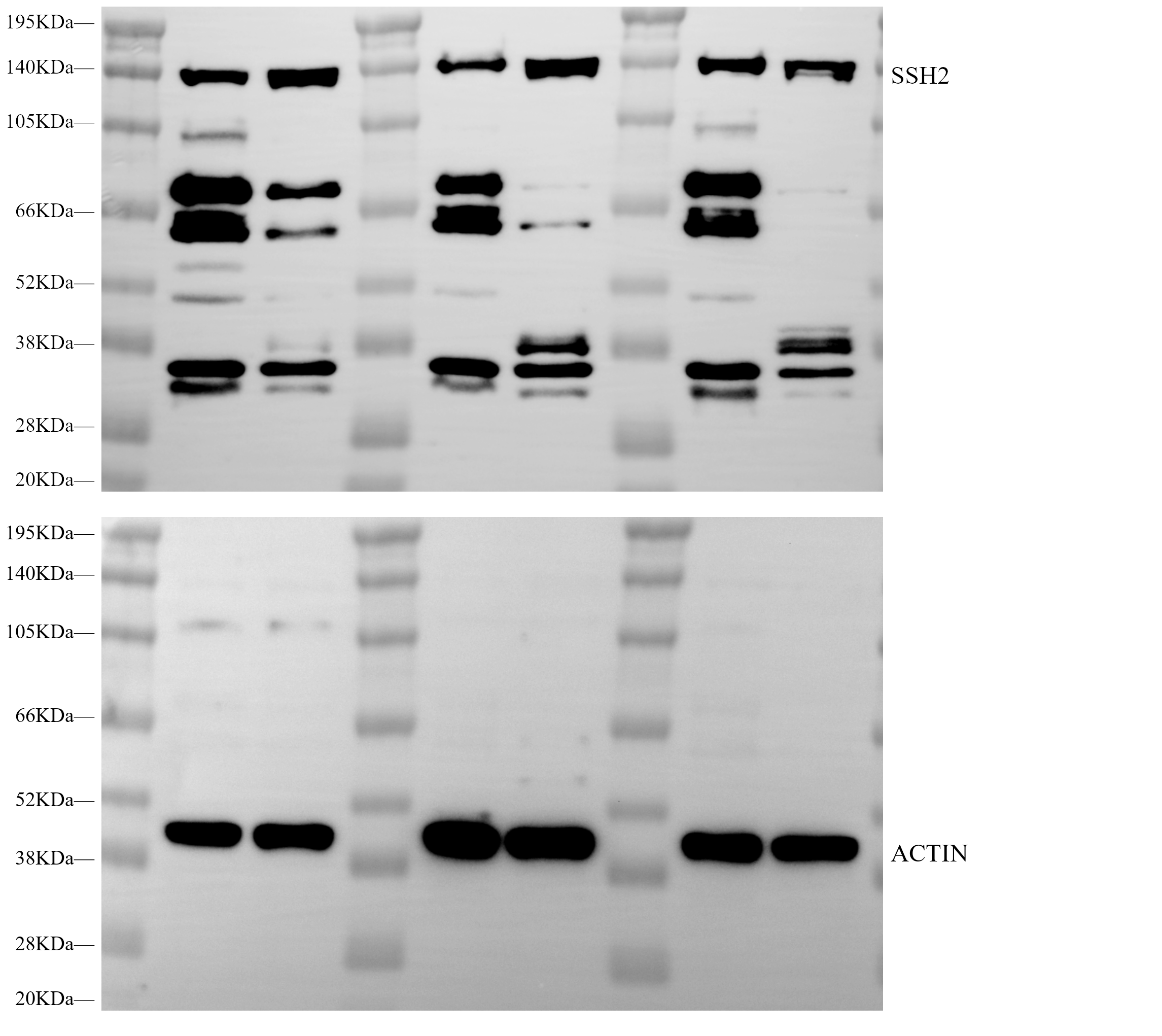

Supplement: Supplementary file 2 — Appendix S1. [file CNS-31-e70172-s001.zip › Supplementary File 1/3_WB/Traditional WB/SSH2_WB.tif]

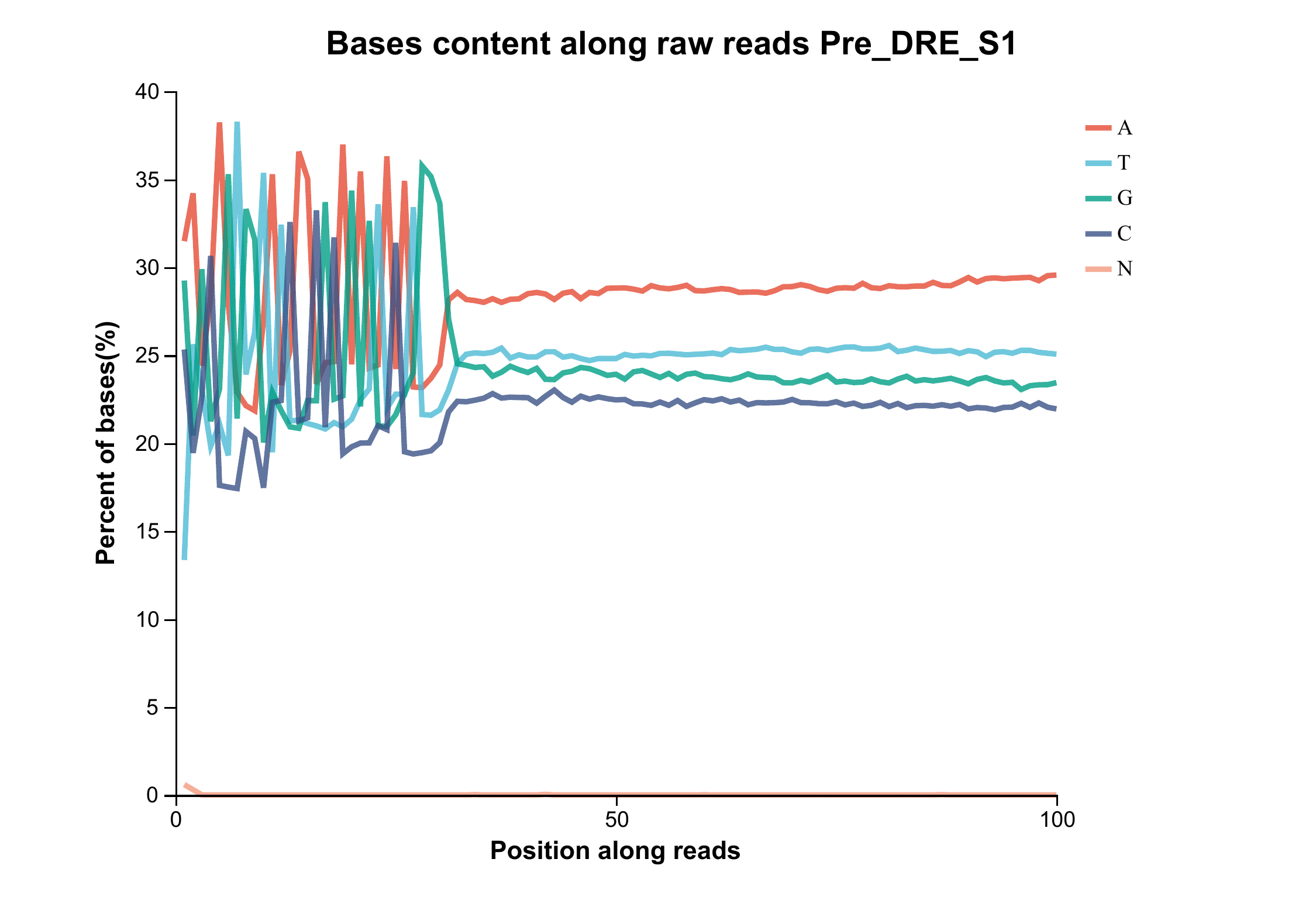

Supplement: Supplementary file 3 — Appendix S2. [file CNS-31-e70172-s002.zip › Supplementary File 2/1_Single Cell_Raw data statistics and quality assessment/Base content distribution.png]

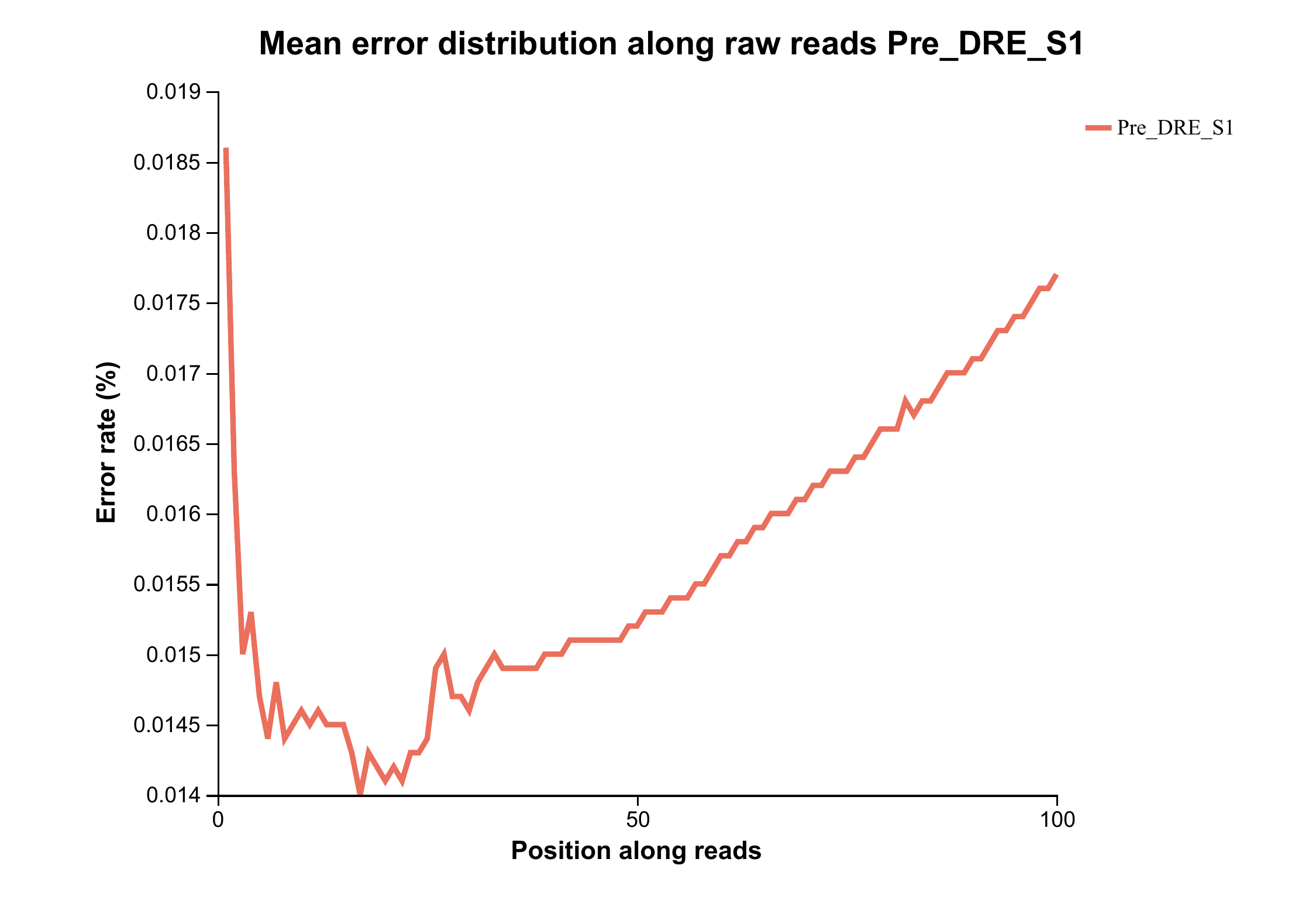

Supplement: Supplementary file 3 — Appendix S2. [file CNS-31-e70172-s002.zip › Supplementary File 2/1_Single Cell_Raw data statistics and quality assessment/Base error rate distribution.png]

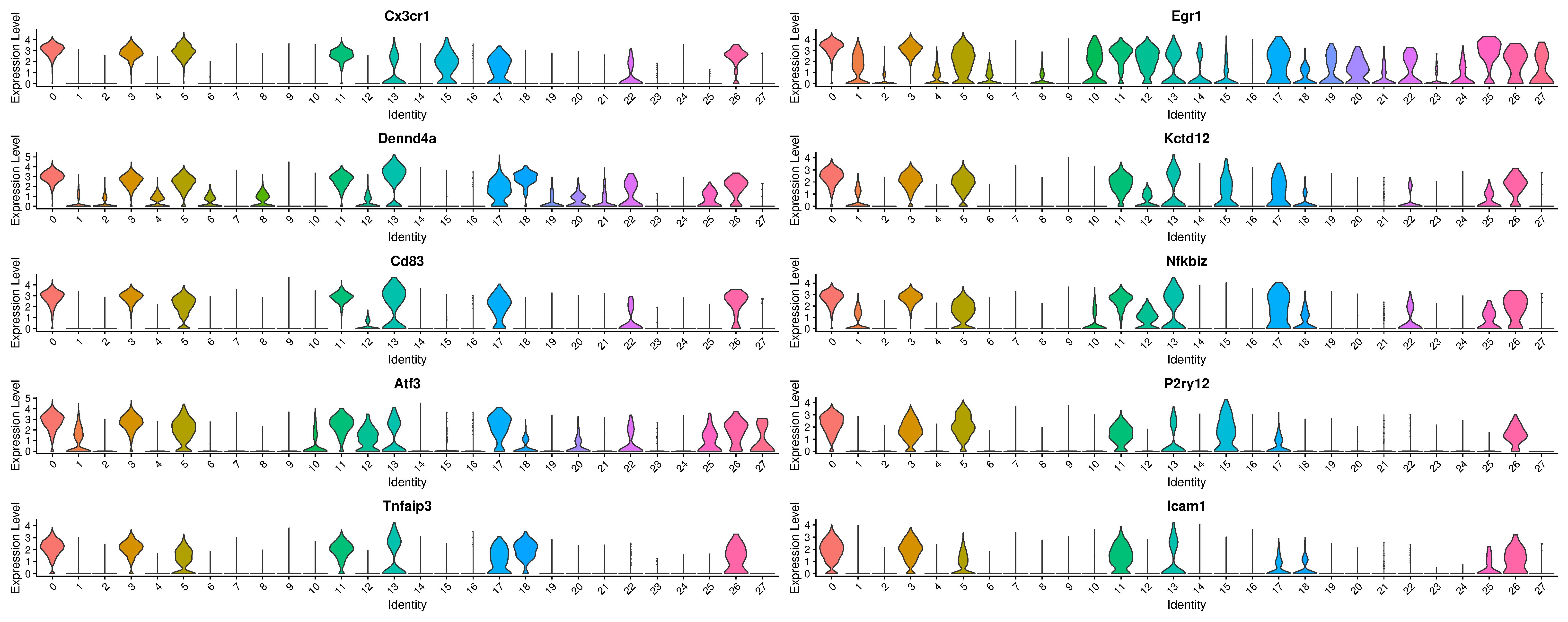

Supplement: Supplementary file 3 — Appendix S2. [file CNS-31-e70172-s002.zip › Supplementary File 2/2_Cluster marker top10 genes (28 clusters)/Cluster.0.VlnPlot_SC.png]

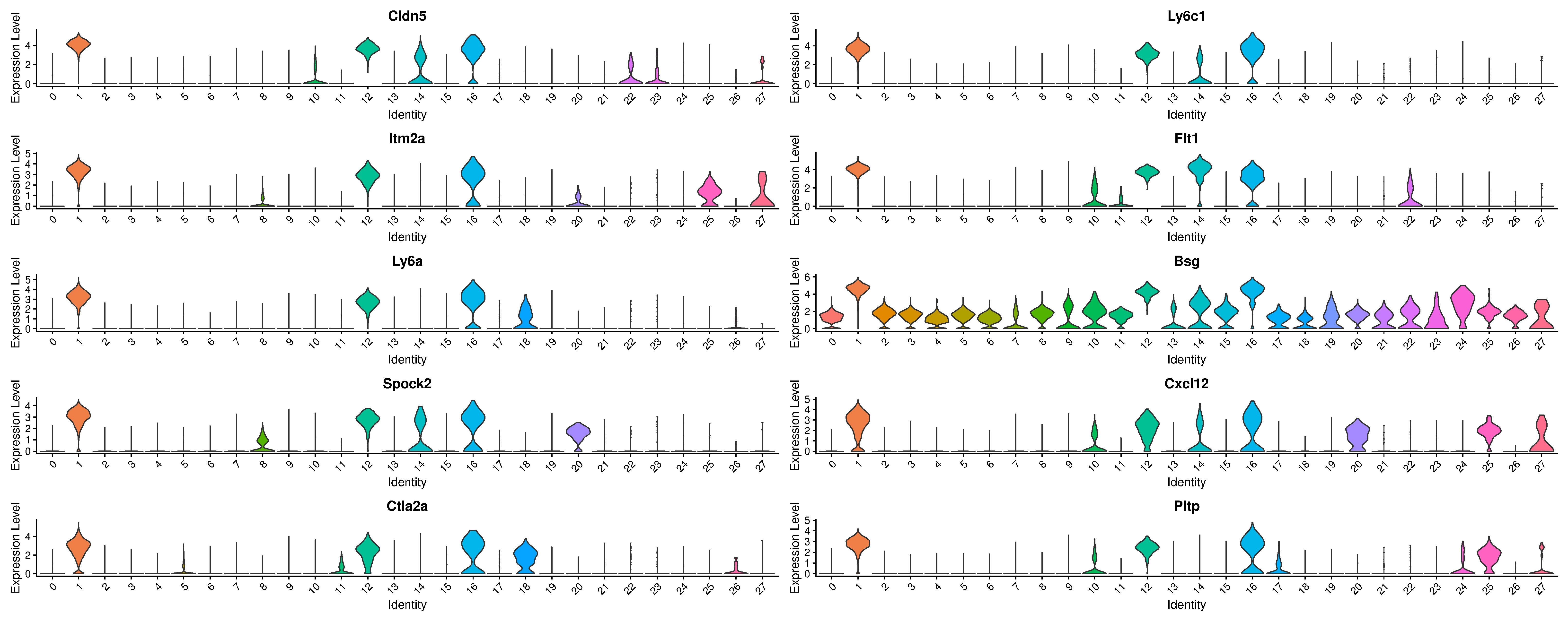

Supplement: Supplementary file 3 — Appendix S2. [file CNS-31-e70172-s002.zip › Supplementary File 2/2_Cluster marker top10 genes (28 clusters)/Cluster.1.VlnPlot_SC.png]

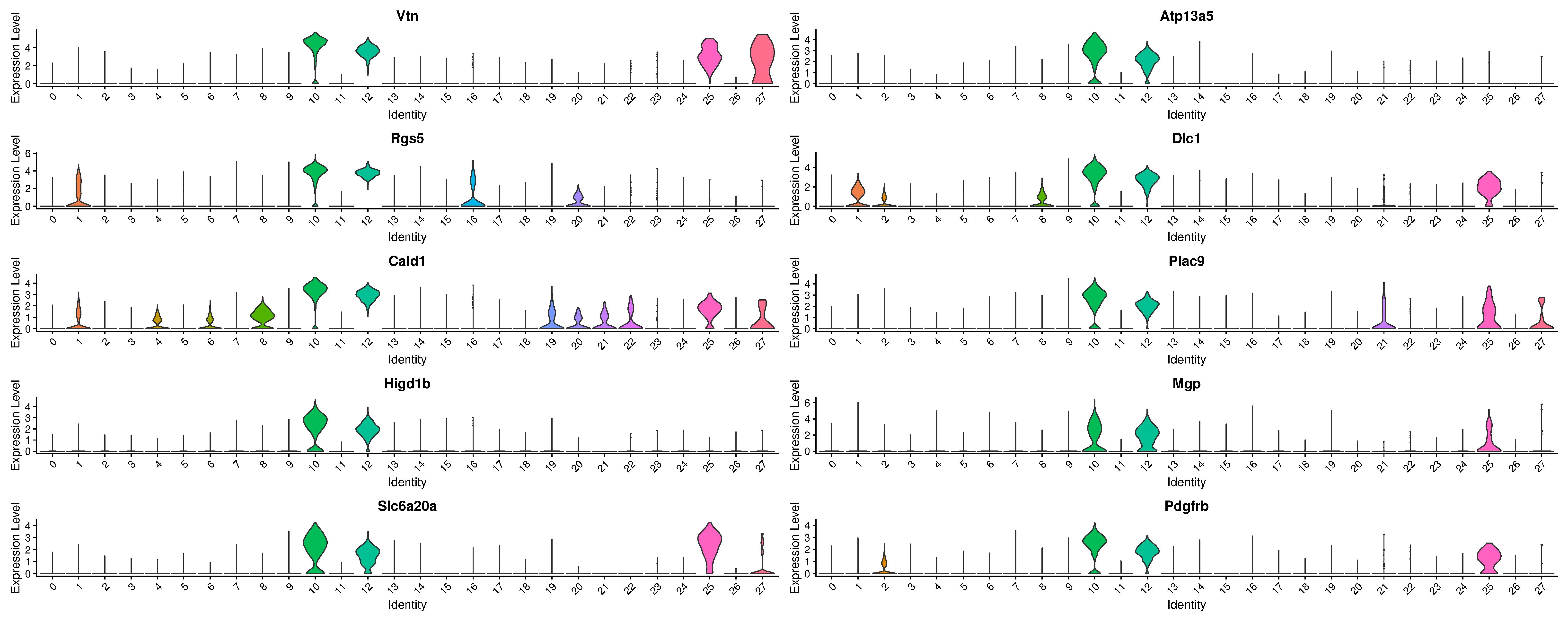

Supplement: Supplementary file 3 — Appendix S2. [file CNS-31-e70172-s002.zip › Supplementary File 2/2_Cluster marker top10 genes (28 clusters)/Cluster.10.VlnPlot_SC.png]

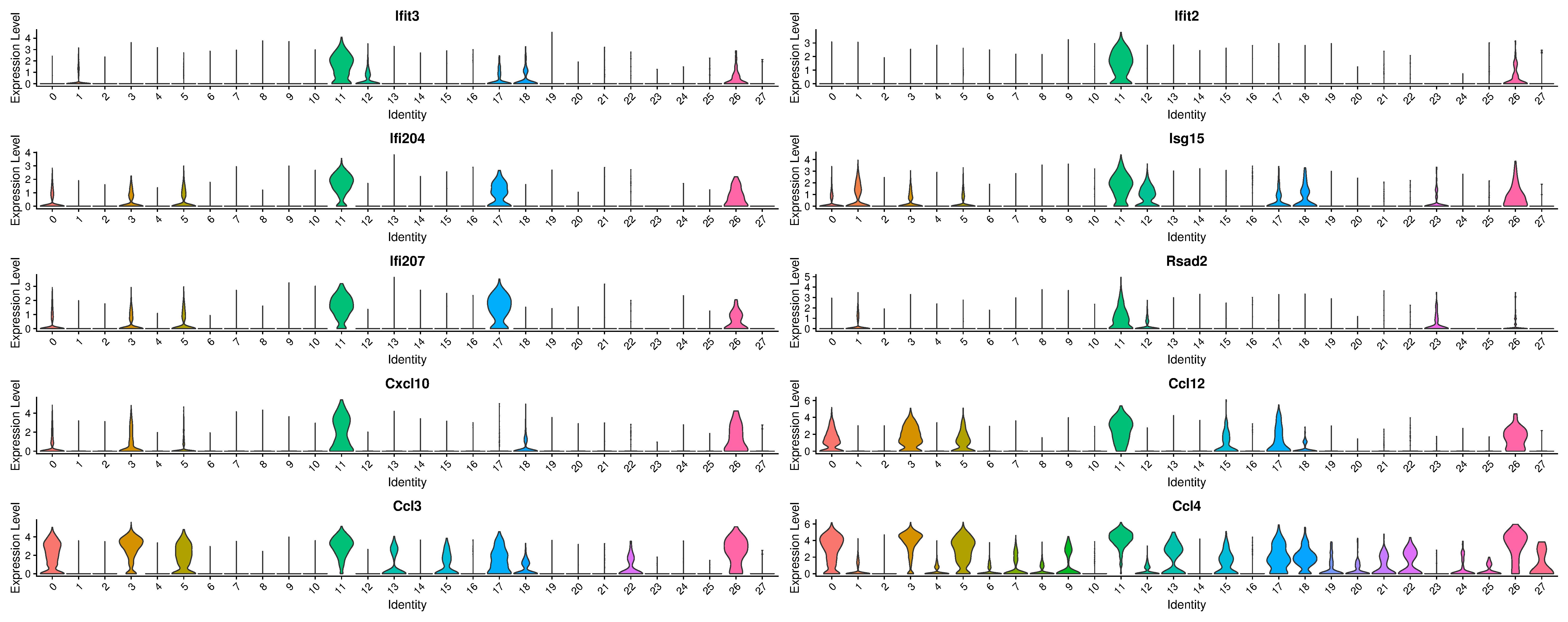

Supplement: Supplementary file 3 — Appendix S2. [file CNS-31-e70172-s002.zip › Supplementary File 2/2_Cluster marker top10 genes (28 clusters)/Cluster.11.VlnPlot_SC.png]

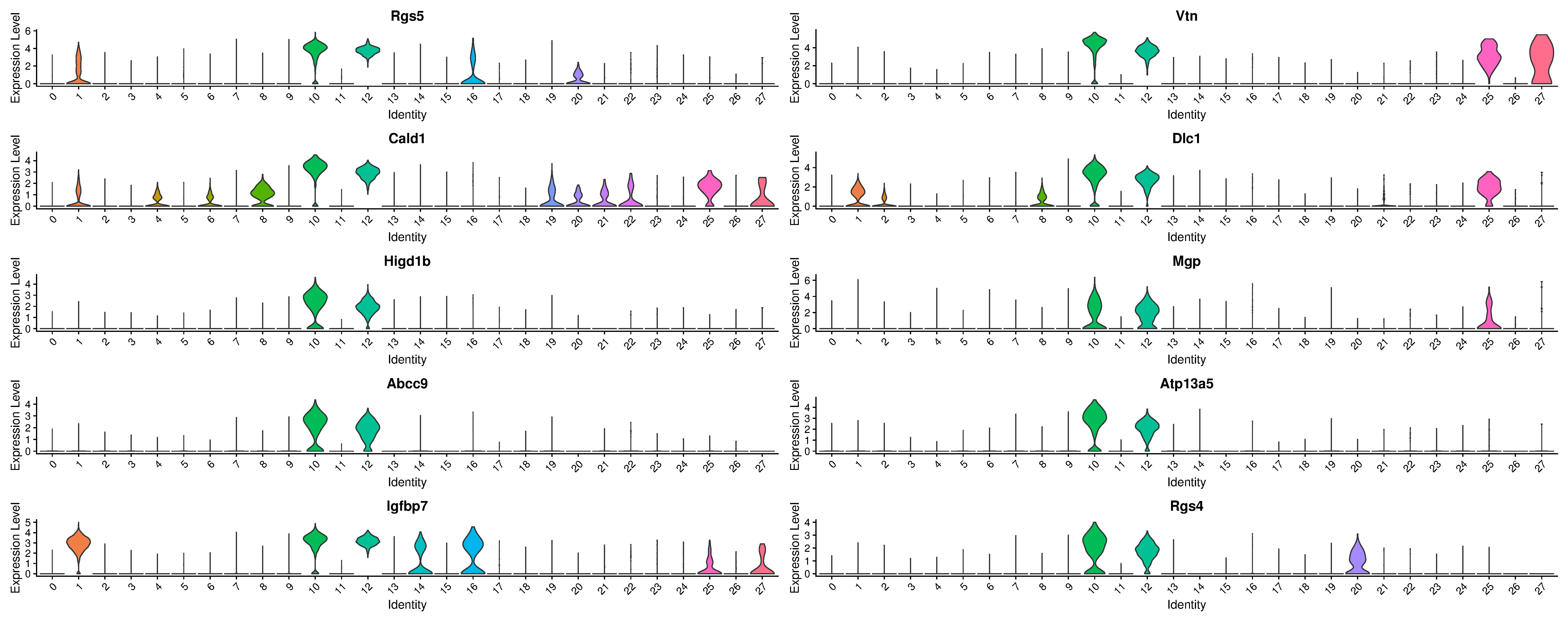

Supplement: Supplementary file 3 — Appendix S2. [file CNS-31-e70172-s002.zip › Supplementary File 2/2_Cluster marker top10 genes (28 clusters)/Cluster.12.VlnPlot_SC.png]

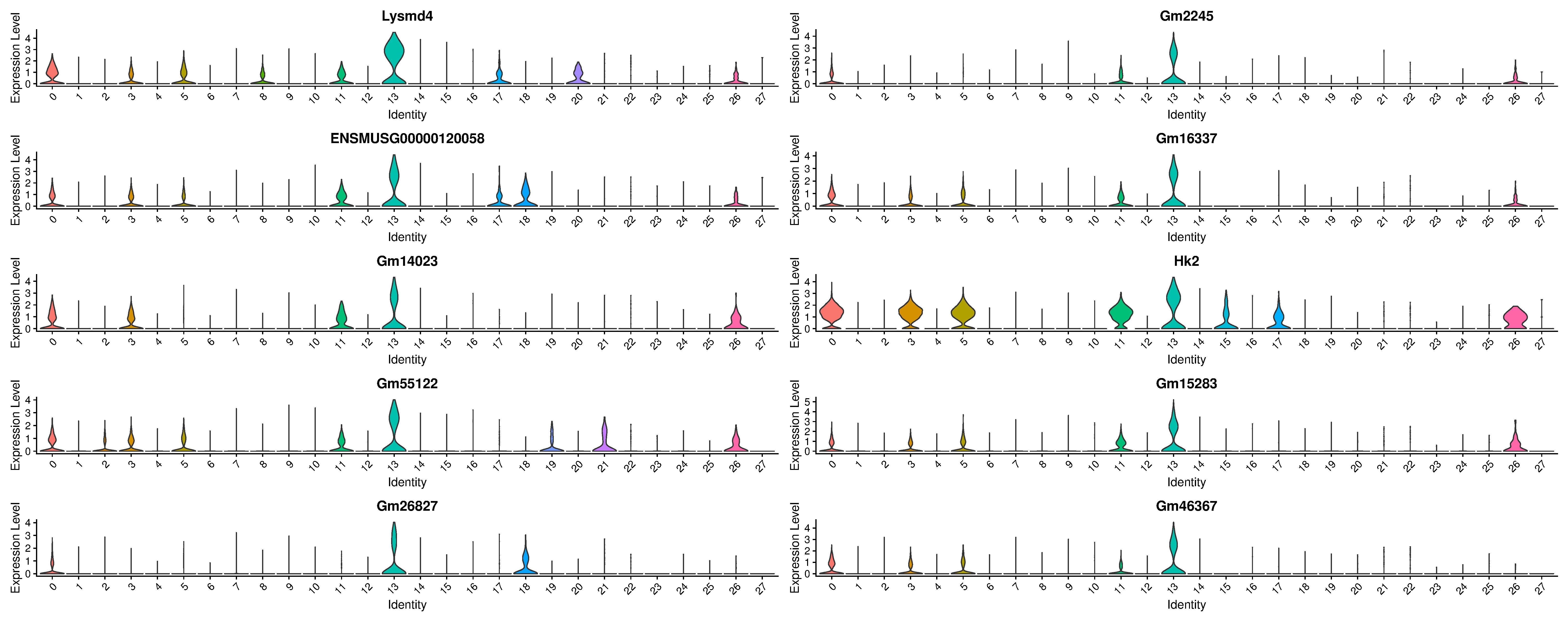

Supplement: Supplementary file 3 — Appendix S2. [file CNS-31-e70172-s002.zip › Supplementary File 2/2_Cluster marker top10 genes (28 clusters)/Cluster.13.VlnPlot_SC.png]

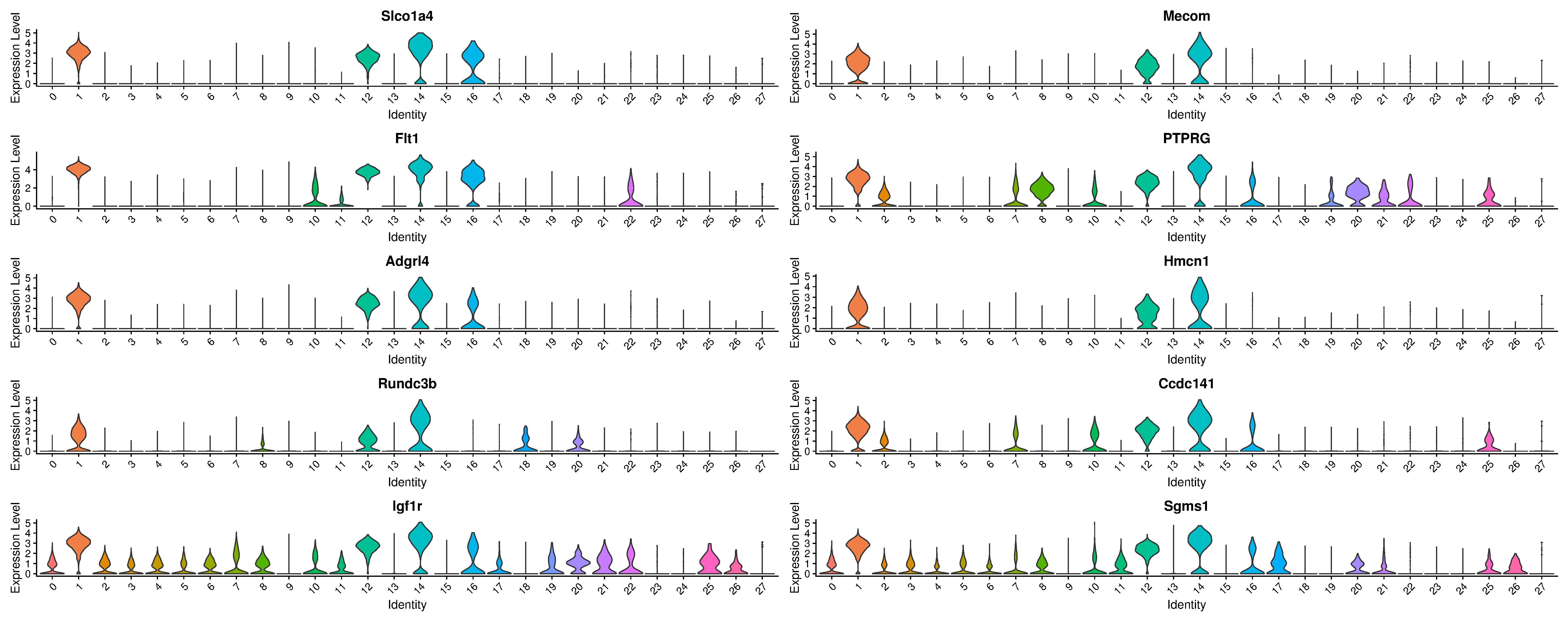

Supplement: Supplementary file 3 — Appendix S2. [file CNS-31-e70172-s002.zip › Supplementary File 2/2_Cluster marker top10 genes (28 clusters)/Cluster.14.VlnPlot_SC.png]

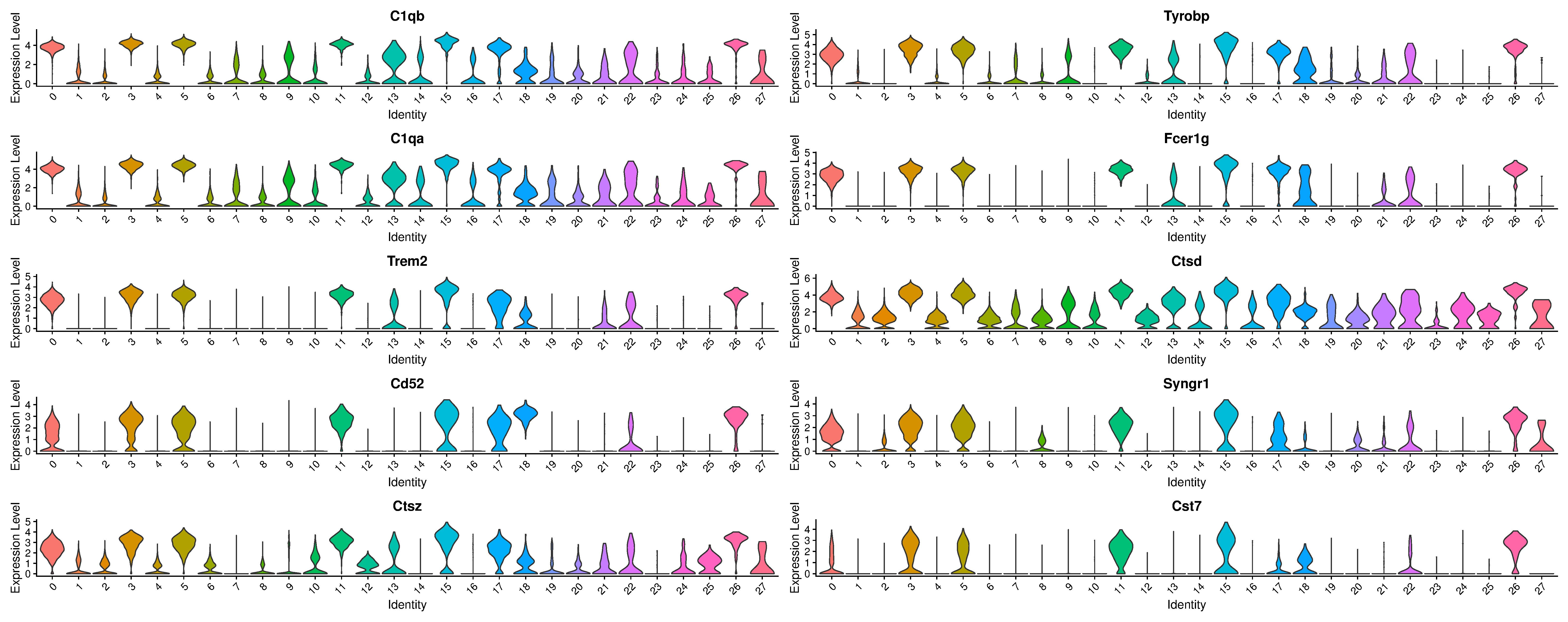

Supplement: Supplementary file 3 — Appendix S2. [file CNS-31-e70172-s002.zip › Supplementary File 2/2_Cluster marker top10 genes (28 clusters)/Cluster.15.VlnPlot_SC.png]

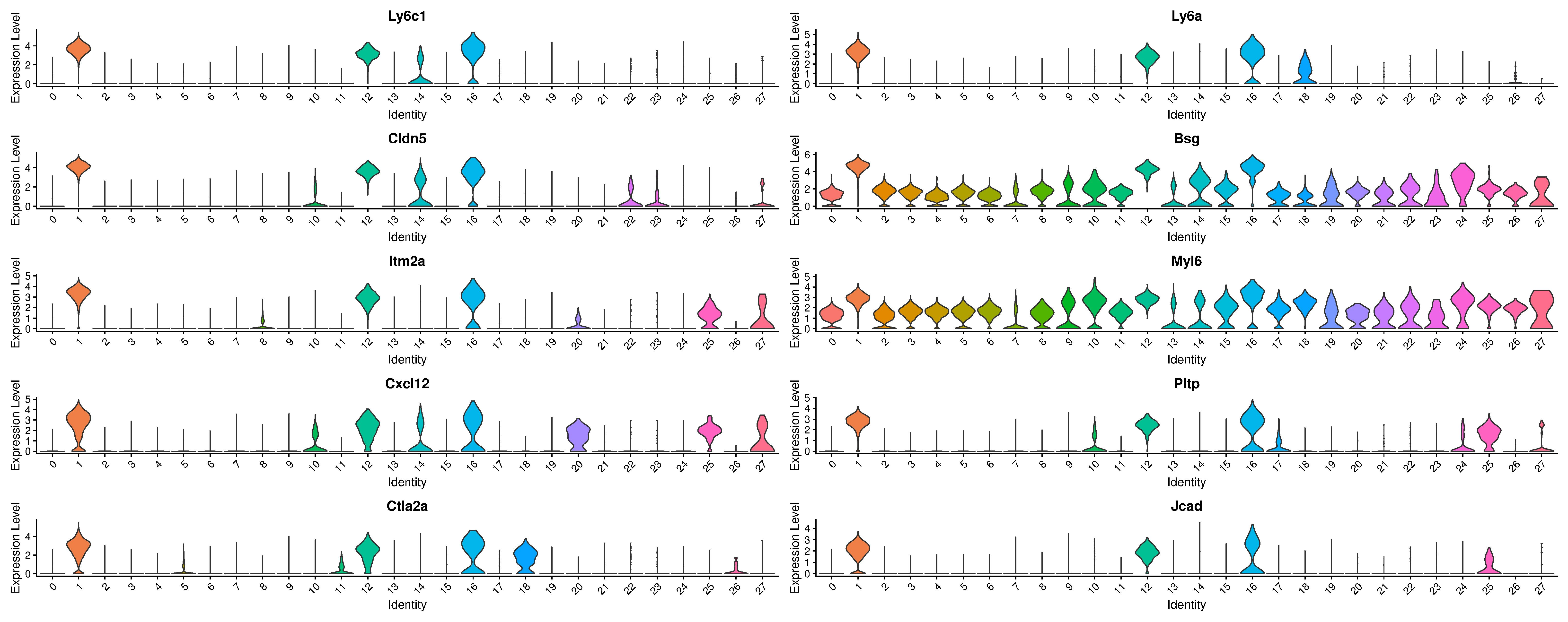

Supplement: Supplementary file 3 — Appendix S2. [file CNS-31-e70172-s002.zip › Supplementary File 2/2_Cluster marker top10 genes (28 clusters)/Cluster.16.VlnPlot_SC.png]

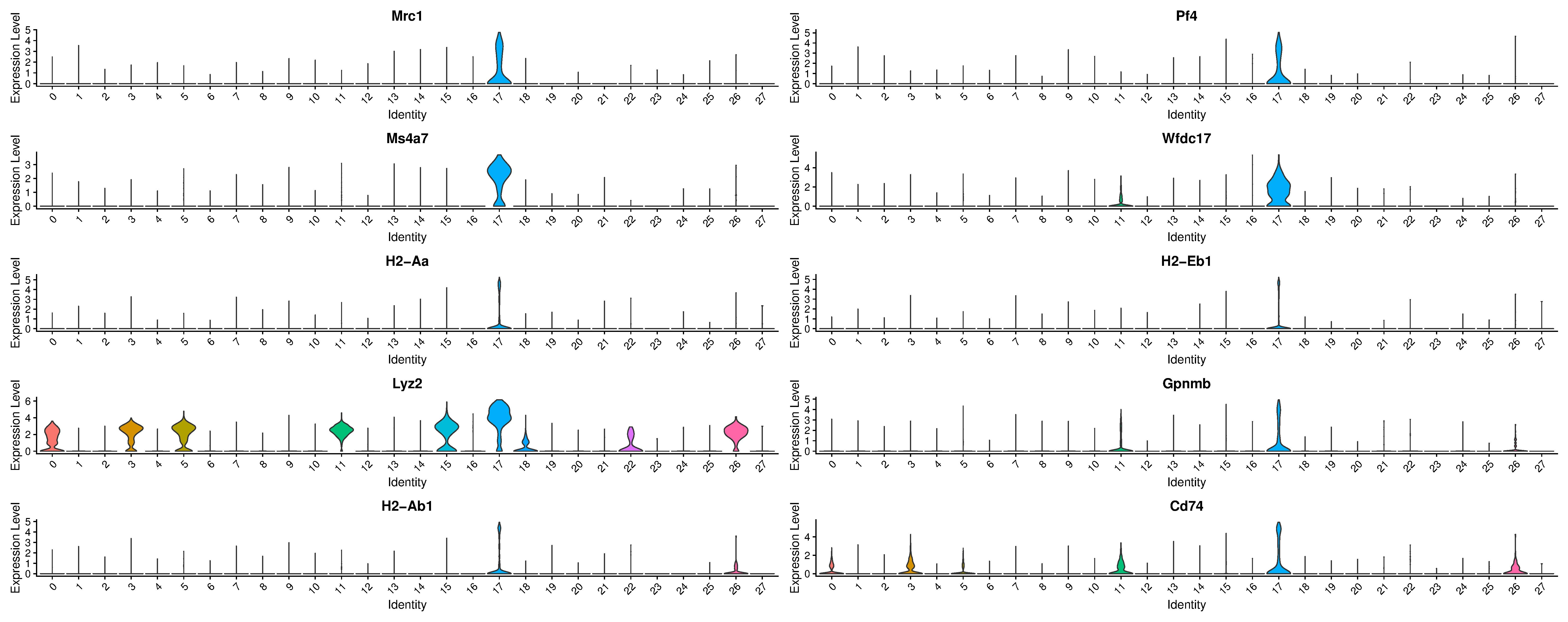

Supplement: Supplementary file 3 — Appendix S2. [file CNS-31-e70172-s002.zip › Supplementary File 2/2_Cluster marker top10 genes (28 clusters)/Cluster.17.VlnPlot_SC.png]

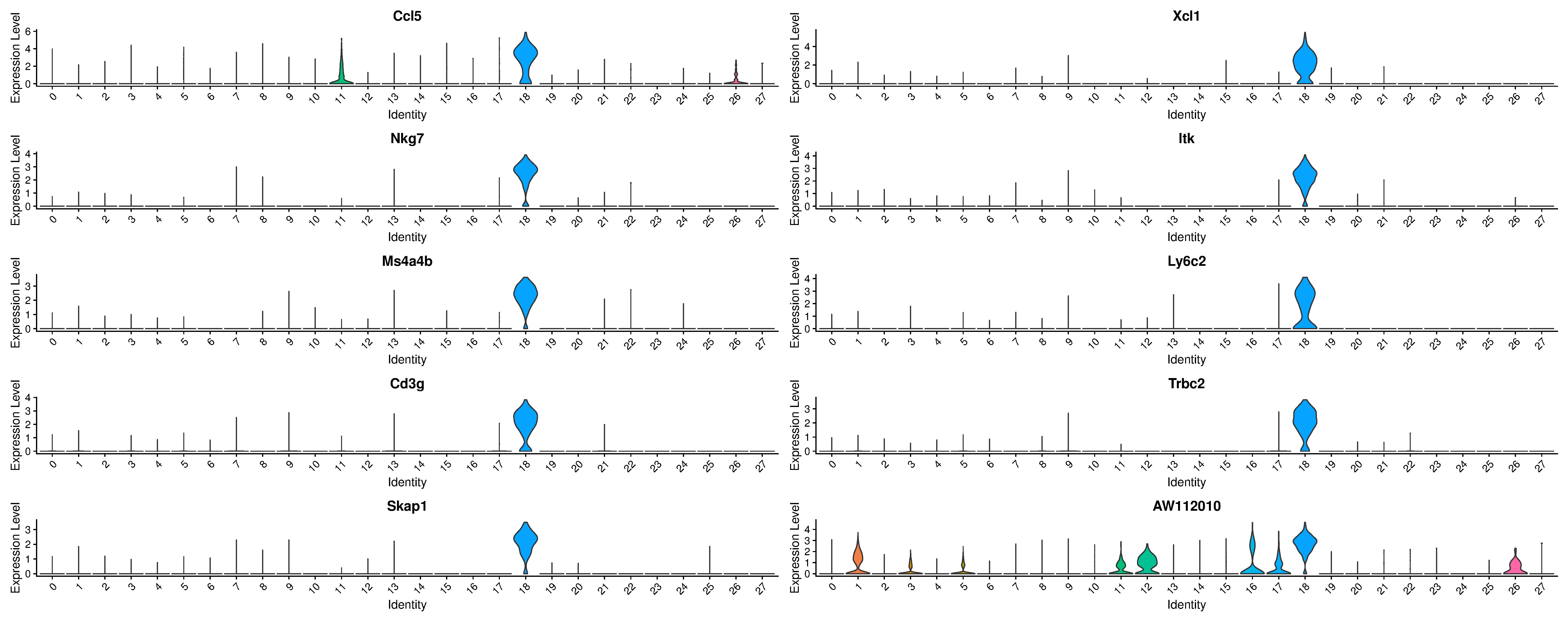

Supplement: Supplementary file 3 — Appendix S2. [file CNS-31-e70172-s002.zip › Supplementary File 2/2_Cluster marker top10 genes (28 clusters)/Cluster.18.VlnPlot_SC.png]

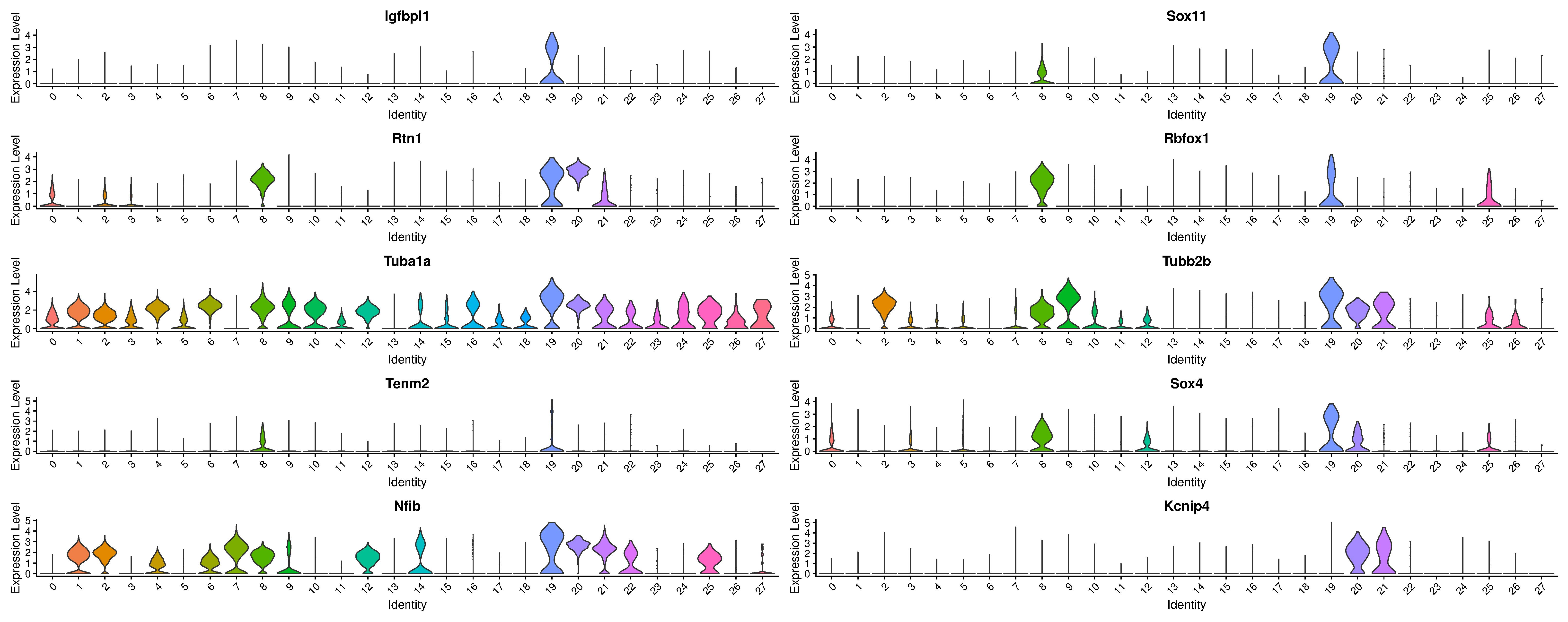

Supplement: Supplementary file 3 — Appendix S2. [file CNS-31-e70172-s002.zip › Supplementary File 2/2_Cluster marker top10 genes (28 clusters)/Cluster.19.VlnPlot_SC.png]

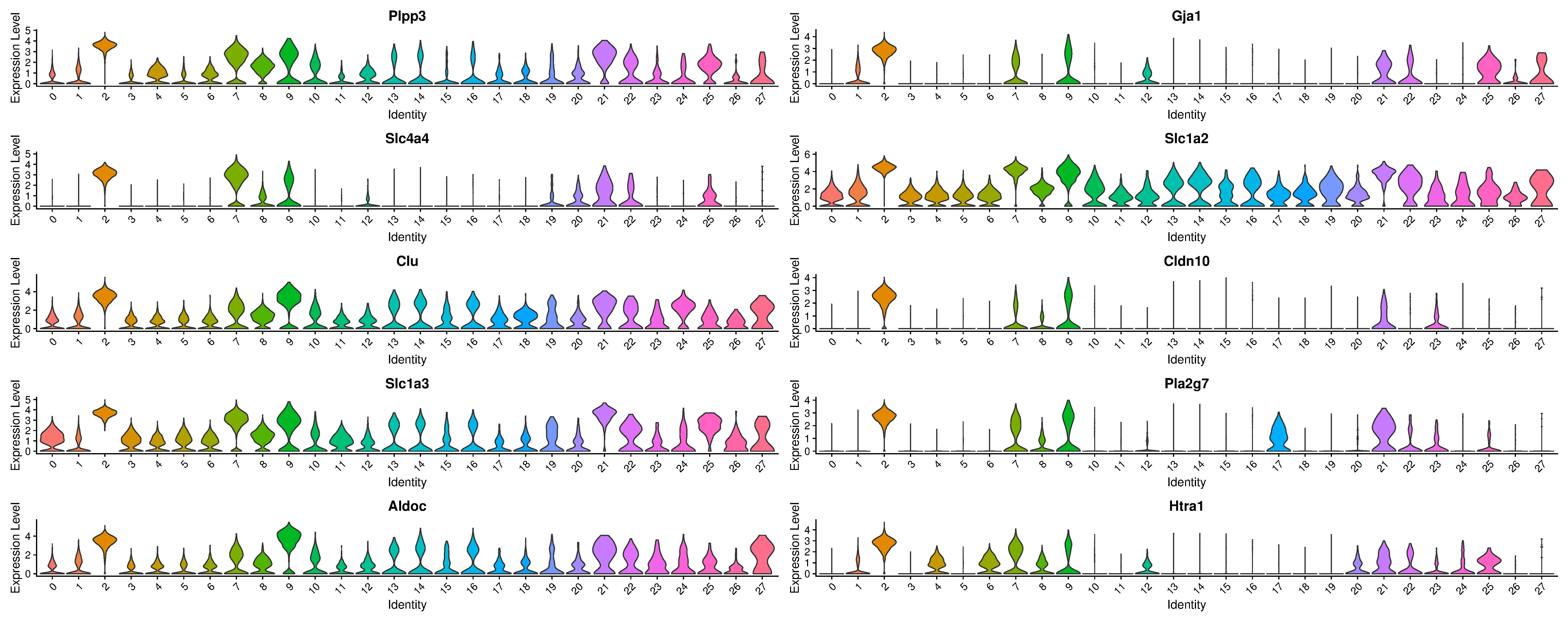

Supplement: Supplementary file 3 — Appendix S2. [file CNS-31-e70172-s002.zip › Supplementary File 2/2_Cluster marker top10 genes (28 clusters)/Cluster.2.VlnPlot_SC.png]

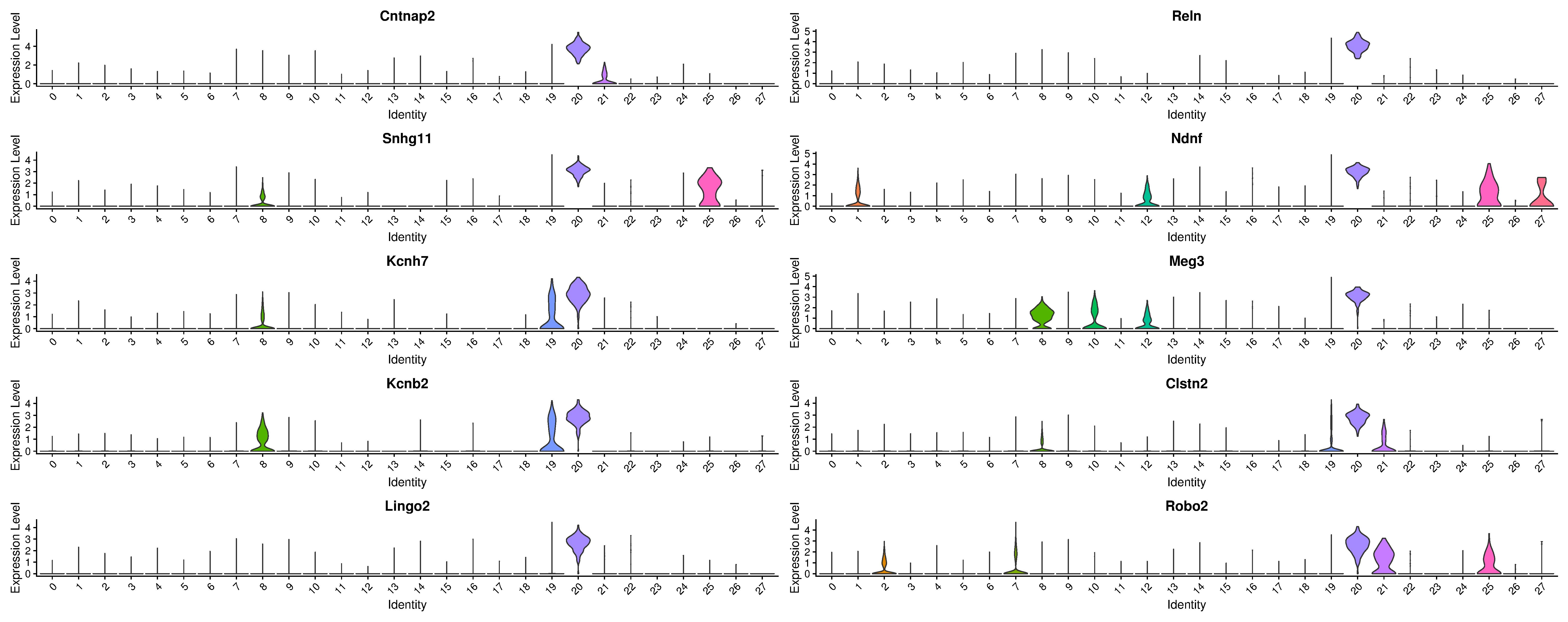

Supplement: Supplementary file 3 — Appendix S2. [file CNS-31-e70172-s002.zip › Supplementary File 2/2_Cluster marker top10 genes (28 clusters)/Cluster.20.VlnPlot_SC.png]

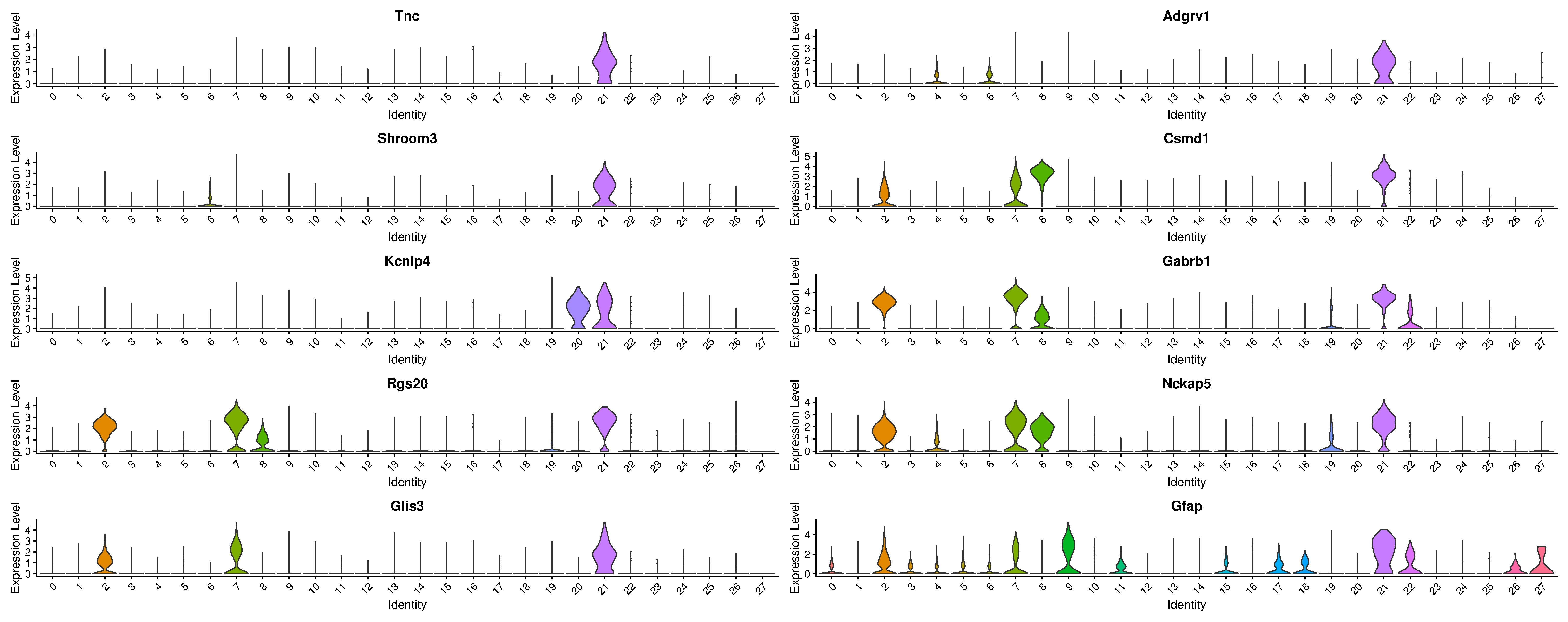

Supplement: Supplementary file 3 — Appendix S2. [file CNS-31-e70172-s002.zip › Supplementary File 2/2_Cluster marker top10 genes (28 clusters)/Cluster.21.VlnPlot_SC.png]

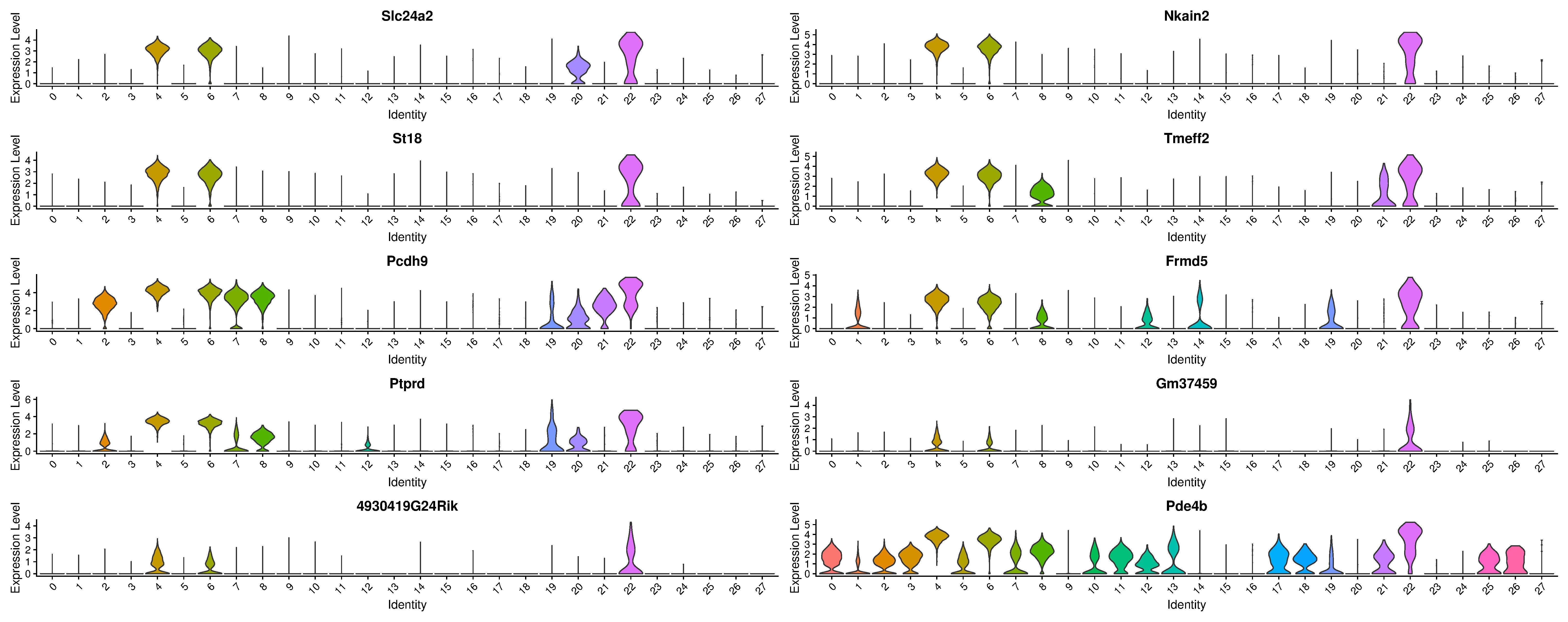

Supplement: Supplementary file 3 — Appendix S2. [file CNS-31-e70172-s002.zip › Supplementary File 2/2_Cluster marker top10 genes (28 clusters)/Cluster.22.VlnPlot_SC.png]

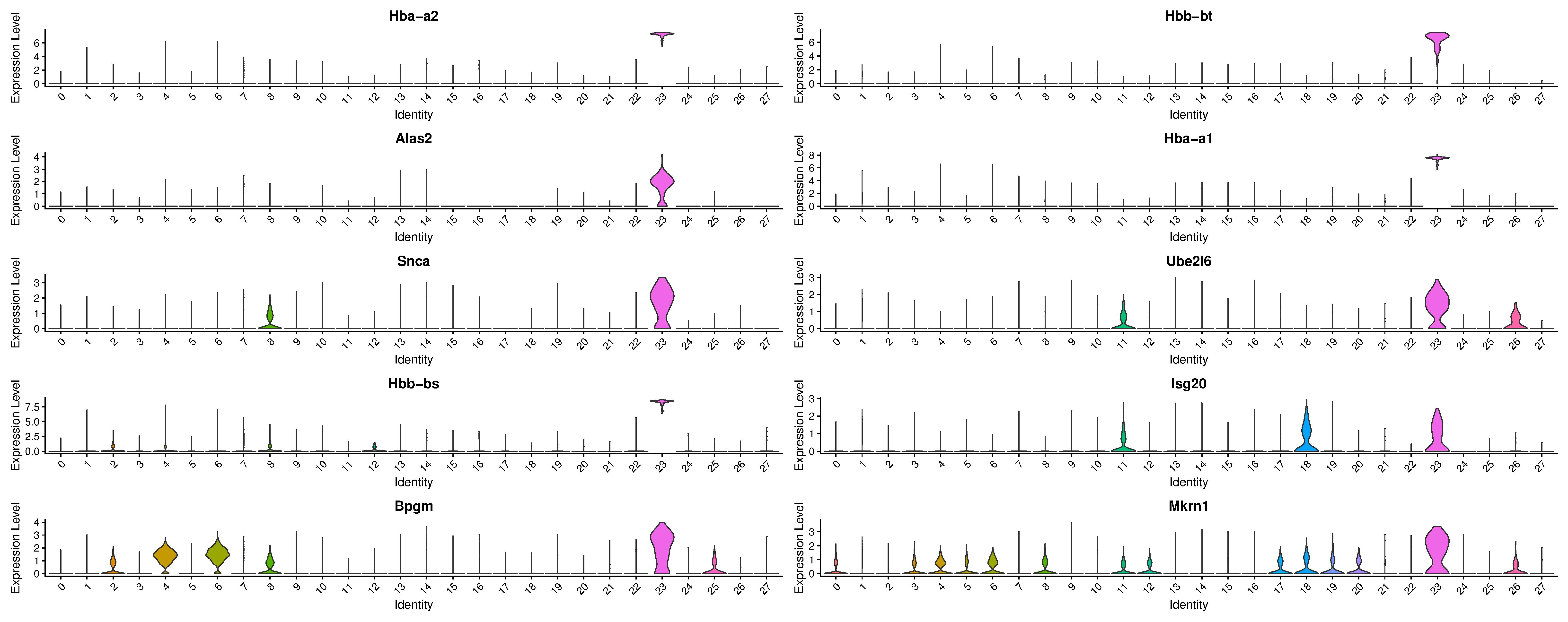

Supplement: Supplementary file 3 — Appendix S2. [file CNS-31-e70172-s002.zip › Supplementary File 2/2_Cluster marker top10 genes (28 clusters)/Cluster.23.VlnPlot_SC.png]

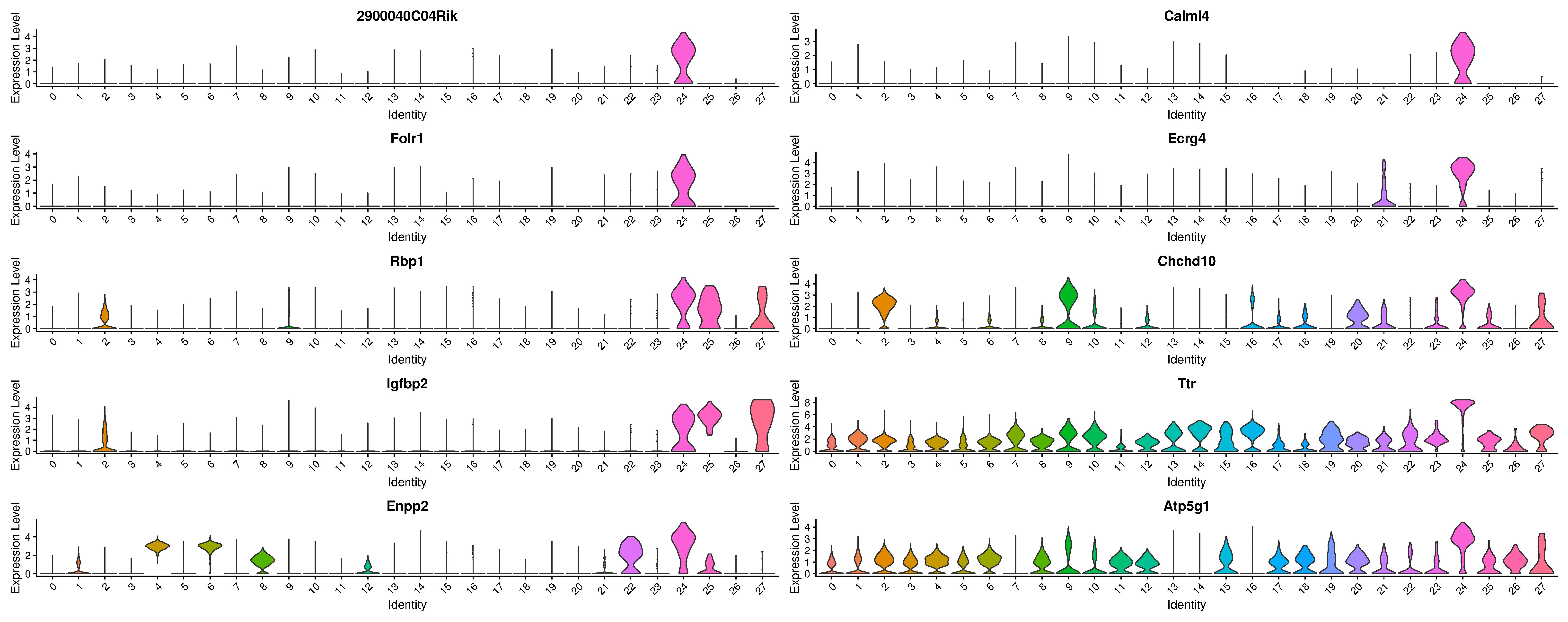

Supplement: Supplementary file 3 — Appendix S2. [file CNS-31-e70172-s002.zip › Supplementary File 2/2_Cluster marker top10 genes (28 clusters)/Cluster.24.VlnPlot_SC.png]

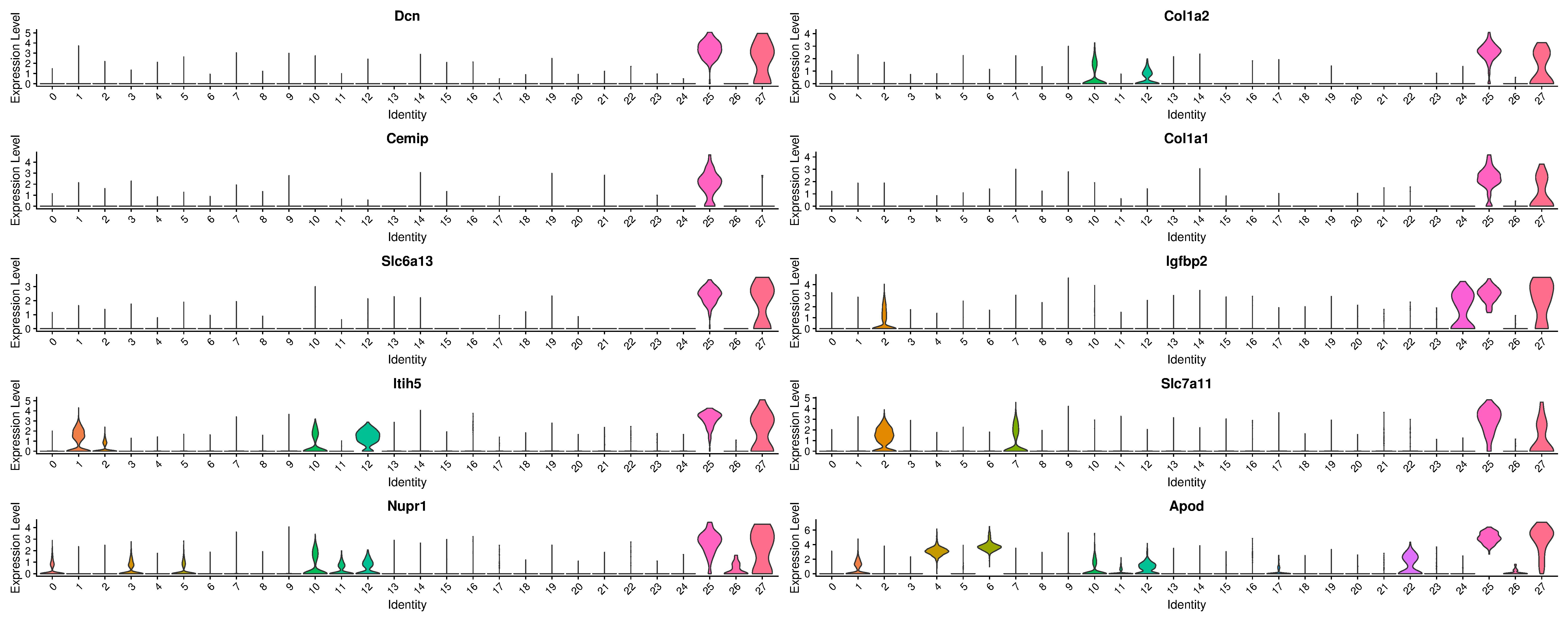

Supplement: Supplementary file 3 — Appendix S2. [file CNS-31-e70172-s002.zip › Supplementary File 2/2_Cluster marker top10 genes (28 clusters)/Cluster.25.VlnPlot_SC.png]

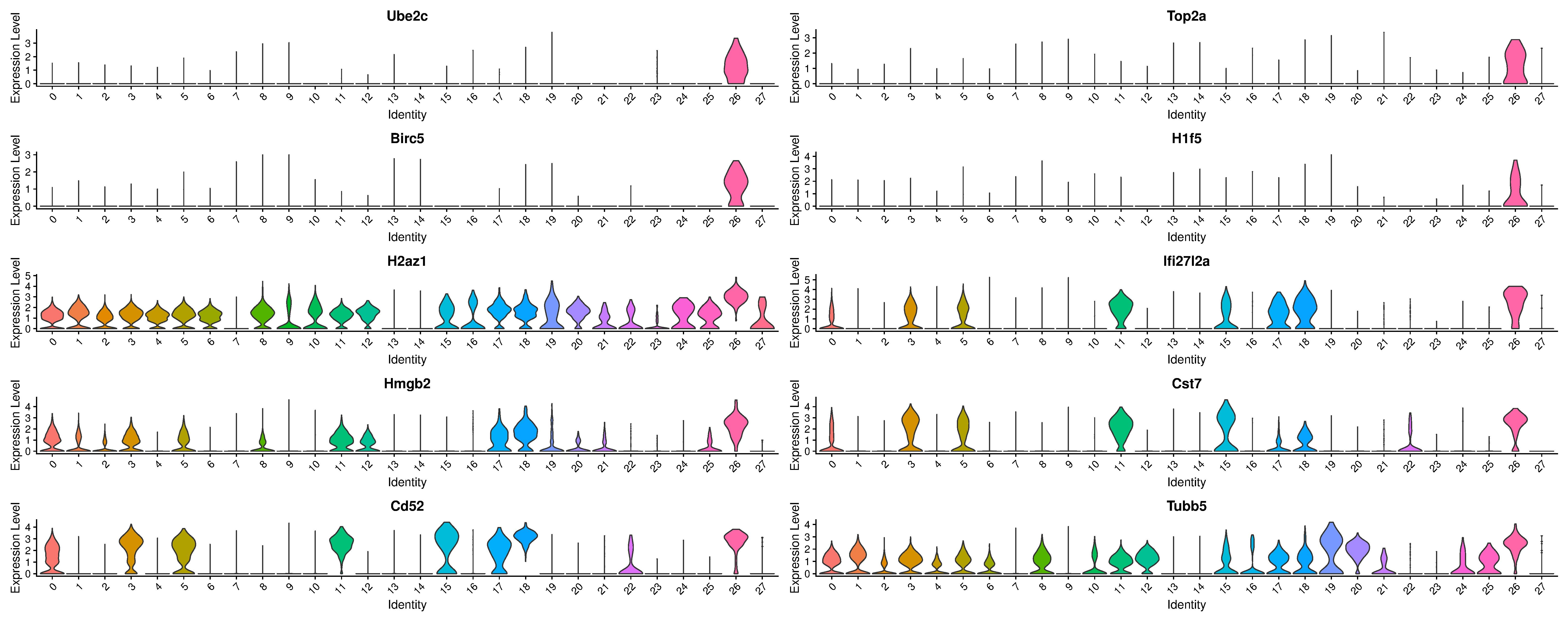

Supplement: Supplementary file 3 — Appendix S2. [file CNS-31-e70172-s002.zip › Supplementary File 2/2_Cluster marker top10 genes (28 clusters)/Cluster.26.VlnPlot_SC.png]

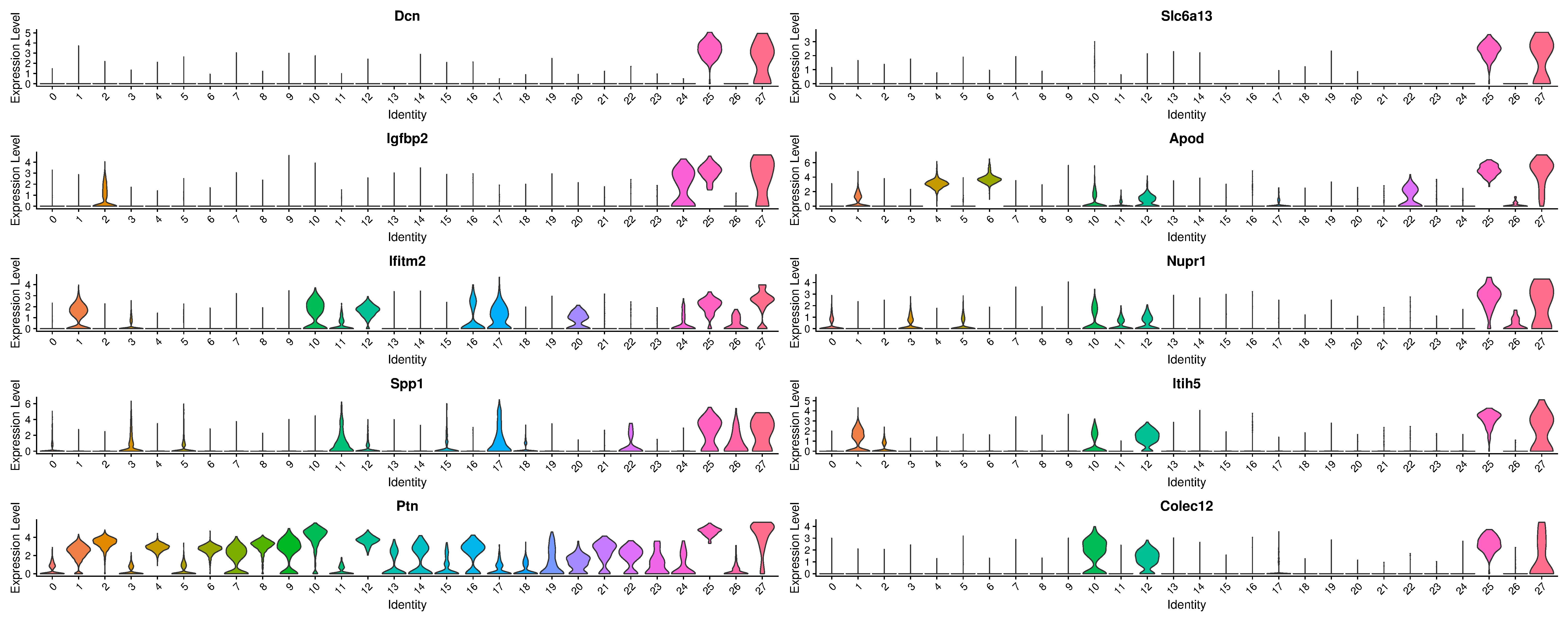

Supplement: Supplementary file 3 — Appendix S2. [file CNS-31-e70172-s002.zip › Supplementary File 2/2_Cluster marker top10 genes (28 clusters)/Cluster.27.VlnPlot_SC.png]

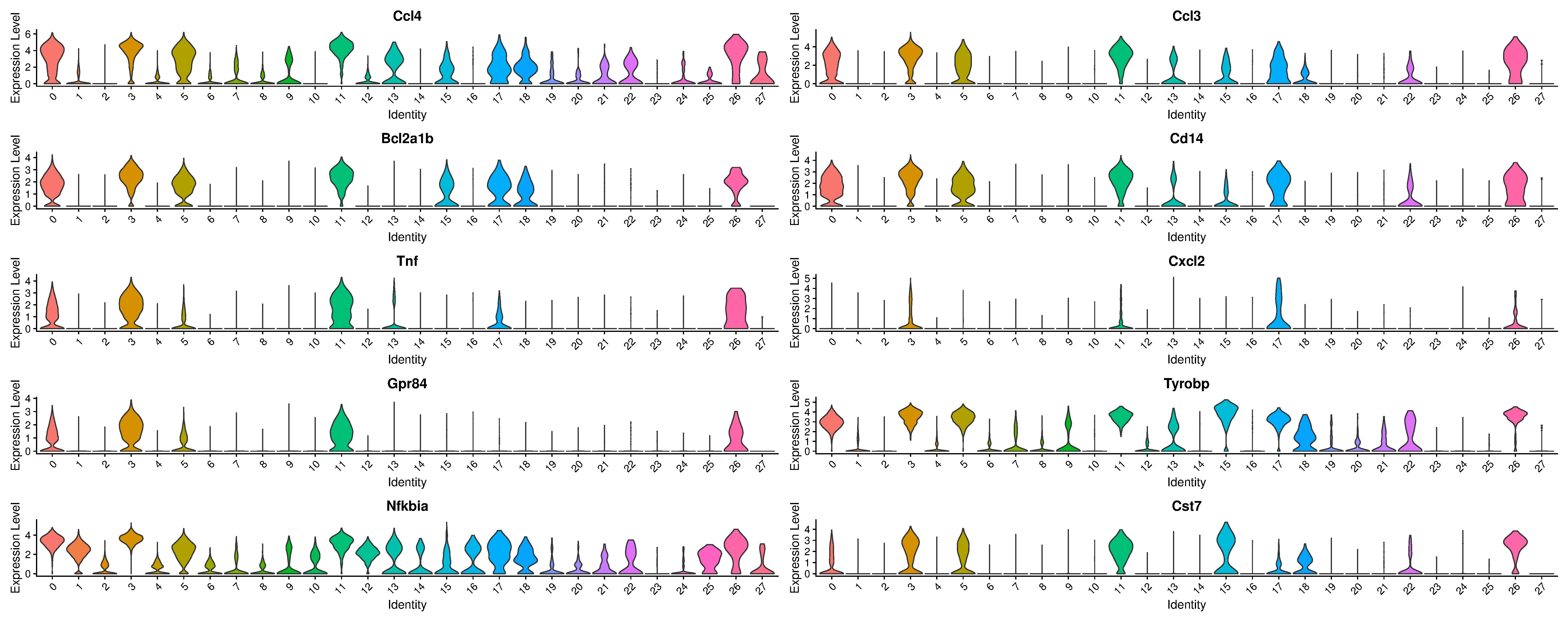

Supplement: Supplementary file 3 — Appendix S2. [file CNS-31-e70172-s002.zip › Supplementary File 2/2_Cluster marker top10 genes (28 clusters)/Cluster.3.VlnPlot_SC.png]

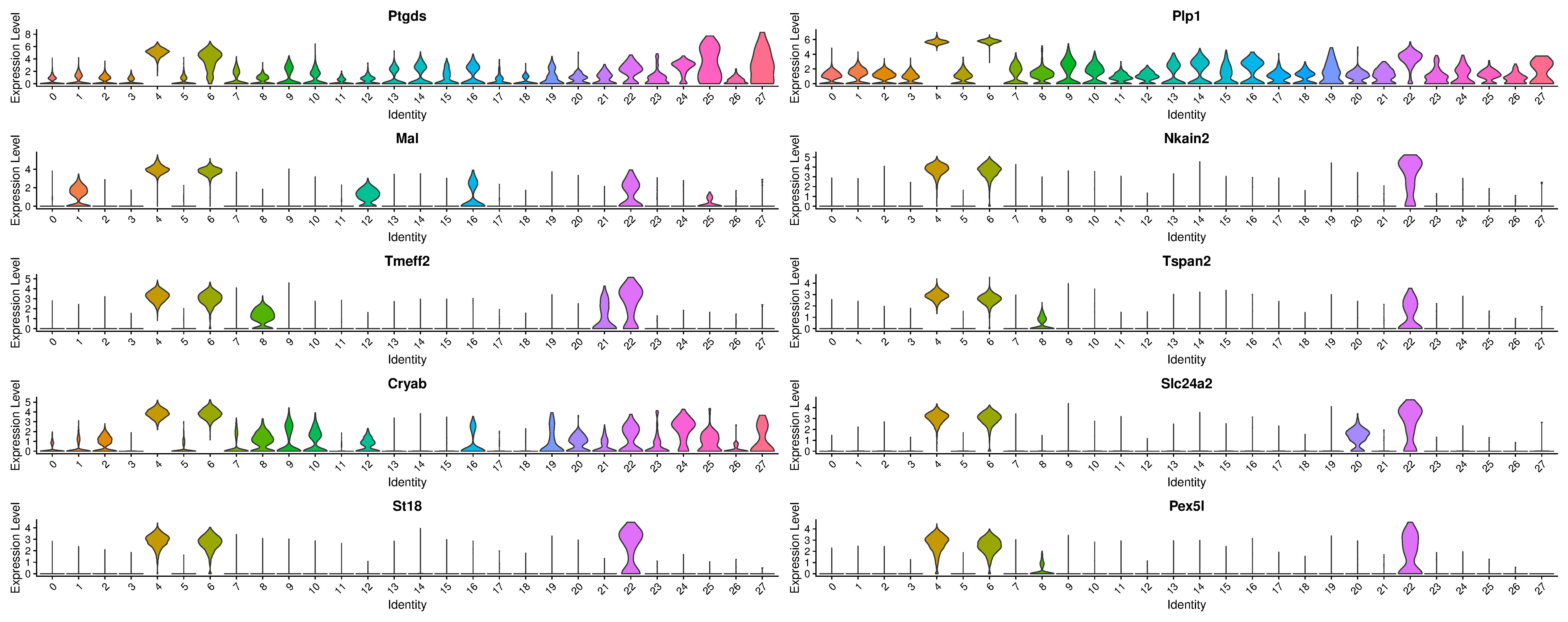

Supplement: Supplementary file 3 — Appendix S2. [file CNS-31-e70172-s002.zip › Supplementary File 2/2_Cluster marker top10 genes (28 clusters)/Cluster.4.VlnPlot_SC.png]

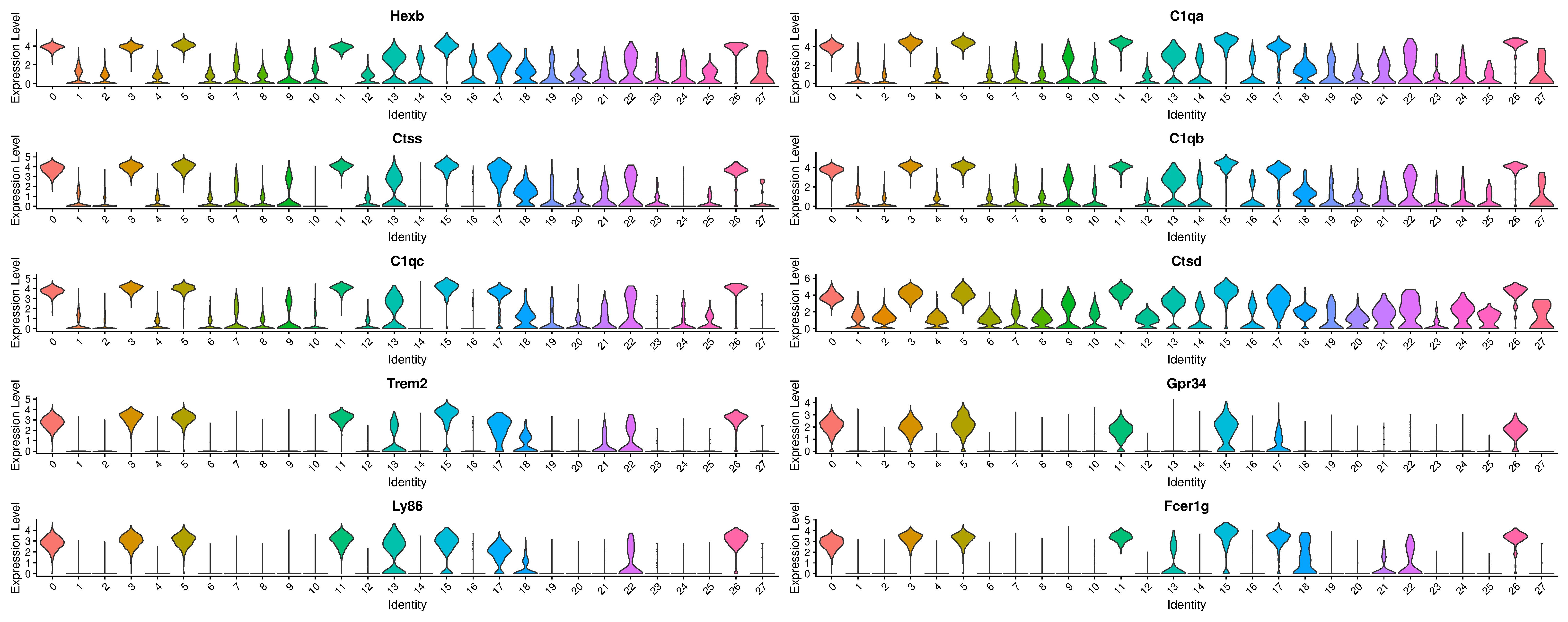

Supplement: Supplementary file 3 — Appendix S2. [file CNS-31-e70172-s002.zip › Supplementary File 2/2_Cluster marker top10 genes (28 clusters)/Cluster.5.VlnPlot_SC.png]

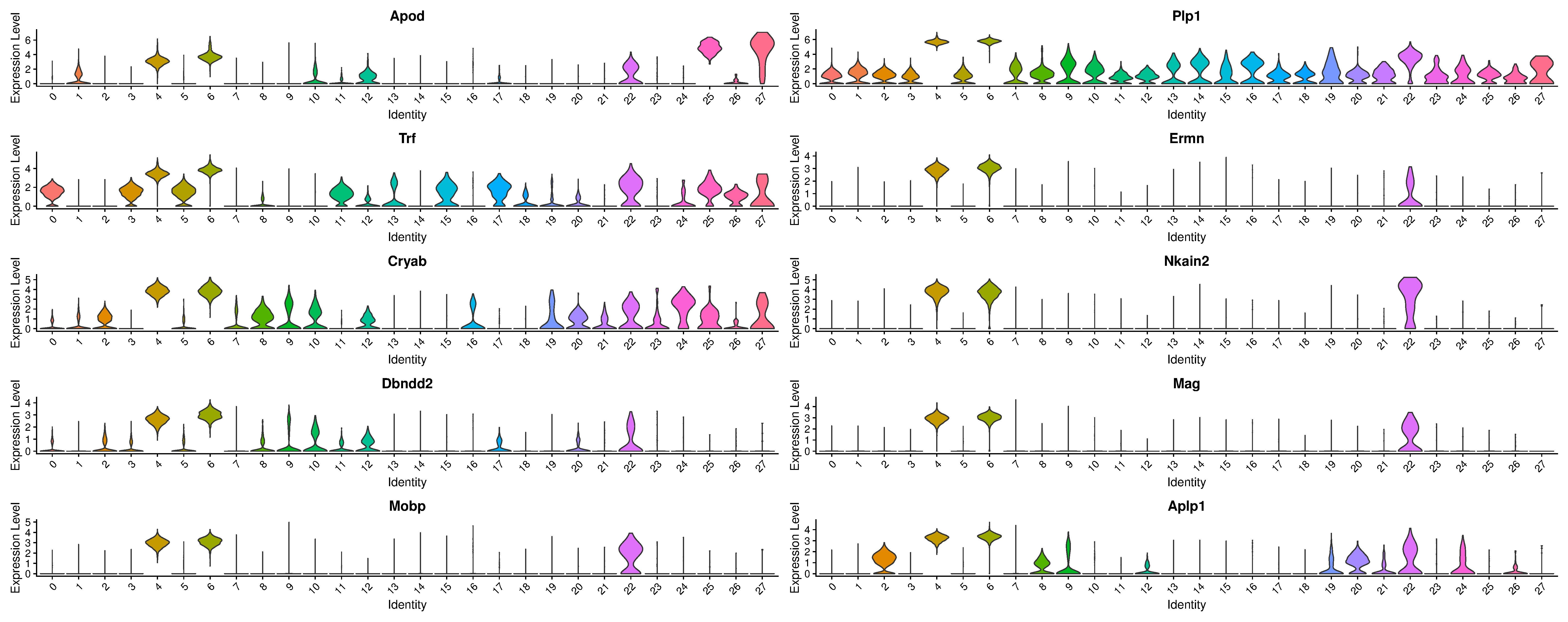

Supplement: Supplementary file 3 — Appendix S2. [file CNS-31-e70172-s002.zip › Supplementary File 2/2_Cluster marker top10 genes (28 clusters)/Cluster.6.VlnPlot_SC.png]

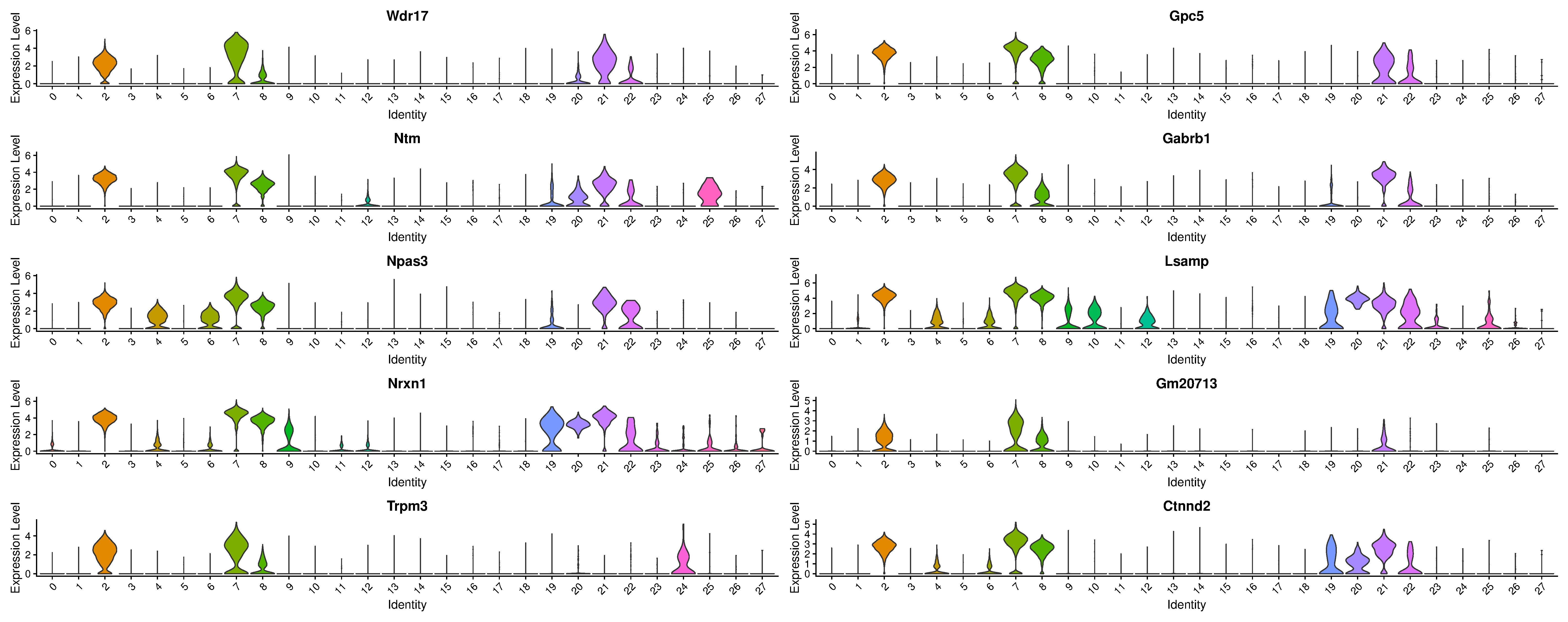

Supplement: Supplementary file 3 — Appendix S2. [file CNS-31-e70172-s002.zip › Supplementary File 2/2_Cluster marker top10 genes (28 clusters)/Cluster.7.VlnPlot_SC.png]

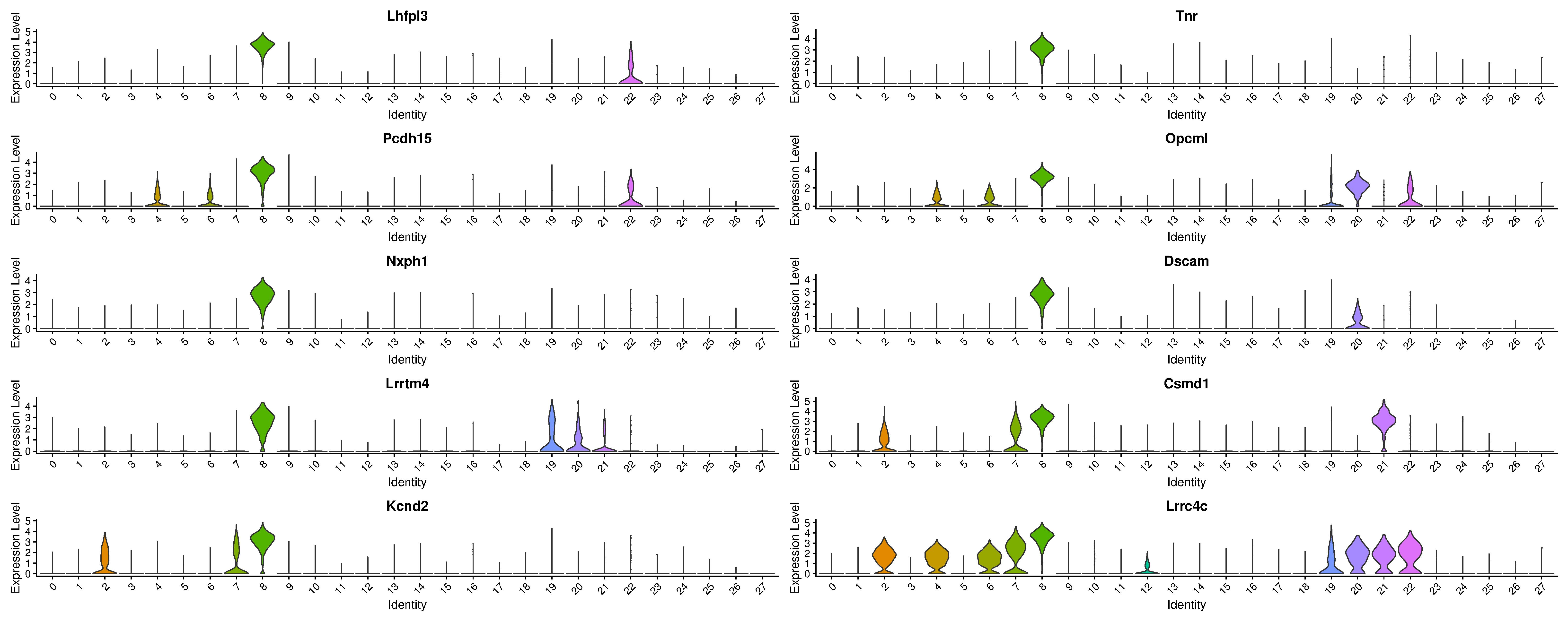

Supplement: Supplementary file 3 — Appendix S2. [file CNS-31-e70172-s002.zip › Supplementary File 2/2_Cluster marker top10 genes (28 clusters)/Cluster.8.VlnPlot_SC.png]

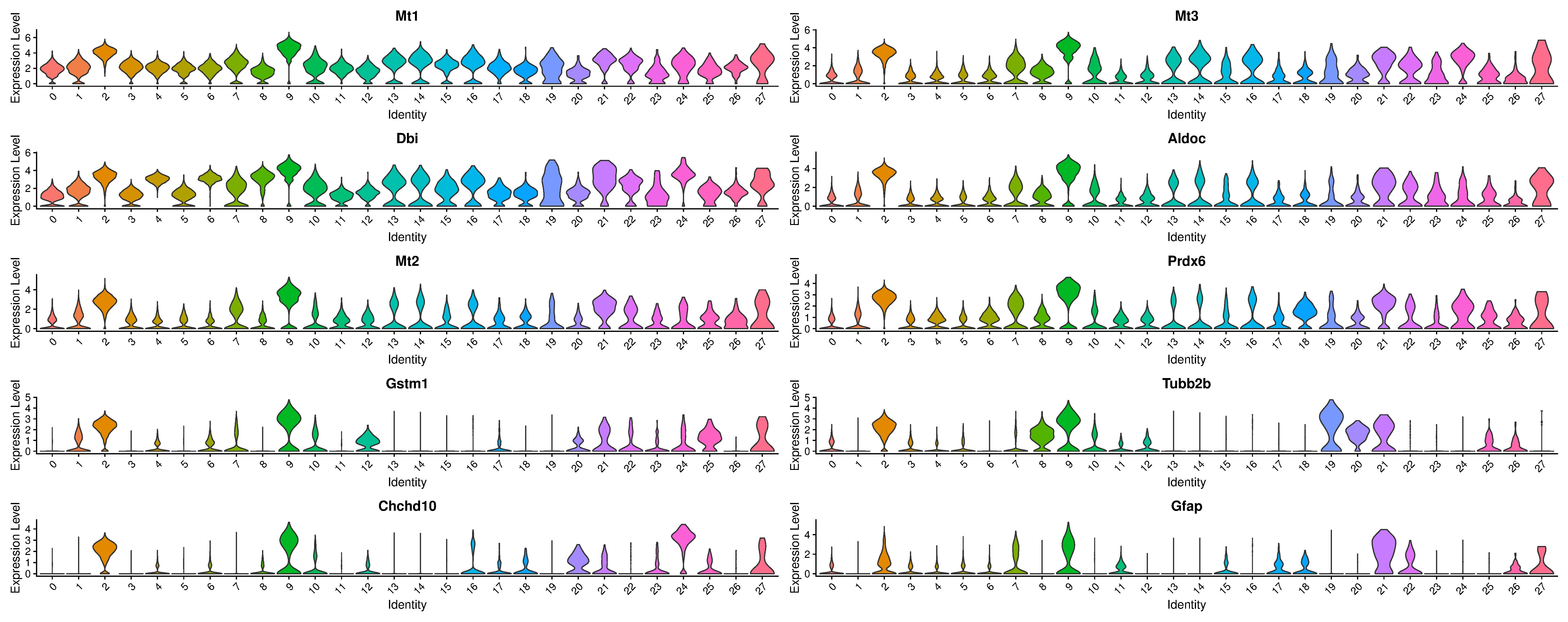

Supplement: Supplementary file 3 — Appendix S2. [file CNS-31-e70172-s002.zip › Supplementary File 2/2_Cluster marker top10 genes (28 clusters)/Cluster.9.VlnPlot_SC.png]

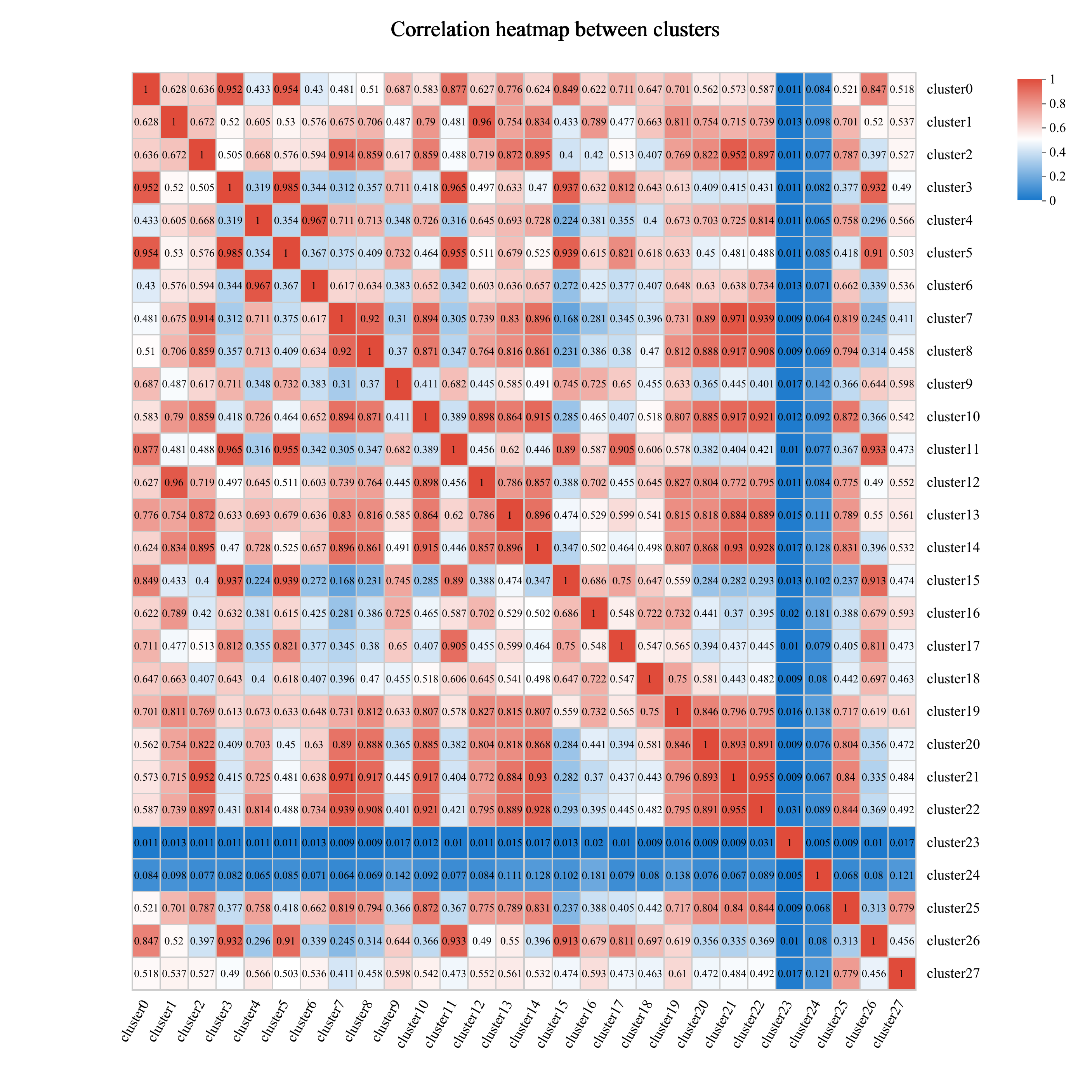

Supplement: Supplementary file 3 — Appendix S2. [file CNS-31-e70172-s002.zip › Supplementary File 2/3_Cluster proportion and correlation (28 clusters)/Correlation.png]
