# Supplementary material for: Integrated Mendelian Randomization and Single‐Cell Transcriptomics Analysis Identifies Critical Blood Biomarkers and Potential Mechanisms in Epilepsy
Source: CNS Neurosci Ther. 2025 Jan 3;31(1):e70172. doi: 10.1111/cns.70172 (PMC11702437; doi:10.1111/cns.70172)
Supplement: Supplementary file 2 — Appendix S1. [file CNS-31-e70172-s001.zip › Supplementary File 1/2_qPCR/Primer sequences_8 key genes.docx]

Sequence (5'->3')

**SSH2**

Forward primer TTATTCGAACGCCGGTCCAG

Reverse primer CTCTCGCTGATGAGCTGGTT

**MTX1**

Forward primer AAGACCAGCAATCCTTGGCA

Reverse primer AGATCGTAGTCGGCATTATACTCT

**GZMA**

Forward primer GGACTCCTGCAATGGGGATT

Reverse primer AAAAGAGGTGATGCCTCGCA

**DNMT1**

Forward primer CTGTCGTCTGCAACCTGCAA

Reverse primer CCAAGTCTTTGAGCCGCCTG

**CDC25B**

Forward primer TCCAGGGAGAGAAGGTGTCTTA

Reverse primer AGGATGGGTCGGATCACACT

**FGD3**

Forward primer TGGTCTTGGAAGAAGGCACC

Reverse primer CCTGTTGCCTAACACTGTTGC

**RAF1**

Forward primer CAACGCGTTCTCTCAACCAC

Reverse primer TTCCGCACATTGACCACAGT

**SH3BP5L**

Forward primer TCATGGCTGACAAAAATCGACT

Reverse primer GCCTCGTTCACCTTACAGGTA
