# Supplementary figures and images for: Integrated Mendelian Randomization and Single‐Cell Transcriptomics Analysis Identifies Critical Blood Biomarkers and Potential Mechanisms in Epilepsy (part 2 of 2)
Source: CNS Neurosci Ther. 2025 Jan 3;31(1):e70172. doi: 10.1111/cns.70172 (PMC11702437; doi:10.1111/cns.70172)

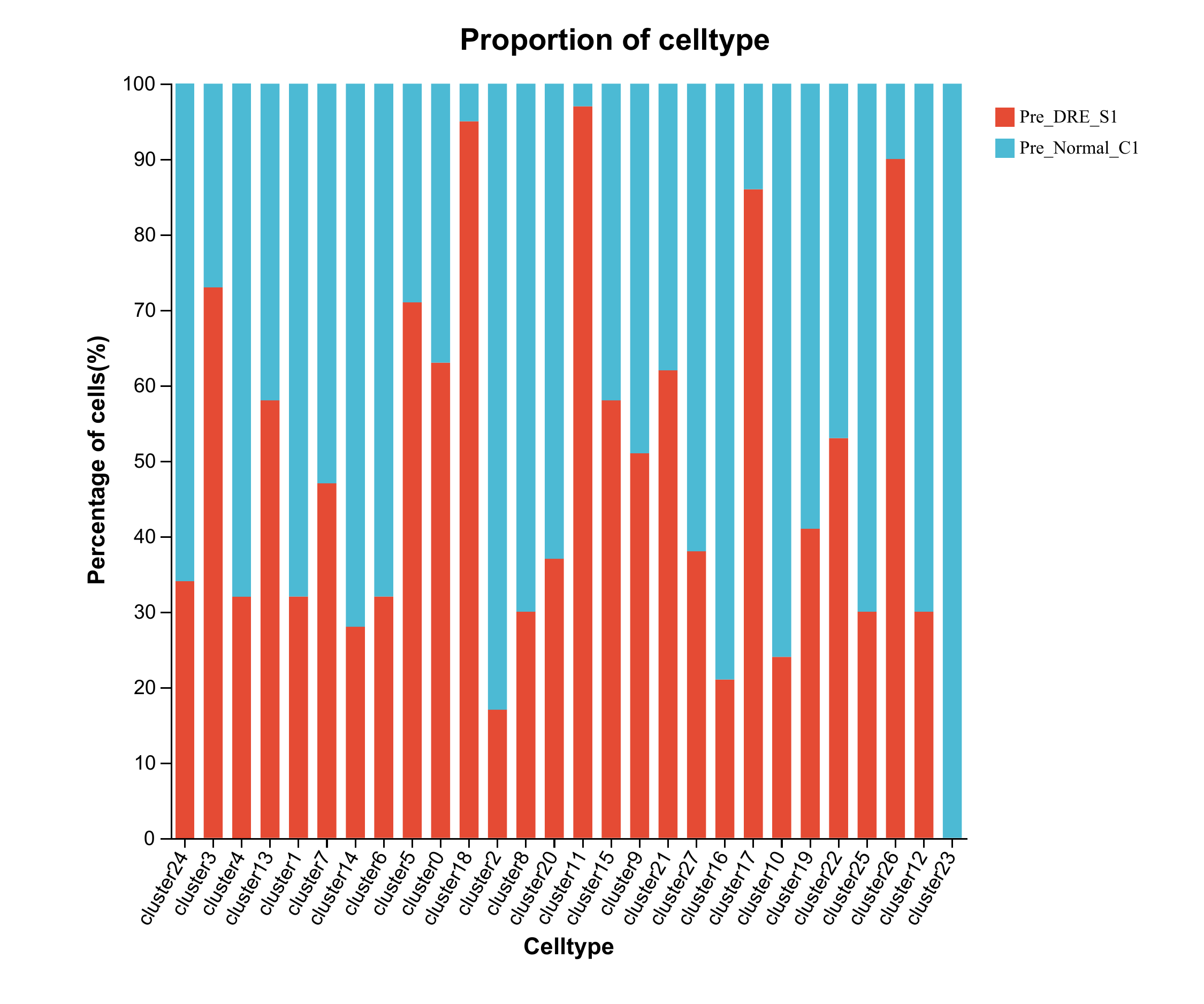

Supplement: Supplementary file 3 — Appendix S2. [file CNS-31-e70172-s002.zip › Supplementary File 2/3_Cluster proportion and correlation (28 clusters)/Proportion.png]
