# Supplementary material for: Integrated Mendelian Randomization and Single‐Cell Transcriptomics Analysis Identifies Critical Blood Biomarkers and Potential Mechanisms in Epilepsy
Source: CNS Neurosci Ther. 2025 Jan 3;31(1):e70172. doi: 10.1111/cns.70172 (PMC11702437; doi:10.1111/cns.70172)
Supplement: Supplementary file 4 — Data S1. [file CNS-31-e70172-s004.docx]

**Supplementary Files Checklist**

This article includes two supplementary files: Supplementary File 1 and Supplementary File 2. They are available on Zenodo (<https://zenodo.org/uploads/10826906>, DOI: 10.5281/zenodo.10826906).

**Supplementary File 1** contains three folders:

***1_Code and Data for Bio+MR***

This folder includes the bioinformatics analysis code and the raw data for Mendelian Randomization.

***2_qPCR***

This folder contains the validation experimental data for mRNA expression of the eight key genes in mouse hippocampus using qPCR. Specifically, it includes primer sequences, amplification curves, melting curves, and the raw data used for analysis and graphing.

***3_WB***

This folder contains the validation experimental data for protein expression of the eight key genes and the key upstream and downstream proteins of Fgd3 and Ssh2 in mouse hippocampus, as mentioned in Figure 6 and 8. The protein expression of the eight key genes was assessed using traditional WB, while proteins in the subsequent key pathways were analyzed using Digital WB.

***4******_Immunofluorescence***

This Excel spreadsheet contains the relative intensity data of immunofluorescence for proteins corresponding to the eight genes in the mouse hippocampal region.

**Supplementary File 2** contains four folders:

***1_Single Cell_Raw data statistics and quality assessment***

This folder contains the quality control data for single-cell analysis.

***2_Cluster marker top10 genes (28 clusters)***

This folder includes the top 10 marker genes for 28 clusters, representing their potential functions and roles.

***3_Cluster proportion and correlation (28 clusters)***

This folder contains heatmaps of the correlations between the 28 clusters and bar charts showing their proportions in the DRE and NC groups.

***4_Significant pathways***

This figure includes raw data for the pathways enriched in microglia from single-cell transcriptome analysis between DRE and NC groups.

**Data Sharing and Availability Statement**

While the supplementary files' data primarily ensure transparency and reproducibility of the research, our article cannot describe and discuss the contents of all supplementary files in detail. Therefore, if any researchers and teams refer to these contents, we hope our article will be appropriately cited. We plan to upload the complete raw data from the single-cell and spatial transcriptomics analysis to GEO or another international open-access platform before 2025. However, before then, we are also willing to share these data with any researchers or teams upon reasonable request.
